# Overcoming Methicillin-Resistant *Staphylococcus aureus* Infections by Disrupting Membrane Integrity and Inducing DNA Degradation with a Dual-Mechanism Fluorescent Phenanthro[9,10-d]imidazole Molecule

**DOI:** 10.34133/research.1242

**Published:** 2026-04-17

**Authors:** Ting Xu, Xiaoting Yan, ingting Wang, Liping Cui, Shangshang Qin, Ruige Yang, Hong Yao, Yong Guo

**Affiliations:** ^1^ Hunan Province Cooperative Innovation Center for Molecular Target New Drug Study, School of Pharmaceutical Science, Hengyang Medical School, University of South China, Hengyang 421001, Hunan Province, China.; ^2^ School of Pharmaceutical Sciences, Zhengzhou University, Zhengzhou 450001, Henan Province, China.; ^3^ College of Veterinary Medicine, Henan Agricultural University; Ministry of Education Key Laboratory for Animal Pathogens and Biosafety; Key Laboratory of Quality and Safety Control of Poultry Products, Ministry of Agriculture and Rural Affairs, Zhengzhou 450046, Henan Province, China.

## Abstract

The rise of multidrug-resistant bacteria, especially methicillin-resistant *Staphylococcus aureus* (MRSA), poses a serious threat to human health, necessitating an urgent need to develop innovative antibacterials with multifunctions for this. Here, focusing on integrating multi-antimicrobial mechanisms with visual monitoring, we report a novel phenanthro[9,10-d]imidazole fluorogen **9b**, outstanding for its antibacterial effects against *S. aureus* and various clinical MRSA isolates (minimum inhibitory concentration = 0.5 to 1 μg/ml) with rapid bactericidal activity, high membrane selectivity, and low susceptibility to drug resistance. Further investigation revealed its dual antimicrobial mechanisms of action by concurrently disrupting bacterial cell membranes and inducing bacterial DNA degradation to accelerate bacterial death. Ultraviolet–visible and fluorescence spectroscopy studies showed that the fluorescence intensity of **9b** was dramatically enhanced when it interacted with *S. aureus*. Notably, in vivo studies demonstrated that **9b** effectively treated both skin and thigh MRSA infections, showing a favorable safety profile and superior efficacy compared to the first-line antibiotic vancomycin. The “dual-mechanism” fluorescent phenanthro[9,10-d]imidazole fluorogen **9b** holds promise as a candidate for developing innovative antibacterial agents that enable simultaneous real-time diagnosis and treatment.

## Introduction

The abuse and misuse of antibiotics have led to rising bacterial resistance, posing important environment and public health risks, particularly due to the rise of methicillin-resistant *Staphylococcus aureus* (MRSA) [1–3]. MRSA, a prominent example of “Superbacteria”, has been regarded to be the most common pathogens responsible for hospital-acquired and community-acquired infections [4,5]. It causes infections like skin and soft tissue infections, bloodstream infections, and systemic infections thereby affecting multiple organs [6–8]. MRSA contains the SCC*mec* gene cassette, which encodes the altered *β*-lactam target proteins PBP2a/2c, along with multiple drug-resistant plasmids [9]. Additionally, it demonstrates intrinsic resistance mechanisms such as active efflux systems, biofilm formation, and persistence, often leading to multidrug resistance or even extensively drug-resistant profiles [10–13]. Moreover, what of particular concern is the lack of the usage monitoring for antibacterial agents, which leads to faster evolution and spread of resistance genes, making many first-line antibiotics, such as vancomycin, daptomycin, and linezolid, begin to be ineffective [6,14], thereby causing further overuse and finally severe poisoning due to antibiotic residues in food [15]. These complicate clinical management, leading to high morbidity and mortality rates among infected patients [12,16]. Therefore, it is urgent to explore nontoxic, nonresidual, and multifunctional antibacterial strategies to fight against, while simultaneously monitoring, pathogenic bacteria, especially drug-resistant MRSA infections. Fluorescently active, green, and multitargeted anti-MRSA agents are possible solutions [17,18].

Antibacterials with multiple targets or mechanisms have been proved to be more effective to bacterial resistance [19,20]. Among them, combinations of bacterial cell membrane and DNA targeting have been recently proved to be efficient [21,22]. For bacterial cell membrane, the structure and composition are relatively stable, which makes it hard to produce drug resistance through mutation [23], while the differences from the nuclear membrane of human normal eukaryotic cells to bacterial nucleoid can provide excellent antimicrobial selectivity [24]. The bacterial cell membrane contains unique negative components (such as lipopolysaccharide), which can be selectively bound and destroyed by special natural or artificial amphiphilic cation materials [21,25,26]. For bacterial DNA, unlike the normal eukaryotic cell DNA protected by the nuclear membrane, the bacteria nucleoid DNA is naked, which makes it more vulnerable to be attacked by those molecules with direct DNA targeting ability, so that some DNA intercalators can bind to DNA and inhibit the synthesis of nucleic acid, thereby playing an antibacterial activity [27,28].

In recent years, membrane-targeting antibacterials, represented by antimicrobial peptides and their analogs, have garnered widespread attention both domestically and internationally due to their broad-spectrum activities and low propensity for inducing resistance [26,29–31]. However, the preparation process of these natural biological macromolecules or synthetic analogs is usually complex, difficult, and expensive. In addition, the existing methods of infection surveillance are mainly to observe clinical symptoms, blood tests, and histopathological examinations [32], which are complex and lead to poor patient compliance, and it is difficult to truly monitor the rational use of antibacterial agents. Recent studies have attempted to additionally introduce fluorescent activity into antibacterial agents to help online visual monitoring for antibacterial processes [33,34]. Ideally, the conjugation of fluorophores to antibacterials should not alter the binding ability to its original targets or pharmacokinetics. However, factors such as the steric hindrance and lipophilicity of the fluorophore, along with the functionalization sites on the antibacterials, often do impact these factors [35,36]. Consequently, integrating fluorescence and potent antimicrobial effects simultaneously poses enormous challenges.

**Fig. 1.**
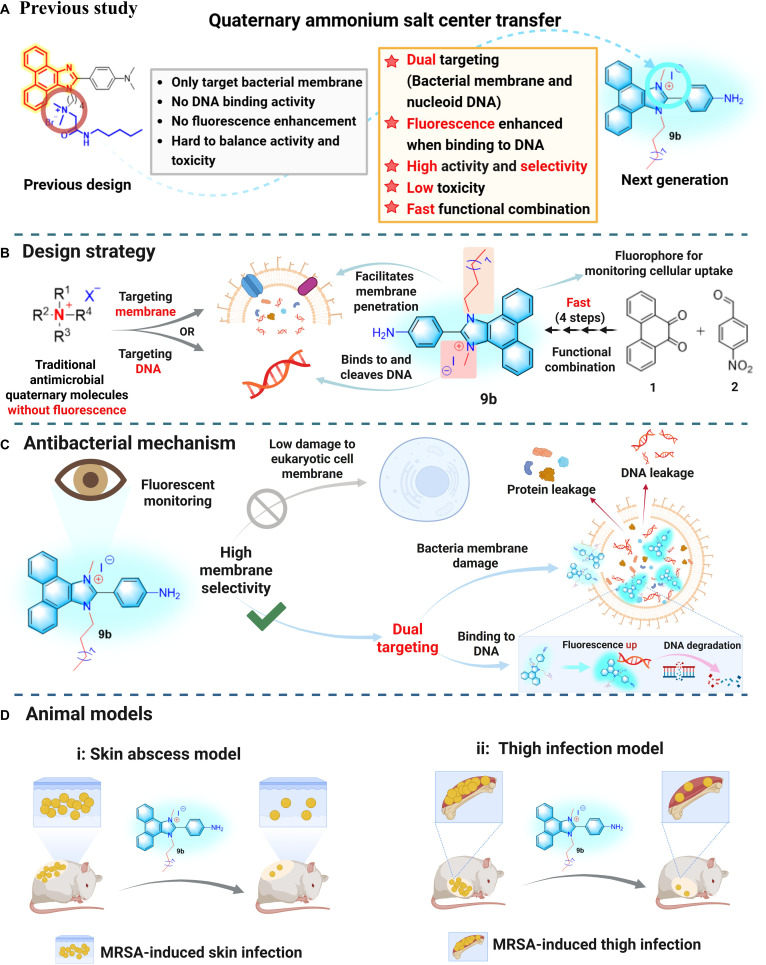
(A) Previous study of a phenanthrenequinone-imidazole compound III13. (B) Design of a fluorescent phenanthro[9,10-d]imidazole molecule 9b with anti-methicillin-resistant *Staphylococcus aureus* (MRSA) activity. (C) The proposed mechanisms of compound9b. (D) Compound 9b exhibits good therapeutic potential in treating the MRSA-induced skin and thigh infections, respectively.

In our previous work, we identified that the phenanthrenequinone-imidazole quaternary ammonium compound **III13** (Fig. 1A), a pseudo-natural product, possesses notable fluorescence properties and demonstrates some antibacterial activity [33]. However, unfortunately, **III13** did not show obvious changes in fluorescence intensity during antibacterial processes, which provides no insight into its antibacterial mode and monitoring ability through further visualization of its fluorescence properties (Fig. 1A). Meanwhile, its bactericidal activity is solely attributed to the bacterial cell membrane-targeting mechanism [37], which makes it difficult to adjust its selectivity to balance antibacterial activity and toxicity to normal cells. However, in theory, the quaternary ammonium structure should have the potential to both target the bacterial cell membrane and interact with DNA [37,38]. Therefore, there is still a broad space for **III13**-based antimicrobial molecules to develop. To this end, we attempted to modify the phenanthrenequinone-imidazole scaffold by introducing a long alkyl chain to increase its affinity to bacterial cell membranes, and transfer the quaternary ammonium structure center to the highly conjugated hydrophobic scaffold to retain the bacterial membrane-targeting ability while enhancing the binding potential to bacterial nucleoid DNA (Fig. 1A and B). The goal was to design and screen phenanthrenequinone-imidazole compounds with both strong fluorescence characteristics and potent bactericidal activity (Fig. 1B). Fortunately, a phenanthro[9,10-d]imidazole fluorescent molecule **9b** was identified, exhibiting both strong fluorescence activity and remarkable antibacterial properties (Fig. 1C). This study investigated the in vitro antibacterial effects, membrane selectivity, bactericidal properties, dual membrane-targeting and DNA-binding mechanism, and the in vivo anti-MRSA efficacy of compound **9b** across multiple models (Fig. 1D). These findings provide important implications for the creation of innovative fluorescent antibacterial molecules.

## Results and Discussion

### Synthesis of fluorescent phenanthro[9,10-d]imidazole compounds

**Fig. 2.**
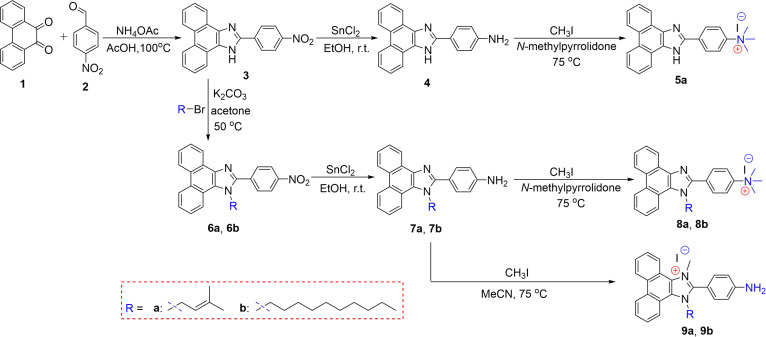
Synthetic routes of fluorescent phenanthro[9,10-d]imidazole compounds 5a, 8a, 8b, 9a, and 9b. r.t., room temperature.

As shown in Fig. 2, we first synthesized the intermediate **3** from 9,10-phenanthroquinone (**1**) and 4-benzenedicarboxaldehyde (**2**) in the presence of NH_4_OAc by the cyclization reaction, followed by the reduction of aromatic nitro group to amine and then the quaternization reaction of compound **4** with methyl iodide to obtain *N,N,N*-trimethyl-4-(1*H*-phenanthro[9,10-d]imidazol-2-yl)benzenaminium iodide (**5a**). Subsequently, to increase the hydrophobicity of **5a**, we carried out an alkyl substitution at the *N*-1 position of intermediate **3** and then obtained compounds **8a** and **8b** using a similar protocol for the synthesis of **5a**. Interestingly, we found that the use of acetonitrile as a solvent in the quaternization of compounds **7a** and **7b** gave products **9a** and **9b** instead of **8a** and **8b**. It has been widely reported that amino groups have a great influence on the antibacterial activity of compounds [39,40].

**Fig. 3.**
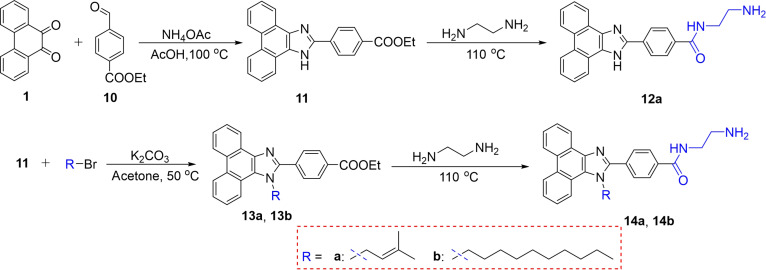
Synthetic routes of fluorescent phenanthro[9,10-d]imidazole compounds 12a, 14a, and 14b.

To further investigate the effect of an amino group on phenanthro[9,10-d]imidazole compounds as well as to increase their structural diversity, we also prepared compound **12a** using 9,10-phenanthroquinone (**1**) and ethyl 4-formylbenzoate (**10**) as the raw material by cyclization and amination reactions. Similarly, we further synthesized compounds **14a** and **14b** with alkyl substitution at the *N*-1 position of the imidazole ring (Fig. 3).

**Fig. 4.**
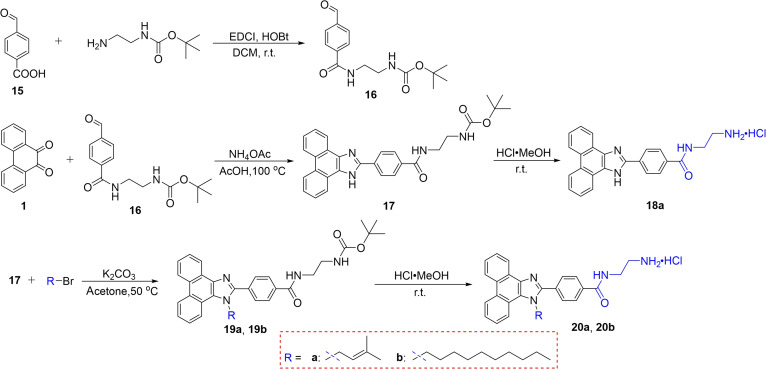
Synthetic routes for fluorescent phenanthro[9,10-d]imidazole compounds of hydrochlorides 18a, 20a, and 20b. r.t., room temperature.

Additionally, to increase the water solubility of compounds **12a**, **14a**, and **14b** in favor of evaluation of their antibacterial activities, we further prepared hydrochlorides **18a**, **20a**, and **20b** of compounds **12a**, **14a**, and **14b** (Fig. 4). The structures of all novel phenanthro[9,10-d]imidazole compounds **5a**, **8a**, **8b**, **9a**, **9b**, **12a**, **14a**, **14b**, **18a**, **20a**, and **20b** were characterized by ^1^H/^13^C nuclear magnetic resonance (NMR), and high-resolution mass spectrometry (HRMS) (see the Supplementary Materials).

### In vitro antibacterial and hemolytic assays

In vitro antibacterial and hemolytic properties were assessed for the synthesized phenanthro[9,10-d]imidazole derivatives. Their antibacterial efficacy against a panel of gram-positive and gram-negative bacterial strains was initially examined using a 2-fold serial dilution method following Clinical and Laboratory Standards Institute guidelines [41]. The results indicated that compound **9b** exhibited pronounced antibacterial activity against both G+ and G− bacteria, with minimum inhibitory concentration (MIC) values ranging from 0.5 to 64 μg/ml, showing particularly strong activity against *S. aureus* ATCC 29213. To further evaluate cytotoxicity and membrane selectivity, we conducted hemolytic assays to determine the half hemolytic concentration (HC_50_) values of these compounds. Compound **9b** displayed minimal hemolytic activity toward sheep erythrocytes and demonstrated excellent selectivity for *S. aureus*, yielding a high selectivity index of 744.2 (Table 1).

**Table 1.** In vitro antibacterial (MIC, μg/ml) and hemolytic activities (HC_50_, μg/ml) of compounds 5a, 8a, 8b, 9a, 9b, 12a, 14a, 14b, 18a, 20a, and 20b

Compd.	G+ bacteria			G− bacteria			HC_50_ (μg/ml)	SI ^a^
	*S. a* ^b^	*M. l* ^c^	*S. s* ^d^	*E. c* ^e^	*K. p* 18-227 ^f^	*K. p* 18-29 ^g^		
**1**	4	8	8	>64	>64	>64	>320	>80
**5a**	16	32	16	>128	>128	>128	>640	>40
**8a**	8	16	16	16	32	32	247.2	>30.9
**8b**	32	32	64	>128	>128	>128	167.8	5.2
**9a**	1	2	4	8	32	16	318.6	318.6
**9b**	0.5	0.5	4	16	64	64	372.1	744.2
**12a**	>128	>128	>128	>128	>128	>128	>640	>5
**14a**	>128	>128	>128	>128	>128	>128	>640	>5
**14b**	>128	>128	>128	>128	>128	>128	>320	>5
**18a**	>128	>128	>128	>128	>128	>128	>80	>5
**20a**	>128	>128	32	>128	>128	>128	>640	>5
**20b**	>128	>128	>128	>128	>128	>128	>640	>5
**VAN** ^h^	2	0.5	0.5	ND	ND	ND	ND	ND
**MEM** ^i^	ND ^j^	ND	ND	0.0625	8	0.0625	ND	ND

MIC, minimum inhibitory concentration; HC_50_, half hemolytic concentration

^a^ SI: Selectivity index (HC_50_/MICs of *S. aureus*).

^b^ *S. a*: *Staphylococcus aureus* ATCC 29213.

^c^ *M. l*: *Micrococcus luteu*.

^d^ *S. s*: *Streptococcus suis*.

^e^ *E. c*: *Escherichia coli* ATCC 25922.

^f^ *K. p* 18-227: *Klebsiella pneumoniae* 18-227.

^g^ *K. p* 18-29: *Klebsiella pneumoniae* 18-29.

^h^ **VAN**: Vancomycin.

^i^ **MEM**: Meropenem.

^j^ ND: not determined. This experiment was repeated 4 times.

Encouraged by the good antibacterial activity of compounds **5a**, **8a**, **8b**, **9a**, and **9b** against *S. aureus*, we further screened their antibacterial activities against 10 clinical MRSA isolates. Interestingly, compound **9b** also displayed robust antibacterial activity against these clinical MRSA isolates with MIC values in the range of 0.5 to 1 μg/ml (Table 2), which is comparable to the first-line drug vancomycin. The antibacterial activity screening results of **5a**, **8a**, **8b**, **9a** and **9b** (Table 2) showed that the MIC of **9a** and **9b** was 8-fold or 32-/64-fold higher than that of the reference compounds **8a** and **8b**, suggesting that quaternization occurs at position *N*-3 of the imidazole ring to yield a more active compound than at the amino position of the benzene ring.

**Table 2.** In vitro antibacterial activities against clinical MRSA strains of compounds 5a, 8a, 8b, 9a, and 9b

Clinical MRSA isolates	MIC (μg/ml)					
	5a	**8a**	**8b**	**9a**	**9b**	**VAN** ^a^
MRSA-11	32	8	32	1	1	0.5
MRSA-13	16	8	32	2	1	0.5
MRSA-14	16	8	32	1	0.5	0.5
MRSA-16	16	8	32	1	0.5	1
MRSA-17	16	8	32	1	1	1
MRSA-19	16	8	32	1	0.5	0.5
MRSA-20	16	8	32	1	0.5	1
MRSA-21	16	8	32	1	1	0.5
MRSA-22	16	8	32	1	1	0.5
MRSA-23	16	8	32	1	0.5	1

MRSA, methicillin-resistant *Staphylococcus aureus*; MIC, minimum inhibitory concentration

^a^ **VAN**: vancomycin. The experiment was repeated 3 times.

### Time-dependent killing and resistance development assays

**Fig. 5.**
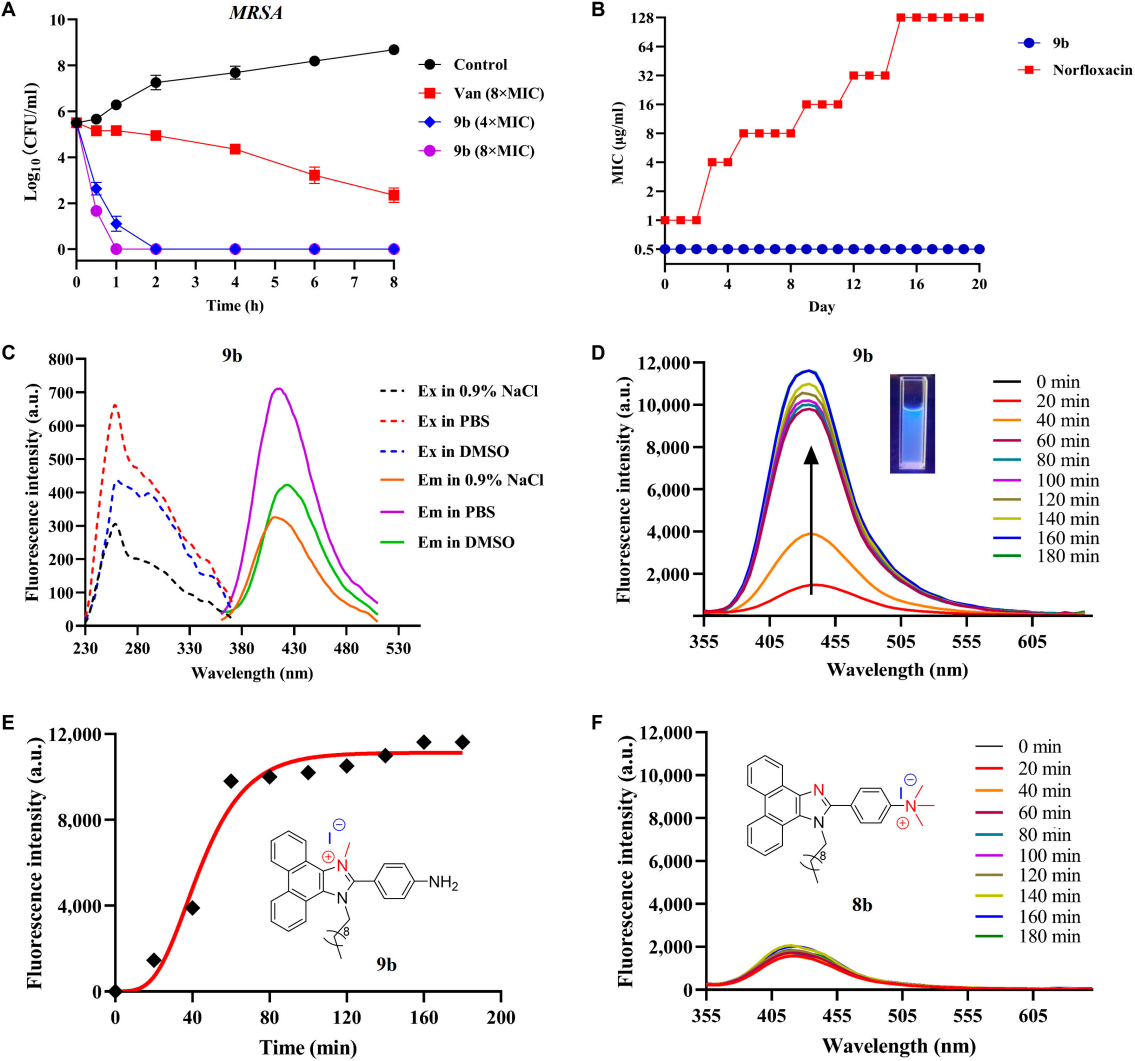
(A) Time-kill kinetics of 9b against MRSA-4. (B) Bacterial resistance study of 9b against *S. aureus* ATCC 29213. Data are presented as means ± SD (*n* = 3 biological replicates). (C) Excitation (ex) and emission (em) spectra of 9b in NaCl, phosphate-buffered saline (PBS), and dimethyl sulfoxide (DMSO) (10 μM). (D) Fluorescence intensity of 9b after treatment of *S. aureus* ATCC 29213 (*λ*_ex_ = 251 nm). (E) Plot of fluorescence intensity at 435 nm against incubation time (0 to 180 min). (F) Fluorescence intensity of 8b after treatment of *S. aureus* ATCC 29213 (*λ*_ex_ = 251 nm). a.u., arbitrary units.

Given the excellent in vitro anti-MRSA activity and low hemolytic effect, **9b** was selected as a candidate for further studies. To evaluate its bactericidal properties, we first investigated the time-kill kinetics against *S. aureus* ATCC 29213 and clinical MRSA isolates. As a result, **9b** was effective in killing bacteria, and it completely eliminated *S. aureus* ATCC 29213 and MRSA-16 in exponential growth phase within 0.5 and 2 h at 8× MIC, respectively, superior to the reference drug vancomycin (Fig. 5A), and also displayed impressive bactericidal properties even at 4× MIC on completely killing eradicated *S. aureus* ATCC 29213 and MRSA-16 within 0.5 and 2 h, respectively (Fig. 5A). Additionally, the development of resistance to compound **9b** against *S. aureus* ATCC 29213 was investigated at sub-MIC (1/2 MIC). Figure 5B shows that the MIC values of **9b** remained unchanged within 20 passages of *S. aureus* ATCC 29213 treated by the sub-MIC level of **9b**, indicating that **9b** is not susceptible to induce resistance to *S. aureus* ATCC 29213. In contrast, development of resistance to the reference drug norfloxacin was apparently observed, with a 128-fold increase in MIC values at the 20th passage. Here, we hypothesized that the less susceptibility of compound **9b** to resistance may be due to its rapid bactericidal capacity and membrane-targeting mode.

### Antimicrobial mechanism studies

#### Photophysical properties of 9b and its fluorescence intensity change upon interaction with bacteria

After evaluation of the anti-MRSA performance of compound **9b**, its photophysical properties were investigated in 0.9% NaCl, phosphate-buffered saline (PBS), and dimethyl sulfoxide (DMSO), respectively. The fluorescence intensity of **9b** in PBS at both excitation and emission wavelengths exceeded that in 0.9% NaCl and DMSO (Fig. 5C). Then, based on the spectral characteristics of compound **9b**, quinine sulfate was selected as the reference standard to determine its fluorescence quantum yield, which was calculated to be only 3.11% (Fig. S1). This result is consistent with expectations, as **9b** should exhibit very weak fluorescence in the absence of binding to bacteria or bacterial DNA, thereby avoiding interference with diagnostic interpretation in theranostic applications or monitoring the fluorescence of cellular uptake and investigating the mode of antimicrobial action. Subsequently, we further confirmed that the fluorescence quantum yields of **9b** enhanced a lot when interacting with *S. aureus* ATCC 29213 by fluorescence spectroscopy. Spectral monitoring displayed that **9b** obviously interacted with *S. aureus* ATCC 29213 cells within 20-min incubation (Fig. 5D). The fluorescence signal gradually increased and converged to the saturation point within 60 min (Fig. 5E), which was about 17 times higher than the fluorescence intensity of **9b** without bacteria (Fig. 5D and E). To further evaluate whether **9b** maintained its fluorescence characteristics under biologically relevant conditions, we investigated its photophysical behavior in environments with varying pH, ionic strength, and solvent polarity (Fig. S2A to C). The results showed that the fluorescence intensity of **9b** remained relatively stable across the physiological pH range (pH 6 to 8), indicating minimal pH-dependent perturbation. An increase in ionic strength (NaCl, 0% to 0.9%) and a decrease in solvent polarity (EtOH, 0% to 100%) led to moderate enhancement of fluorescence intensity. However, these changes were substantially weaker than the fluorescence enhancement observed upon bacterial binding, suggesting that the signal amplification in infected tissues is unlikely to arise solely from matrix effects. In addition, **9b** exhibited acceptable photostability in PBS (Fig. S2D). Under continuous excitation, only a gradual decrease in fluorescence intensity was observed over 40 min, indicating resistance to rapid photobleaching under experimental conditions. Collectively, these results support the robustness of **9b** fluorescence under biologically relevant conditions and strengthen its potential for theranostic applications. Moreover, we further investigated the fluorescence intensity of compound **8b** with the quaternary ammonium center at the amino group (–NH_2_) after incubation with MRSA and found that the interaction of **8b** with MRSA cells was very slight even after 3 h of incubation (Fig. 5F). These results indicated that compound **9b** may interact with the bacterial DNA thereby enhancing its fluorescence intensity. Therefore, it is hypothesized that the quaternary ammonium structure on the imidazole ring might enhance the interaction of **9b** with bacterial DNA compared to **8b**, thereby improving the antibacterial activity of **9b**.

#### Investigation of the interaction between compound 9b and bacterial DNA

**Fig. 6.**
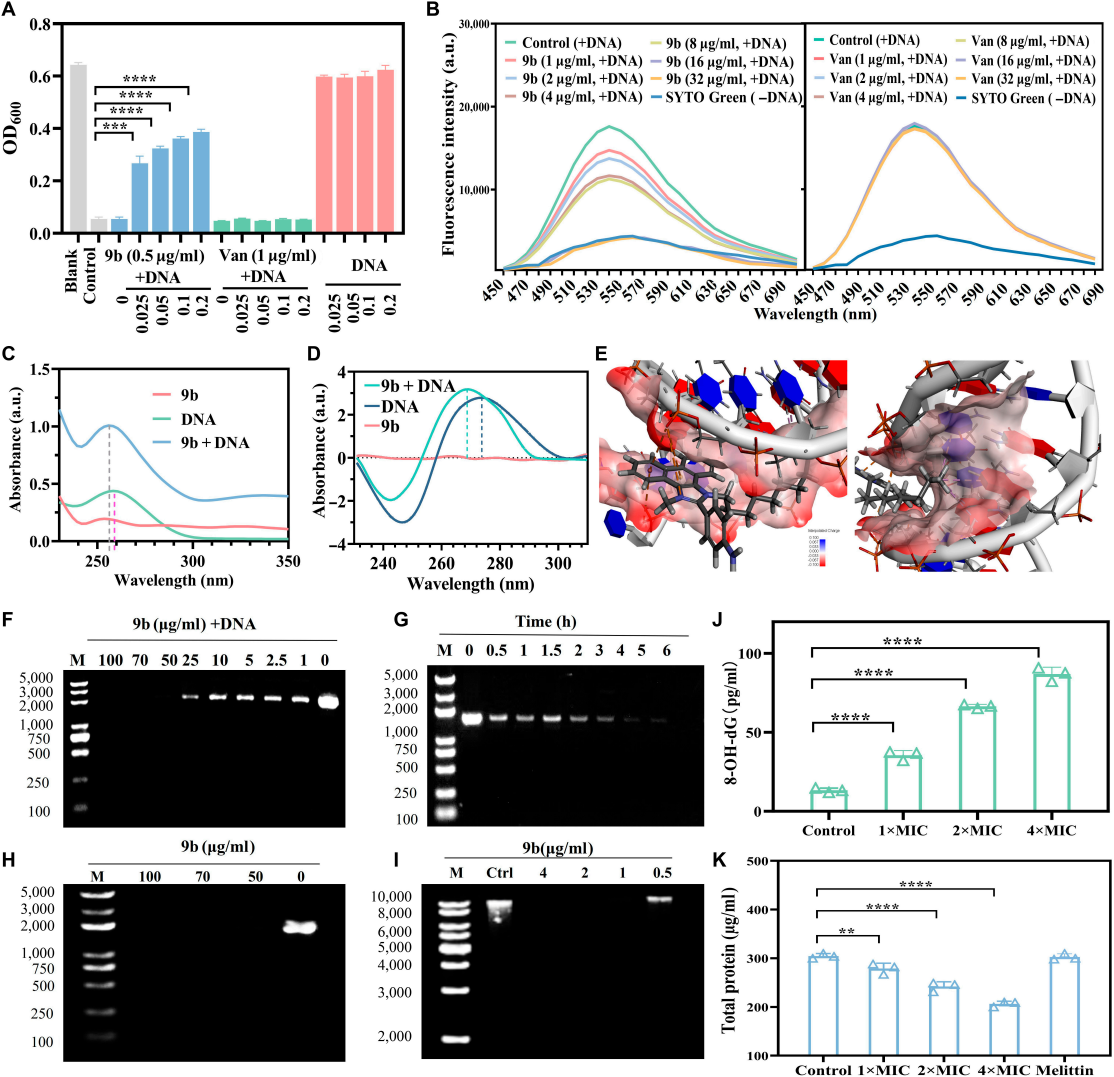
Antibacterial mechanism of compound 9b against the clinical MRSA-16 isolate. (A) Evaluation of the influence of exogenous bacterial DNA on the antibacterial efficacy of 9b. (B) Fluorescence quenching of the DNA–dye complex following the addition of 9b using SYTO Green, a minor groove–binding probe. Vancomycin served as the control. (C) Ultraviolet (UV) absorption spectra of DNA recorded before and after exposure to compound 9b, with black and red dashed lines marking the characteristic absorption peaks prior to and following treatment. (D) Circular dichroism (CD) spectra illustrating the structural changes of DNA in the absence or presence of 9b. (E) Molecular docking simulation displaying the interaction between compound 9b and dsDNA (CGCGAA TTCGCG)_2_ (PDB: 4U8A). (F) Agarose gel electrophoresis of pUC19 plasmid DNA treated with various concentrations of compound 9b. (G) Time-dependent electrophoretic analysis of pUC19 plasmid DNA incubated with 9b (50 μg/ml). (H) Agarose gel electrophoresis of pUC19 DNA purified after treatment with 9b. (I) Electrophoretic profiles of genomic DNA extracted from methicillin-resistant *Staphylococcus aureus* (MRSA) cells following incubation with compound 9b. Lane M: 1-kb DNA marker; lane 1: genomic DNA from untreated MRSA; lanes 2 to 5: genomic DNA from MRSA exposed for 3 h to lysogeny broth (LB) medium supplemented with 4, 2, 1, or 0.5 μg/ml 9b. (J) The content of 8-hydroxy-2′-deoxyguanosine (8-OH-dG) in MRSA after treatment with different concentrations of compound 9b for 2 h. (K) Total protein content of MRSA after 2 h of exposure to increasing concentrations of 9b. Data are presented as means ± SD (*n* = 3 biological replicates). Statistical significance was determined by 1-way analysis of variance (ANOVA) with Tukey’s multiple-comparison test: **P < 0.01, ***P < 0.001, ****P < 0.0001. a.u., arbitrary units.

To confirm the interaction between compound **9b** and MRSA’s DNA, we evaluated the effect on the antibacterial activity of **9b** by exogenous addition of genomic DNA (from MRSA-16). The antibacterial activity of **9b** could be suppressed by the MRSA genomic DNA, whereas the reference drug vancomycin displayed no such effect upon DNA addition (Fig. 6A). This result indicated a strong interaction between **9b** and MRSA genomic DNA, but no such effect was observed in the non-DNA-targeted vancomycin. Further dye-displacement experiments confirmed the binding mode of **9b** with DNA. Fluorescence competition assays using SYTO Green showed that the fluorescence intensity of the DNA–dye complex progressively decreased with increasing concentrations of **9b**, indicating that **9b** competes with SYTO Green for the minor groove binding site on DNA (Fig. 6B). Consistently, ultraviolet (UV) absorption spectra revealed a blue shift of DNA characteristic peaks after **9b** treatment (Fig. 6C), while circular dichroism spectra exhibited enhanced positive ellipticity and reduced negative signals, indicating obvious conformational alterations of DNA upon binding with **9b** (Fig. 6D). Molecular docking analysis supported these findings, showing that **9b** fits into the DNA minor groove with charge–charge interactions (Fig. 6E). To further assess the stability of the docking-predicted minor-groove binding mode under dynamic conditions, we performed molecular dynamics (MD) simulations. Starting from the docked conformation, a 100-ns MD trajectory was conducted. Root-mean-square deviation (RMSD) analysis demonstrated the overall structural evolution of the **9b**–DNA complex throughout the simulation. It reached a stable plateau phase after an initial equilibration period, with no dissociation observed (Fig. S3A). Consistent with the RMSD results, the radius of gyration exhibited a decreasing trend after approximately 60 ns, suggesting that the system gradually approached a more compact conformational state during equilibration (Fig. S3B). Hydrogen bond analysis revealed only 1 to 2 transient hydrogen bonds between **9b** and DNA over the course of the simulation, consistent with the docking results indicating that charge–charge interactions dominated the interaction (Fig. S3C). In addition, the solvent-accessible surface area gradually increased and then stabilized, suggesting a redistribution of local solvent exposure rather than global structural unfolding (Fig. S3D). Collectively, these results indicated that the docking-predicted minor-groove binding conformation of **9b** remains dynamically stable over the simulated time scale and can be maintained under physiological-like conditions.

However, even if **9b** binds to bacterial DNA through the minor groove, it may not significantly affect the DNA function, such as Hoechst. Therefore, the direct mechanism of **9b** on bacterial DNA was first explored through agarose gel electrophoresis. Plasmid pUC19 DNA (150 ng/ml) incubated with **9b** (1 to 100 μg/ml) for 6 h showed progressive band fading, with complete degradation at 50 μg/ml (Fig. 6F). Time-course analysis confirmed full degradation after 6 h at 75 μg/ml (Fig. 6G), and similar results were observed after purifying DNA–drug complexes (Fig. 6H). Furthermore, genomic DNA extracted from MRSA treated with **9b** (0.5 to 4 μg/ml) for 3 h displayed dose-dependent degradation, with complete disappearance at concentrations ≥1 μg/ml (Fig. 6I). Collectively, these results demonstrated that compound **9b** exhibits a good cleavage effect on MRSA’s DNA. To further confirm the DNA damage in bacterial cells, we detected changes in the levels of 8-OHdG, a well-established biomarker of oxidative DNA damage produced by hydroxyl radical attack on guanine residues [42]. The levels of 8-OHdG increased significantly in a concentration-dependent manner following **9b** treatment (Fig. 6J). In parallel, the total protein content of MRSA, including intracellular and extracellular fractions, declined markedly after **9b** treatment in a concentration-dependent fashion (Fig. 6K). To distinguish DNA-driven inhibition of biosynthesis from protein loss due to membrane leakage, 32 μg/ml of melittin was severed as a membrane-disrupting control lacking DNA-binding activity. After melittin exposure, no significant change in MRSA total protein was detected. These data indicated that the reduction observed after **9b** treatment was attributable primarily to DNA-damage-mediated suppression of transcription and translation rather than membrane rupture.

However, DNA damage alone typically requires time to exert a bactericidal effect, whereas compound **9b** had already demonstrated rapid, concentration-dependent killing in previous time-kill studies. Notably, many amphiphilic cationic compounds structurally similar to **9b** have been reported to interact with negatively charged phospholipids, leading to membrane disruption. Therefore, subsequent experiments were conducted to further determine whether **9b** compromises the integrity of bacterial membranes, thereby supporting its proposed dual-target antibacterial mechanism.

#### Effects of compound 9b on bacterial cell membranes

**Fig. 7.**
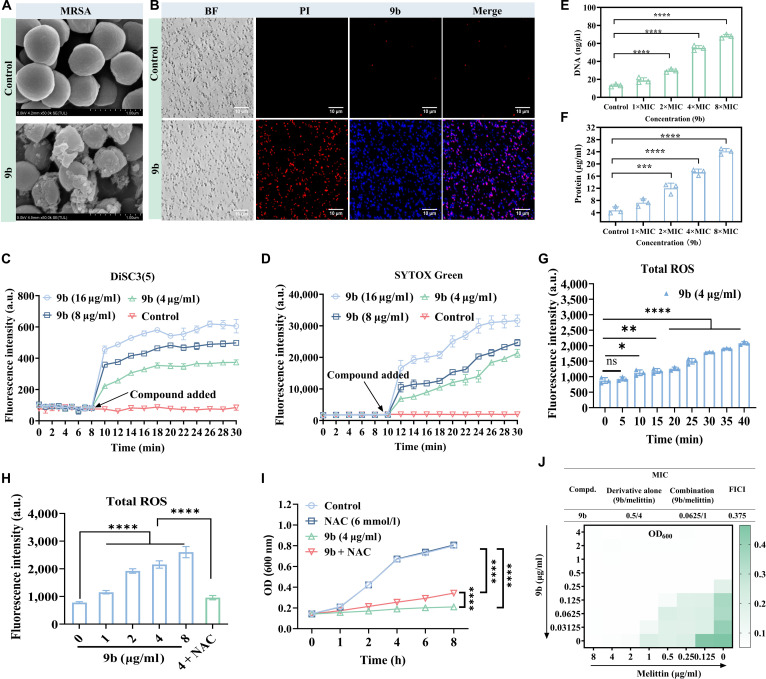
Effects of compound 9b on bacterial cell membranes. (A) Scanning electron microscopy (SEM) images showing the morphology of MRSA-16 cell membranes. Untreated MRSA-16 cells were used as the control group; scale bar: 1.0 μm. Control: no treatment. Scale bar: 50 μm. (B) Fluorescence microscopy images of MRSA-16 cells stained with propidium iodide (PI) following exposure to compound 9b. (C) Cytoplasmic membrane depolarization of 9b using 3,3′-dipropylthiocarbocyanine iodide [DiSC3(5)]. The blank control is bacteria without drug treatment. (D) Membrane permeability of methicillin-resistant *Staphylococcus aureus* (MRSA) was detected using SYTOX Green after treatment with different concentrations of 9b at indicated times. (E) The intracellular reactive oxygen species (ROS) changes following the action of 9b on MRSA-16 were detected using the fluorescent probe dichlorodihydrofluorescein diacetate (DCFH-DA). (F) Protein leakage caused by the action of compound 9b on MRSA-16. (G) Time-dependent accumulation of total ROS in MRSA-16 induced by compound 9b. (H) Total intracellular ROS levels in MRSA-16 after treatment with compound 9b at different concentrations. The ROS scavenger NAC was used as a control. (I) Growth curves of MRSA-16 treated with 9b (4 μg/ml) in the presence or absence of N-acetyl-L-cysteine (NAC) (6 mmol/l). (J) Synergistic antibacterial activity of 9b combined with melittin against MRSA-16 determined by the checkerboard assay. The calculated fractional inhibitory concentration index (FICI) value indicates synergy. (E) to (I) are presented as means ± SD (*n* = 3 biological replicates). Statistical significance was determined by 1-way analysis of variance (ANOVA) with Tukey’s multiple-comparison test: *P < 0.05, **P < 0.01, ***P < 0.001, ****P < 0.0001. a.u., arbitrary units; ns, not significant.

It is well known that the biophysical integrity and function of the bacterial membrane are critical for the growth and survival of bacteria [22,43–47]. Previously, some pioneering studies indicated that many quaternary ammonium compounds have excellent membrane disruption effects [37]. To reveal whether compound **9b** can inherit this property to MRSA, we first visualized the changes in morphological characteristics of **9b**-treated MRSA using scanning electron microscopy and found that the **9b**-treated MRSA had fragmented and wrinkled surfaces (Fig. 7A). Since compound **9b** itself possesses blue fluorescence and can interact with bacterial DNA to emit stronger blue fluorescence, we used it and another fluorescent probe, propidium iodide (PI), to further investigate its disruptive effects on bacterial cell membranes. For PI, only if the cell membrane was damaged it can pass through and bind to intracellular DNA to emit red fluorescence. The fluorescence microscopy images clearly indicated that compound **9b** successfully disrupts the bacterial cell membranes and interacts with bacterial DNA to emit blue fluorescence (Fig. 7B), with similar fluorescent properties as the fluorescent probe 4′,6-diamidino-2-phenylindole. This property of **9b** could be well used for cell staining and imaging. Subsequently, the membrane depolarization assay was performed by using 3,3′-dipropylthiocarbocyanine iodide [DiSC3(5)] fluorescent probe, which can aggregate within cell membrane, thereby resulting in fluorescence quenching, to further demonstrate the disruption effect of compound **9b** to bacterial cell membranes. A large increase in fluorescence intensity was observed upon treatment of MRSA with **9b** (Fig. 7C), suggesting that membrane disruption contributes to its bactericidal effect. Quantitative analysis using SYTO 9 revealed that **9b** rapidly enhanced bacterial membrane permeability in a dose- and time-dependent manner within 30 min (Fig. 7D). Their temporal correlation analysis with Fig. 7G indicated that membrane damage occurred prior to significant elevation of reactive oxygen species (ROS), suggesting that the accumulation of ROS is a downstream event rather than the initiating trigger. Taken together, the above scanning electron microscopy, membrane depolarization, and fluorescence microscopy experiments fully demonstrated that **9b** can effectively disrupt bacterial cell membranes, ultimately leading to cell death.

#### ROS and leakage of DNA and protein measurement

It is known that rupture of bacterial cell membranes or changes in permeability can induce intracellular ROS generation and the release of cellular components, including DNA and proteins, accelerating bacterial death [48,49]. Therefore, we first quantified the leakage DNA and protein levels to confirm the disruption of bacterial cell membrane and found that their release increased in a concentration-dependent manner upon treatment with **9b** (Fig. 7E and F), with the total amount of ROS also increased accordingly (Fig. 7H), illustrating that when **9b** interacted with MRSA, the level of ROS concentration in MRSA increased significantly with increasing concentrations of **9b**. Upon reaching a concentration of 32 μg/ml, the fluorescence intensity of **9b**-treated group was 4 times more than that of the blank control, suggesting that **9b** facilitates ROS production following the disruption of bacterial cell membranes. To clarify the relationship among membrane disruption, ROS accumulation, and DNA damage during **9b**-mediated killing, we employed the ROS scavenger N-acetyl-L-cysteine (NAC) to suppress oxidative stress and assess the contribution of ROS. As shown in Fig. 7G, NAC markedly reduced the ROS signal induced by **9b** to a level close to that of untreated bacteria at the same **9b** dose. Time-course analyses of membrane depolarization and membrane permeabilization (Fig. 7C and D) indicated that membrane damage occurred earlier, whereas ROS elevation followed thereafter, supporting ROS as a downstream event of membrane disruption. We then compared the antibacterial activity of **9b** in the presence or absence of NAC (Fig. 7I) and observed only a modest reduction upon ROS suppression. Moreover, ROS scavenging significantly attenuated **9b**-induced oxidative DNA damage (Fig. S4), demonstrating that ROS contributes to DNA oxidation but is not the sole determinant of DNA damage or bacterial death. To further explore nonmembrane-mediated antibacterial properties, melittin, a membrane-disruptive peptide, was used for combination testing (Fig. 7J). The calculated fractional inhibitory concentration index was 0.375, indicating synergy. This observation is consistent with an additional mechanism beyond membrane disruption and supports the contribution of DNA targeting/binding to the overall antibacterial activity of **9b**. Collectively, the findings indicated that compound **9b** exhibited strong membrane-targeting activity, leading to disruption of bacterial cell membranes, triggering intracellular ROS accumulation and membrane disruption, which leads to the release of intracellular DNA/proteins and rapid bacterial death.

#### Transcriptome analysis of MRSA treated with 9b

**Fig. 8.**
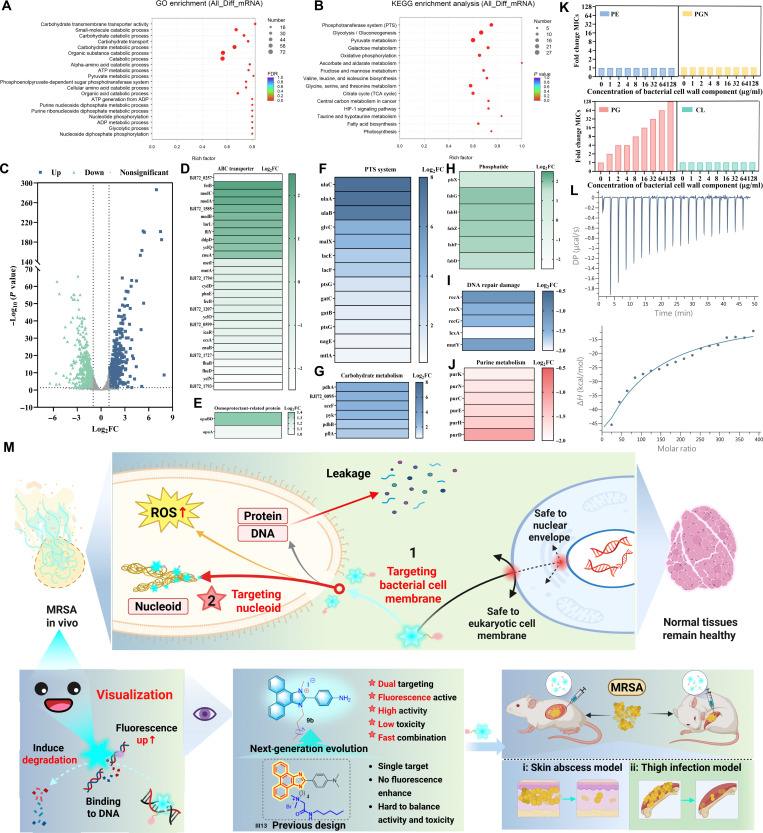
Antibacterial mechanism of compound 9b against the clinical MRSA-16 isolate. (A) Gene Ontology (GO) enrichment analysis. (B) Kyoto Encyclopedia of Genes and Genomes (KEGG) pathway analysis. (C) Volcano plot showing transcriptomic changes in MRSA-16 after exposure to compound 9b. (D to J) Differentially expressed genes (DEGs) of methicillin-resistant *Staphylococcus aureus* (MRSA) N315 altered by 9b treatment. (K) Assessment of the impact of exogenous peptidoglycan (PGN), phosphatidylglycerol (PG), phosphatidylethanolamine (PE), and cardiolipin (CL) (0 to 64 μg/ml) on the anti-MRSA activity of compound 9b. (L) Isothermal titration calorimetry (ITC) analysis of the interaction of PG with 9b. (M) The possible dual mechanism of phenanthro[9,10-d]imidazole molecule 9b against MRSA involves disruption of membrane integrity and induction of DNA degradation. FDR, false discovery rate; ADP, adenosine diphosphate; ATP, adenosine triphosphate; TCA, tricarboxylic acid; HIF-1, hypoxia-inducible factor 1; DP, differential power.

To further explore the molecular mechanism of **9b** against MRSA and the changes in gene expression induced at the gene level, we performed transcriptome analysis in the **9b**-treated group and the blank group using RNA sequencing. Gene Ontology and Kyoto Encyclopedia of Genes and Genomes (Fig. 8A and B) enrichment indicated significant enrichment in pathways associated with carbohydrate metabolism, phosphotransferase systems, and membrane transport, as well as purine metabolism and DNA repair processes. The expression levels of differentially expressed genes (DEGs) between the administration group and the blank group were assessed by transcriptome sequencing, and 986 DEGs were screened out according to the screening conditions (*P*-adjust ≤ 0.05, |log2FC| ≥ 1). As a result, 484 genes were significantly up-regulated and 502 genes were significantly down-regulated in these DEGs, which is more visualized in the scatterplot of the up-regulation and down-regulation from the volcano. The distribution of up- and down-regulated genes was visualized in the scatter plot (Fig. 8C). As shown in Fig. 8D to J, on the one hand, the osmoprotection-related glycine/betaine transporter protein genes (*opu*A, *opu*BD) were up-regulated, reflecting that the cell membrane permeability of MRSA was obviously increased after treatment with **9b**, which disrupted the function of the bacterial cell membrane and led to the inhibition of the growth of MRSA. The differential up-regulation of ABC transporter protein genes indicated that after treatment with **9b**, the exocytosis function of the drug into the cell was increased, which accelerated the pumping out of the drug and nutrients, leading to the leakage of intracellular macromolecules, and accelerated the death of MRSA. On the other hand, the up-regulation of membrane phosphoester gene reflected that MRSA could improve the function of cell membrane and increase the fluidity of cell membrane by promoting the formation of phospholipid bilayer, thus promoting the repair of cell membrane. These results suggest that the effects of **9b** on MRSA membrane damage are mainly influenced by membrane-associated proteins and membrane phospholipids. Additionally, membrane homeostasis collapse triggers metabolic imbalance, leading to respiratory chain disruption and ROS accumulation. However, the antioxidant defense system, including sodA and katG, was not effectively activated, exacerbating oxidative stress within bacterial cells.

Moreover, experiments including exogenous DNA binding assays and 8-hydroxy-2′-deoxyguanosine (8-OH-dG) quantification demonstrated that compound **9b** specifically binds to the DNA minor groove, directly inducing structural DNA damage and thereby accelerating bacterial death. Purine metabolism is one of the important metabolic pathways, which is associated with the synthesis of genetic material. As displayed in Fig. 8J, gene down-regulation indicates that purine metabolism is inhibited and nucleic acid synthesis is disrupted, resulting in bacterial death. Compound **9b** may also block the DNA repair pathway by inhibiting the repair and homologous recombination-related proteins, RecA, RecX, RecG, lexA, mutY, which destabilizes the bacterial genome and leads to bacterial damage or death. This result suggests that **9b** can intercalate into the DNA minor groove and inhibit purine metabolism, leading to impaired nucleic acid synthesis, and interfere with DNA repair pathways, ultimately resulting in bacterial damage. In addition, the bacterial death caused by compound **9b** is also related to carbohydrate metabolism and the phosphotransferase system (Fig. 8F). By increasing the uptake of nutrients from the environment, the bacterial body is able to obtain more energy to repair the damage caused by the compounds to the bacterial cell membranes, and a large accumulation of pyruvate, thereby disrupting bacterial cellular metabolism and resulting in cell death.

#### Study on the membrane-targeting mechanism of 9b

To further elucidate the membrane-targeting mechanism of **9b**, we evaluated its anti-MRSA activity in the presence of exogenously added bacterial cell wall component peptidoglycan (PGN) and membrane phospholipids, including phosphatidylglycerol (PG), phosphatidylethanolamine (PE), and cardiolipin (CL). From Fig. 8K, increasing concentrations of PG gradually reduced the anti-MRSA activity of **9b**. When exogenous PG reached 128 μg/ml, the MIC of **9b** against MRSA increased from 1 to 128 μg/ml. In contrast, the MIC values of **9b** did not change (MIC = 1 μg/ml) when exogenously added with different concentrations of PE, CL, and PGN (Fig. 8K). Meanwhile, the results of isothermal titration calorimetry (ITC) further demonstrated that **9b** has good affinity for PG, with a dissociation constant (K_D_, M^−1^) value of 2.99×10^6^ (Fig. 8L), which is far more superior to recently reported LTX-315 [50], comparable to A20 [51]. We further employed MD simulations to preliminarily explore the potential binding mode between **9b** and PG (Fig. S5A to D). Following initial conformational relaxation, the **9b**–PG complex entered a stable dynamic equilibrium. Both the RMSD and radius of gyration fluctuated within a limited range throughout the simulation, with no evidence of persistent dissociation or global unfolding, indicating that the complex remains structurally stable while retaining a certain degree of conformational flexibility. Meanwhile, the solvent-accessible surface area remained relatively stable over time, further supporting that the complex exists in a dynamically stable state in solution, with its stability more likely arising from the cooperative contribution of hydrophobic and van der Waals interactions. In a representative binding conformation (Fig. S5E), the amino group on benzene of **9b** forms a well-defined hydrogen bond with a phosphate oxygen atom in the PG headgroup region, with a distance of approximately 2.1 Å, indicating favorable geometric alignment and directionality. This hydrogen bond anchors the spatial orientation of **9b** within the binding site, directing its aromatic scaffold toward the hydrophobic acyl chain region of the lipid and thereby facilitating hydrophobic contacts and improving conformational complementarity. This was also supported by molecular mechanics–generalized born surface area analysis (Table S1). The interaction between **9b** and PG is thermodynamically favorable, with a calculated binding free energy (*Δ*G_bind) of −21.97 ± 2.42 kcal/mol. Energy decomposition further revealed that the binding is predominantly stabilized by van der Waals and hydrophobic contributions, whereas electrostatic interactions are largely compensated by solvation effects. Together, these results suggested that hydrogen bonding at the polar headgroup region mainly governs binding-site localization and orientation, while the overall stability of the **9b**–PG complex is maintained through the cooperative action of multiple noncovalent interactions. Collectively, these results implied that **9b** exerts its membrane-disrupting effect through specific binding to PG, thereby contributing to its selectivity toward bacterial cells.

Integrating these findings with earlier observations, a dual-action antibacterial mechanism of **9b** against MRSA was proposed (Fig. 8M). First, **9b** selectively associated with PG within bacterial membranes, leading to membrane integrity disruption, enhanced permeability, and depolarization. As a result, intracellular ROS levels increased and cellular contents such as DNA and proteins leaked out, leading to accelerated bacterial death. Second, **9b** directly bound to the minor groove of DNA, inducing DNA damage and degradation, while simultaneously interfering with purine metabolism and DNA repair pathways, as revealed by transcriptomic analysis. Together, these actions collapsed membrane homeostasis and genomic stability, culminating in rapid and potent bactericidal effects. Moreover, **9b** exhibited fluorescence enhancement upon bacterial interaction, which enabled real-time visualization of its antimicrobial process.

Given its robust antibacterial efficacy and unique dual-targeting mechanism, it was essential to ensure that **9b** possessed a favorable safety profile for potential clinical application. Therefore, the subsequent section focused on assessing the biosafety of **9b** in mammalian systems.

### Membrane selectivity, pharmacokinetics, plasma stability, and toxicity studies

The above mechanistic findings demonstrated that compound **9b** specifically targeted the phospholipid component PG in bacterial membranes and subsequently interacted with bacterial DNA, exerting potent antibacterial activity through a dual-action mechanism. Given this mode of action, ensuring favorable in vitro and in vivo safety and plasma stability is critical for its therapeutic potential.

**Fig. 9.**
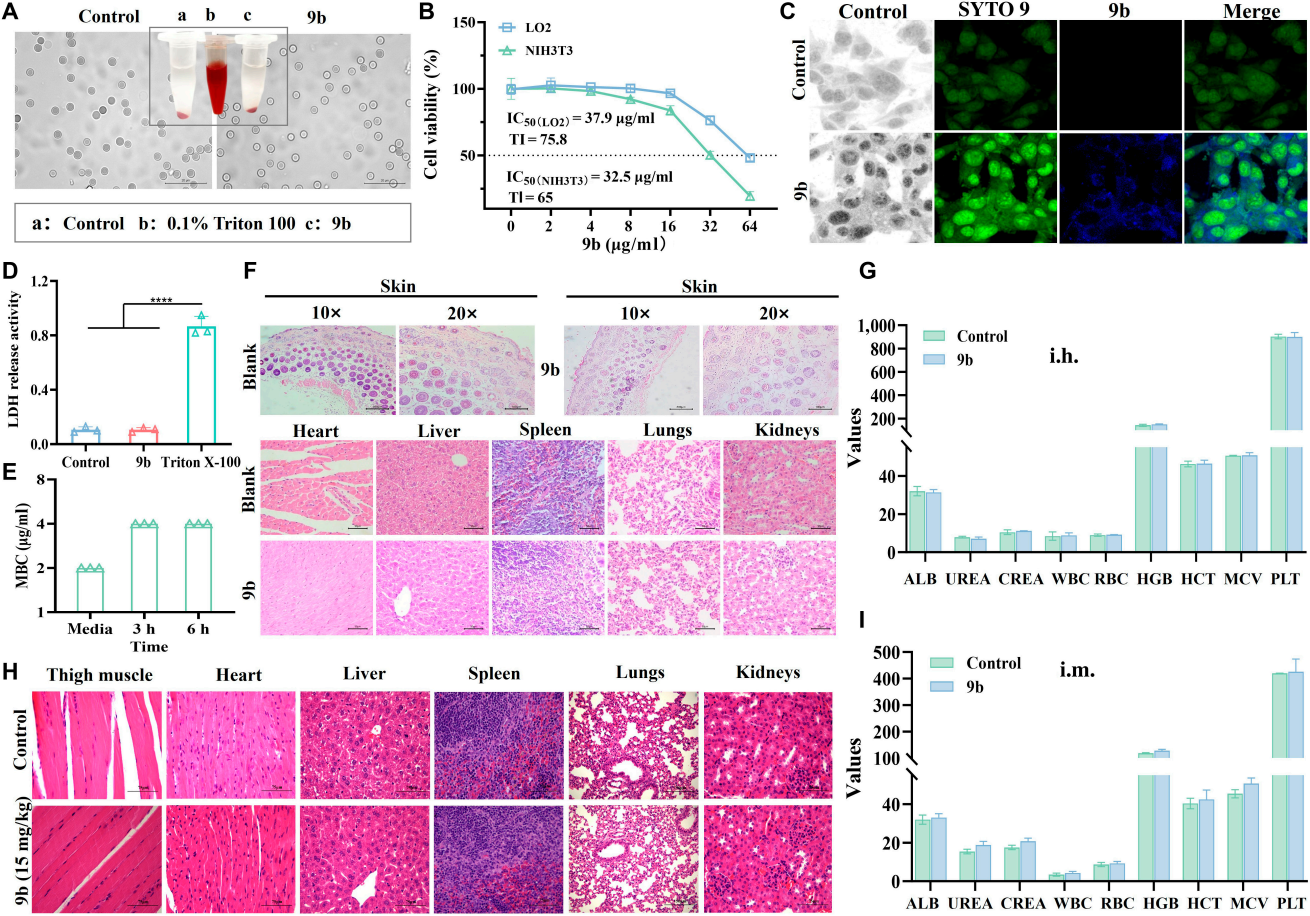
(A) Microscopic images of sheep blood erythrocytes after treatment with 9b. Scale bar: 50 μm. (B) Cytotoxicity of compound 9b against LO2 and NIH3T3 cells. Data are presented as means ± SD (*n* = 3 biological replicates). (C) Fluorescence microscopy images of LO2 cells after treatment with 9b (16 μg/ml) for 24 h and staining with SYTO 9. Control: Nontreated cells. Bright field. Scale bar: 200 μm. (D) Lactate dehydrogenase (LDH) release activity testing on LO2 cells with 9b (16 μg/ml) for 24 h. Data are presented as means ± SD (*n* = 3 biological replicates). Data are presented as means ± SD (*n* = 3 biological replicates). Statistical significance was determined by 1-way analysis of variance (ANOVA): ****P < 0.0001. (E) Plasma stability of compound 9b against MRSA-16. (F to H) In vivo safety evaluation of compound 9b by hematoxylin and eosin (H&E) staining treated with subcutaneous (i.h.) and intramuscular (i.m.) injections on mice, respectively. Scale bar: 50, 75, or 150 μm. Routine blood testing and blood biochemical indexes in mice treated with i.h. (G) and i.m. (I) compound 9b. ALB, albumin; UREA, urea; CREA, creatinine; WBC, white blood cell; RBC, red blood cell; PLT, platelet count; MCV, mean corpuscular volume; HGB, hemoglobin; HCT, hematocrit. Data are presented as means ± SD (*n* = 6 biological replicates).

To provide exposure context for the in vivo efficacy evaluation, we determined the pharmacokinetic properties of **9b** in rats following intravenous administration (Table 3). After intravenous dosing (2 mg/kg), **9b** reached a maximum plasma concentration (Cmax) of 2,173.33 ng/ml within 5 min and exhibited a terminal half-life (*t*₁/₂) of 5.81 h, with an area under the plasma concentration–time curve from time zero to infinity (AUC₀–inf) of 1,273.87 h·ng/ml. The calculated volume of distribution (Vdss) of 3.03 l/kg suggested that **9b** is not confined to the plasma compartment and undergoes appreciable tissue distribution. These results provided important supportive information by confirming that **9b** remains chemically detectable in vivo and possesses the capacity for tissue distribution. To further evaluate the blood compatibility of **9b**, the morphology of sheep red blood cells was examined under microscopy after incubation with the compound. Even at a high concentration of 128 × MIC against MRSA-16, red blood cells treated with **9b** showed no observable morphological changes compared with the blank control, maintaining intact membranes without signs of hemolysis. In contrast, the positive control (0.1% Triton X-100) caused complete erythrocyte lysis (Fig. 9A). Quantitative hemolysis analysis was conducted to generate dose–response curves over concentrations encompassing clinically relevant exposure ranges centered on the MIC (Fig. S6). The data showed that **9b** caused minimal hemolysis within this exposure window, supporting its acceptable hemocompatibility under clinically relevant conditions.

**Table 3.** Pharmacokinetic parameters of 9b in rats

Route	Dose	Cmax (ng/ml)	*T*_max_ (h)	*t*_1/2_ (h)	AUC_0_–t (h·ng/ml)	AUC_0_–inf (h·ng/ml)	CL (ml/min/kg)	Vdss (l/kg)
Intravenous	2 mg/kg	2,173.33	0.083	5.81	1,258.01	1,273.87	26.22	3.03

CL, cardiolipin; Cmax, maximum plasma concentration; Vdss, volume of distribution; AUC_0_-t, area under the plasma concentration–time curve from time zero to the last measurable time point; AUC_0_-inf, area under the plasma concentration–time curve from time zero to infinity

Cytotoxicity assessment revealed that LO2 and NIH3T3 cells maintained >90% viability at 64 μg/ml of **9b**, with half maximal inhibitory concentration (IC_50_) values of 37.9 μg/ml and 32.5 μg/ml, respectively, and high therapeutic index (>65) (Fig. 9B). Confocal fluorescence microscopy further indicated that **9b** entered the cytoplasm but showed no nuclear localization, as only SYTO 9-stained nuclei displayed strong green fluorescence, while **9b** exhibited weak blue fluorescence confined to the cytoplasmic region (Fig. 9C). Importantly, this intracellular distribution did not compromise membrane integrity, as confirmed by lactate dehydrogenase release assays, where lactate dehydrogenase activity in **9b**-treated cells remained comparable to that of the blank control and obviously lower than the Triton X-100-positive control (Fig. 9D). These findings collectively demonstrated that **9b** selectively targeted bacterial cells while preserving mammalian cell membrane integrity and avoiding nuclear DNA interaction. Due to the presence of various hydrolytic enzymes in plasma, such as cholinesterases, lipases, and phosphatases, many compounds undergo rapid enzymatic hydrolysis, leading to a sharp decline in their plasma concentration and consequently a loss of antimicrobial efficacy [52–54]. As illustrated in Fig. 9E, the minimum bactericidal concentration (MBC) of **9b** remained unchanged after treatment with plasma for 6 h, suggesting that **9b** is not easily hydrolyzed by hydrolases and possessed commendable plasma stability. Furthermore, as shown in Fig. S7, the chemical stability of **9b** in rat plasma was evaluated by monitoring the percentage of intact compound over time. The results showed that **9b** remained largely intact in plasma, with approximately 92% of the compound remaining after 4 h. Kinetic fitting using a first-order degradation model yielded an apparent degradation rate constant (ke) of 0.0173 h^−1^, corresponding to an estimated half-life (*t*₁/₂) of 40.17 h. In contrast, the reference compound propantheline showed substantially faster degradation under the same conditions, with only 48.5% remaining after 4 h and a calculated half-life of 3.85 h. These results indicated that **9b** possesses favorable chemical stability in plasma compared with the reference compound. To further clarify the behavior of **9b** in plasma, its plasma protein binding was determined in rat plasma. At a concentration of 4 μg/ml, **9b** exhibited a free fraction (Fu) of 2.47% and a corresponding protein-bound fraction (Fb) of 97.53%, indicating substantial plasma protein binding. These results suggested that a large proportion of **9b** exists in a protein-bound form in plasma. Although **9b** exhibited a relatively high plasma protein binding rate, measurable antibacterial activity was still observed in plasma-containing systems (Fig. 9E). This phenomenon may arise from several factors. First, plasma protein binding is a reversible equilibrium, allowing bound drug to continuously release free compound as the free fraction is consumed. Second, **9b** appeared to have a strong affinity for bacterial membrane components such as PG, which may enable the compound to partition preferentially toward bacterial cells rather than remain protein-bound. Finally, even a small free fraction may remain pharmacologically active, particularly for membrane-active antibacterial agents. These findings demonstrated that **9b** exhibited favorable safety in eukaryotic cells and can selectively compromise bacterial cell membranes and interact with bacterial DNA at therapeutic concentrations, thereby displaying its antibacterial activity.

Subcutaneous administration of compound **9b** at various doses (40, 20, 10, 5, and 2.5 mg/kg) was performed on healthy mice to monitor general condition and survival over a 24-h period for preliminary in vivo toxicity assessment. Animals in all treatment groups remained in good health, and no visible abnormalities were detected relative to the blank control group. The survival rate was 100% for all tested doses (Table S2). Mice treated with 40 or 20 mg/kg exhibited mild redness and swelling at the injection site, whereas those receiving 10, 5, or 2.5 mg/kg showed no obvious skin irritation, and their appearance was comparable to that of the untreated controls. These observations suggest that compound **9b** demonstrates acceptable in vivo tolerability at doses ≤10 mg/kg. Next, the blood and skin at the administration site of the mice receiving the maximum dose (10 mg/kg), as well as tissues of vital organs for blood biochemistry indexes and hematoxylin and eosin staining tests, respectively. Additionally, Fig. 9F showed no significant abnormalities in the tissue sections (skin, heart, liver, spleen, lungs, and kidneys) of mice in **9b** group compared with those of the blank group. Figure 9G displays that the blood biochemistry and blood routine indexes of the mice treated with **9b** (10 mg/kg) were within the normal range and almost the same as that of the blank control group.

Additionally, we investigated the survival status of mice following intramuscular injections of different concentrations of **9b** (20, 15, 10, and 5 mg/kg) into the thigh muscles. The results showed that the mice in all groups survived (Table S3), but the mice in **9b** (20 mg/kg) had swollen and hardened thigh muscles. The thigh muscles of the remaining groups of mice were elastic and did not differ from the control group, indicating that the injection dose without adverse effects should be ≤15 mg/kg. Mice with leg muscles injected with 15 mg/kg of **9b** solution showed no pathological alterations in the major organs of mice (heart, liver, spleen, lungs, and kidneys), no inflammatory cells were observed in the thigh muscles, and the muscle fibers did not show any breakage (Fig. 9H). There was no significant difference in blood routine and blood biochemistry indexes compared with the control group (Fig. 9I).

Collectively, these results confirmed that **9b** achieved a favorable safety profile both in vitro and in vivo. This was largely attributed to its mechanism-driven dual selectivity—preferential binding to bacterial membranes followed by DNA interaction—while sparing mammalian membranes and nuclear DNA, highlighting its promise as a safe and effective lead compound for MRSA treatment.

### In vivo antibacterial ability

**Fig. 10.**
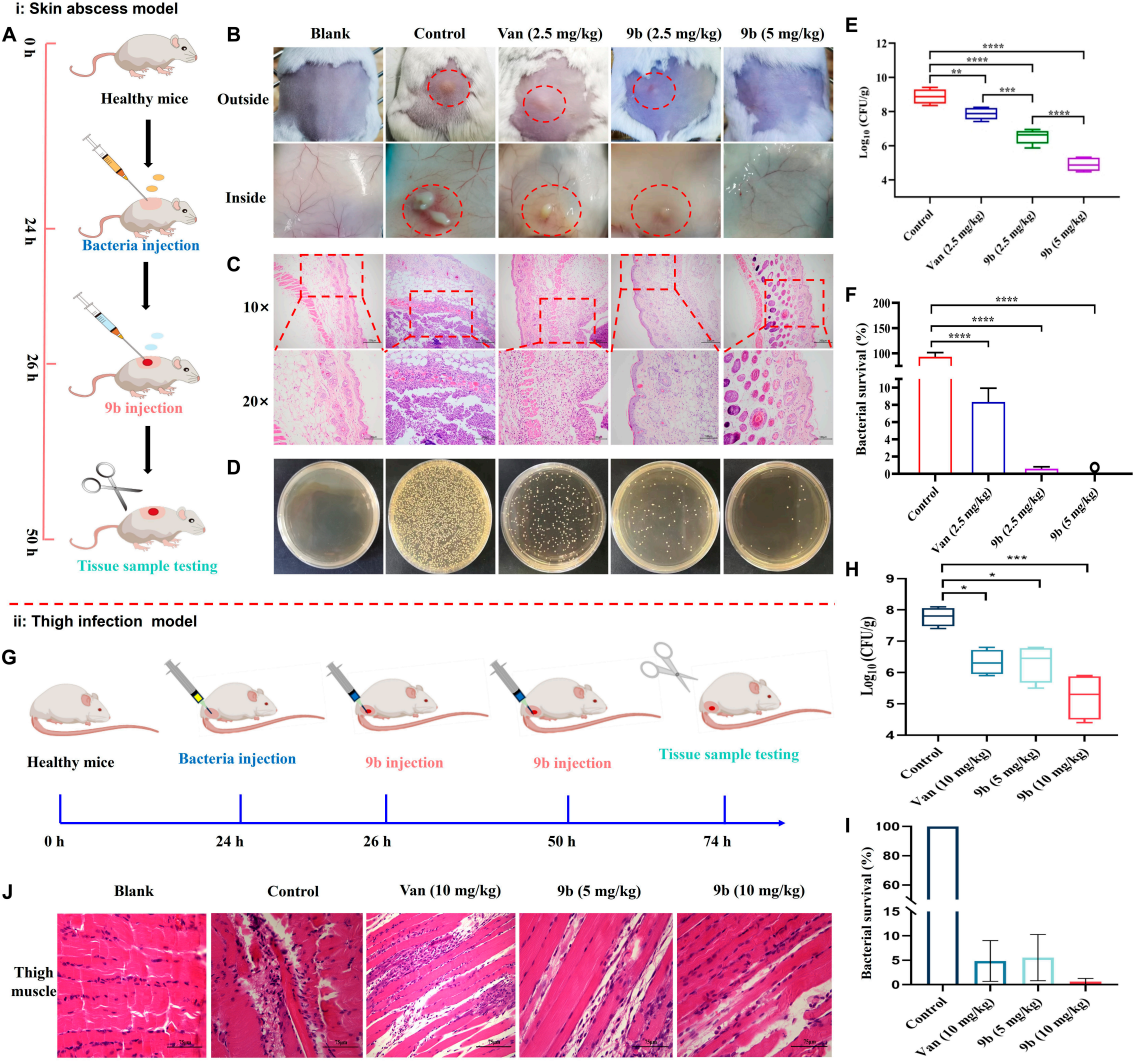
In vivo antibacterial assay. (A) Schematic illustration of the experimental design for the murine methicillin-resistant *Staphylococcus aureus* (MRSA) skin abscess model. (B) Representative images of MRSA-infected skin lesions following different therapeutic interventions. (C) Histopathological sections of infected skin tissues subjected to various treatments; scale bars represent 100 or 200 μm. (D) Representative photographs of MRSA colonies obtained from homogenized infected skin samples after serial dilution and plating. (E) Quantification of bacterial survival in the skin of MRSA-infected mice treated with compound 9b (2.5 and 5 mg/kg) or vancomycin (Van, 5 mg/kg) for 24 h. (F) Bacterial survival in the skin of MRSA-infected mice after administration of 9b (2.5 mg/kg), 9b (5 mg/kg), and Van (2.5 mg/kg) for 24 h. (G) Schematic overview of the experimental procedure for the murine MRSA thigh infection model. (H) Bacterial load in the thigh of MRSA-infected mice of each group treated with 9b (5 and 10 mg/kg) and vancomycin (Van) (5 mg/kg) for 48 h. (I) Bacterial survival in the thigh of MRSA-infected mice of each group treated with 9b (5 and 10 mg/kg) and vancomycin (Van) (10 mg/kg) for 48 h. (J) Representative hematoxylin and eosin (H&E)-stained sections from thigh muscle after various treatments. Scale bar: 75 μm. The data are presented as the means ± SE from 6 independent experiments. Statistical significance was determined by 1-way analysis of variance (ANOVA) with Tukey’s multiple-comparison test: *P < 0.05, **P < 0.01, ***P < 0.001, ****P < 0.0001.

Considering its pronounced in vitro anti-MRSA activity and favorable safety profile, the in vivo antibacterial performance of compound **9b** was further investigated using a murine MRSA skin abscess model established according to previously reported protocols (Fig. 10A). A 6× 10^8^ CFU/ml dose of MRSA-16 was injected subcutaneously into the dorsal skin of mice, followed by the administration of **9b** (2.5 and 5 mg/kg) and vancomycin (2.5 mg/kg) for treatment 2 h postinfection. As displayed in Fig. 10B, extensive abscess formation was evident in the dorsal skin and subcutaneous layers of mice in the control group. It is noteworthy that no abscesses were observed in mice treated with a high dose of **9b** (5 mg/kg), and the dorsal skin and subcutaneous tissues were indistinguishable from those of the blank group. By comparison, animals treated with vancomycin (2.5 mg/kg) or low-dose **9b** (2.5 mg/kg) still exhibited visible abscesses and localized erythema in the subcutaneous tissue. Additionally, histopathological analysis of the infected skin revealed extensive infiltration of inflammatory cells in the subcutaneous tissue, with further spread into the dermis and epidermis, indicative of a septic inflammatory response (Fig. 10C). In contrast, skin tissues from mice treated with vancomycin or low-dose **9b** (2.5 mg/kg) exhibited only mild infiltration of inflammatory cells, indicating moderate improvement of infection (Fig. 10C). Notably, high-dose **9b** (5 mg/kg) treatment resulted in skin morphology nearly indistinguishable from that of the blank group (Fig. 10C).

Moreover, to further investigate the in vivo efficacy of **9b**, bacterial load and survival rates in the infected skin tissues of mice were determined by plate counting. We quantified the bacterial load in infected skin tissues of mice. As shown in Fig. 10D, only a few bacteria were observed in the **9b**-treated groups (2.5 and 5 mg/kg) compared with the model group, and at same dosage, **9b** exhibited superior antibacterial activity in vivo compare to vancomycin. Quantitative analysis further demonstrated that the bacterial load in the infected skin of the control group was approximately 8.9 log₁₀ CFU/g (Fig. 10E). Treatment with **9b** (2.5 mg/kg) and vancomycin (2.5 mg/kg) reduced bacterial load by 2.4 and 1.0 log₁₀ CFU/g (Fig. 10E), respectively, corresponding to decreases in bacterial survival rates of 92.6% and 84.9% (Fig. 10F). Notably, administration of **9b** at 5 mg/kg produced a more pronounced effect, with the bacterial load reduced by 4.0 log₁₀ CFU/g and bacterial survival nearly abolished (Fig. 10E).

Furthermore, systemic pathological changes were evident in the model group, with the liver, lung, and kidney exhibiting signs of congestion, interstitial edema, alveolar wall thickening, inflammatory infiltration, and renal tubular epithelial cell swelling (Fig. S8A). Conversely, the mice in the other treatment groups had no aberrant alterations in major organs. The mice in vancomycin (2.5 mg/kg) group exhibited good improvement in pathological symptoms compared with the mice in the control group, especially the mice in the **9b** (2.5 and 5 mg/kg) groups basically tended to be normalized with no obvious pathological abnormalities (Fig. S8A). Collectively, **9b** with outstanding in vivo therapeutic efficacy has the potential to be a candidate for combating MRSA infections.

To further investigate the in vivo efficacy of **9b**, a mouse thigh infection model was developed using a clinical MRSA-16 isolate (Fig. 10G). From Fig. 10H, the bacterial load in the thigh tissues of mice in the control group was approximately 7.8 log_10_ CFU/g. In contrast, infected mice treated with low-dose **9b** (5 mg/kg) and vancomycin (10 mg/kg) both achieved a reduction in bacterial load of about 1.5 log_10_ CFU/g, resulting in bacterial survival rates of approximately 94.45% and 95.14%, respectively, indicating significant antibacterial efficacy. Notably, infected mice treated with **9b** (10 mg/kg) exhibited a reduction in thigh bacterial load of approximately 2.6 log_10_ CFU/g and a reduction in bacterial survival of about 99.36% (Fig. 10H and I), suggesting that **9b** has strong in vivo anti-MRSA efficacy.

Finally, histopathological evaluation was conducted to assess the systemic toxicity and tissue protective effects of **9b** in a thigh model of mice infected with a clinical MRSA-16 isolate. As shown in Fig. 10J, the control group (MRSA infection without treatment) exhibited extensive muscle fiber fragmentation, dissolution, and dense inflammatory cell infiltration, indicative of severe tissue damage. In contrast, vancomycin (10 mg/kg) treatment partially alleviated these pathological features, with reduced inflammation and partially preserved muscle structure. Notably, **9b** at both 5 and 10 mg/kg effectively preserved the integrity of skeletal muscle fibers and obviously reduced inflammatory cell infiltration, suggesting robust antibacterial efficacy and tissue protection. For major organs (Fig. S8B), MRSA infection induced systemic injury, particularly in the spleen, lungs, and kidneys. The control group showed signs of splenic extramedullary hematopoiesis, pulmonary inflammation, and renal tubular degeneration, reflecting infection-associated systemic stress. Treatment with vancomycin improved these pathological changes to a certain extent. Importantly, mice treated with **9b** displayed well-preserved tissue architecture across all examined organs, with no signs of necrosis or massive inflammatory infiltration, indicating no evident systemic toxicity at either dose. These findings support the therapeutic potential and safety profile of **9b** in treating MRSA infections.

**Fig. 11.**
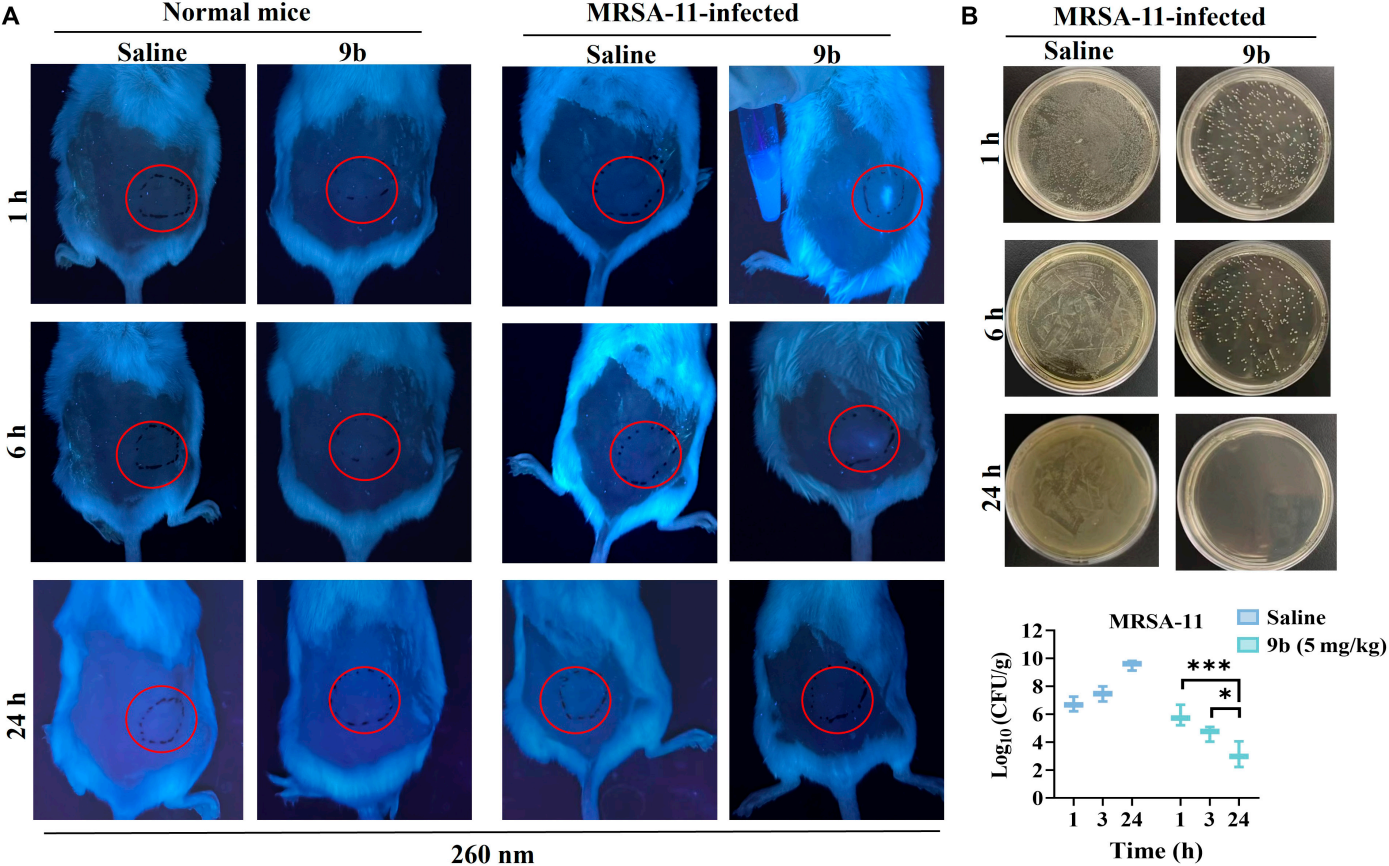
In vivo antibacterial visual monitoring assay. (A) Representative in vivo fluorescence images of normal mice and MRSA-11-infected mice treated with saline or 9b. Imaging was performed at the indicated time points (1, 6, and 24 h) under ultraviolet (UV) excitation (260 nm). Red circles indicate the infection sites. No detectable fluorescence signal was observed in normal mice treated with either saline or 9b, indicating minimal background emission of free 9b in the absence of bacterial binding. In contrast, strong fluorescence signals were detected at the infected sites of 9b-treated mice as early as 1 h postadministration. (B) Representative agar plate images showing bacterial colony formation from infected tissues collected at 1, 6, and 24 h after treatment. Quantitative analysis of bacterial burden (log_10_ CFU/g tissue) is shown below. Data are presented as means ± SD (*n* = 3). Statistical significance was determined by 1-way analysis of variance (ANOVA) with Tukey’s multiple-comparison test: *P < 0.05, ***P < 0.001.

After confirming the favorable in vivo safety and antibacterial efficacy of **9b**, we further evaluated its potential for real-time visualization of infection. Using a mouse skin infection model, imaging was performed at the characteristic excitation wavelength of **9b** (260 nm). As shown in Fig. 11A, no detectable fluorescence signal was observed in normal mice treated with either saline or **9b**, which is consistent with the low quantum yield of free **9b** and indicates minimal background signal in the absence of bacterial binding. In contrast, in MRSA-11-infected mice, a distinct fluorescence signal was observed at the infection site as early as 1 h after **9b** treatment, whereas no comparable signal was detected in saline-treated controls. This finding was consistent with our in vitro results (Fig. 5D), demonstrating that fluorescence intensity increases obviously upon bacterial binding. Moreover, the temporal changes in fluorescence intensity correlated with the reduction in bacterial burden as determined by CFU counting (Fig. 11B), suggesting that the fluorescence signal reflects dynamic changes in infection load. Collectively, these results indicated that **9b** enables infection-site visualization with minimal background interference and exhibits potential as a theranostic agent for antibacterial monitoring.

## Conclusion

In summary, we herein report the design and synthesis of a series of fluorescent phenanthro[9,10-d]imidazole compounds, in which **9b** was screened out with low toxicity, excellent anti-MRSA activity, rapid bactericidal properties, and good plasma stability and not easy in developing drug resistance. Additionally, **9b** displayed good fluorescence properties, with a remarkable increase in fluorescence intensity upon interaction with bacteria, allowing visualization and monitoring of the bactericidal process. Mechanistic studies revealed that **9b** possesses a dual antibacterial mechanism: Firstly, it can specifically bind to PG on bacterial cell membranes to disrupt its membranes, thereby enhancing ROS production and facilitating protein/DNA leakage, which accelerates bacterial death. Simultaneously, **9b** can also bind to bacterial DNA and induce degradation, thus exerting excellent antibacterial activity. Most importantly, **9b** exhibited high biosafety and excellent in vivo therapeutic efficacy, superior to vancomycin at the same dose, both in a mouse skin abscess and a mouse thigh infection models infected with clinically isolated MRSA strains. Currently, some emerging antibacterial therapies, such as nanozymes, have also demonstrated great potential in the integration of antibacterial and diagnostic treatments [55–57]. The key feature is that they can enhance the catalytic production of ROS through specific external stimuli to mediate antibacterial effect, which also endows the antibacterial nanozymes therapy with a more intelligent characteristic. This is also one of the antibacterial mechanisms of the small molecule antibacterial agent **9b** reported in this article. In contrast, **9b** not only shows stronger antibacterial activity but also does not rely on a single antibacterial mechanism. Instead, it has a clear synergistic antibacterial mechanism that targets bacterial cell membranes and DNA. Moreover, small molecules have a more mature foundation in industrial production thus have strong commercial potential. These findings are expected to provide new insights and viable approaches for developing novel anti-MRSA agents with enhanced pharmacological properties.

## Materials and Methods

### Chemicals, bacteria, and instruments

Analytical grades of phenanthrene-9,10-dione, 4-benzenedicarboxaldehyde, methyl iodide, and various amines were acquired from Sigma-Aldrich, Energy-Chemical, and Aladdin Inc. *Staphylococcus aureus* ATCC 29213, *Micrococcus luteu*, *Streptococcus suis*, *Escherichia coli* ATCC 29213, *Klebsiella pneumoniae* 18-227, and *Klebsiella pneumoniae* 18-29 were sourced from the ATCC (American Type Culture Collection). Clinical MRSA isolates were obtained from the first affiliated hospital of Zhengzhou University/University of South China. A Bruker Avance instrument 400 MHz instrument was employed to measure ^1^H/^13^C NMR of phenanthro[9,10-d]imidazole compounds. An Agilent 6545 mass spectrometer was used to determine HRMS of phenanthro[9,10-d]imidazole compounds. The purity of all the title phenanthro[9,10-d]imidazole compounds was larger than 95% by using high-performance liquid chromatography.

### Synthesis of phenanthro[9,10-d]imidazole compounds 5a, 8a, 8b, 9a, and 9b

A solution of phenanthrene-9,10-dione (0.96 mmol) and 4-benzenedicarboxaldehyde (0.96 mmol) was dissolved in glacial acetic acid, followed by the addition of ammonium acetate (19.2 mmol). The reaction mixture was refluxed and agitated at 100 °C for 1 to 2 h. The reaction solution was then cooled to room temperature and poured into cold water, and the solids were recovered by filtration and washed with ice water. Subsequently, the solids were dried in a vacuum to obtain intermediate **3**. Next, to an acetone solution of **3** (0.27 mmol) and 1-bromo *n*-anemonane or 3,3-dimethylallylbromide (1.91 mmol), K_2_CO_3_ (1.91 mmol) was added, and then the reaction was agitated at reflux. Upon completion of the reaction, the solution was concentrated, and the residue was purified by silica gel column chromatography (SG-CC) to provide compounds **6a** and **6b**.

To the ethanol solution of compounds **3** (0.44 mmol) or **6a**/**6b** (0.44 mmol) was added stannous chloride dihydrate (4.40 mmol), then reacted under room temperature. Once the reaction finished, the solvent was concentrated, and EtOAc was added to dissolve the residue followed by adding saturated NaHCO_3_ to adjusted the pH to neutral. Subsequently, the reaction mixture was filtrated and then extracted with EtOAc, followed by drying with anhydrous Na_2_SO_4_, concentration, and isolation to give the compounds **4**, **7a**, and **7b**.

Finally, compound **4** (0.11 mmol) or **7a**/**7b** (0.11 mmol) was dissolved with *N*-methyl-2-pyrrolidone or acetonitrile, followed by the addition of iodomethane (1.10 mmol). After stirring the reaction at 75 °C for 48 h, compounds **5a**, **8a**, **8b**, **9a**, and **9b** were obtained by using SG-CC.

### Synthesis of phenanthro[9,10-d]imidazole compounds 12a, 14a, and 14b

To a solution of phenanthrene-9,10-dione (0.96 mmol) and ethyl 4-formylbenzoate (0.96 mmol) in glacial acetic acid, ammonium acetate (19.2 mmol) was added with stirring at 100 °C for 1 to 2 h. Once the reaction finished, heating was stopped and the reaction was cooled down to room temperature and then poured into iced water, collecting the solids by filtration and washing with iced water. The solid was dried under reduced pressure to give intermediate **11** in 63% yield. Next, compound **11** (0.27 mmol) and 1-bromo *n*-anemonane or 3,3-dimethylallylbromide (1.91 mmol) were dissolved in acetone, after which K_2_CO_3_ (1.91 mmol) was added, and the mixture was refluxed at 50 °C. Upon completion of the reaction, the mixture was extracted with EtOAc, and the combined organic layers were dried over anhydrous Na_2_SO_4_. The solvent was then concentrated, and the mixture was purified to yield compounds **13a** and **13b**. Finally, compounds **11**, **14a**, and **14b** were dissolved by ethylenediamine with stirring at 110 °C and monitored by thin layer chromatography until completion. Then separation was performed by SG-CC to obtain the title compounds **12a**, **14a**, and **14b**. After completion of the reaction, extraction was performed using EtOAc, followed by the amalgamation of the organic phases, drying over anhydrous Na_2_SO_4_, concentration, and subsequent separation to yield compounds **13a** and **13b**.

### Synthesis of phenanthro[9,10-d]imidazole compounds 18a, 20a, and 20b

To a dichloromethane solution of 4-formylbenzoic acid (0.72 mmol), *N*-Boc-ethylenediamine (0.87 mmol), 1-ethyl-3-(3-dimethylaminopropyl)carbodiimide (1.81 mmol), 1-hydroxybenzotriazole (1.08 mmol), and triethylamine (1.81 mmol) were added, then stirring at room temperature under N_2_ for 12 to 14 h. After that, the mixture was washed with saturated saline and extracted with dichloromethane, and then the organic phases were combined, dried with anhydrous Na_2_SO_4_, concentrated, and purified by SG-CC to give the intermediate **16** in a yield of 71%. Subsequently, compound **17** was synthesized in accordance with the approach used for the synthesis of compound **3**. Next, we synthesized intermediate **19a** and **19b** using the same method as for compounds **6a** and **6b**. Finally, intermediates **17**, **19a**, and **19b** (60 mg) were hydrolyzed with HCl/MeOH (4 mol/l) solution. The solvent was removed once reaction finished, and residues were washed by petroleum ether to obtain the title compounds **18a**, **20a**, and **20b**. The structures of all the title compounds **5a**, **8a**, **8b**, **9a**, **9b**, **12a**, **14a**, **14b**, **18a**, **20a**, and **20b** were determined using ^1^H/^13^C NMR and HRMS. High-performance liquid chromatography analysis confirmed a purity of ≥95% for all title compounds. Spectral data of **5a**, **8a**, **8b**, **9a**, **9b**, **12a**, **14a**, **14b**, **18a**, **20a**, and **20b** are presented in the Supplementary Materials.

### Determination of MICs

The MICs of compounds **5a**, **8a**, **8b**, **9a**, **9b**, **12a**, **14a**, **14b**, **18a**, **20a**, and **20b** were tested using microbroth dilution method in compliance with the Clinical and Laboratory Standards Institute guidelines. The detailed procedure follows the previously reported methods [33,58,59]. We repeated the determination of MICs 3 times.

### Physical spectral characterization

The spectral properties of compounds **9b** and **5a** were investigated using a UV–visible spectrophotometer and a fluorescence spectrophotometer. Stock solutions of the compounds were prepared in PBS, 0.9% NaCl solution, and DMSO with a final concentration of 1 × 10^−5^ mol/l. The UV–visible absorption spectra were obtained by scanning the wavelength over an appropriate spectral range. The fluorescence emission spectra were subsequently recorded between 400 and 700 nm. To simulate physiological environmental changes, the pH, ionic strength, and ethanol content of the solution were adjusted, and the corresponding fluorescence spectra of compound **9b** were recorded. The fluorescence quantum yield (Φ) of compound **9b** was determined using quinine sulfate as a standard reference under excitation at 350 nm and calculated according to the relative quantum yield method [60].

### Fluorescence measurement after interaction of 9b with bacteria cells

Single MRSA-16 colonies were cultured in 2 ml Mueller–Hinton broth (MHB) at 37 °C, 200 rpm for 12 h. The culture was then diluted 1:1,000 and further incubated under the same conditions for 3 to 5 h. After incubation, cells were harvested by centrifugation (4,000 rpm, 4 °C, 4 min), washed 3 times with PBS, and resuspended in PBS containing **9b** (32 μg/ml). The suspension was shaken at 37 °C, 180 rpm for 3 h, and samples were collected every 20 min in the dark. Fluorescence intensity of **9b** was determined using a microplate reader with excitation at 260 nm and emission recorded from 300 to 700 nm. Bacterial suspensions without drug treatment served as blank controls, and each condition was tested in triplicate.

### Time-kill kinetic study

The bactericidal activity of compound **9b** against *S. aureus* and MRSA-16 was evaluated using a plate-based colony counting assay [36]. Briefly, an individual bacterial colony was transferred into 1.0 ml of MHB and incubated at 37 °C with shaking at 200 rpm. The resulting culture was subsequently diluted with fresh MHB to achieve a final cell density of 1 × 10^5^ CFU/ml and allowed to grow for an additional 2.5 h. Compound **9b** was then introduced at concentrations corresponding to 4× and 8× the MIC, followed by further incubation at 37 °C and 200 rpm for 8 h. Aliquots were collected at 0, 0.5, 1, 2, 4, 6, and 8 h, and viable bacteria were quantified by colony enumeration. Time-kill curves were constructed by plotting log CFU as a function of incubation time. All experiments were conducted in triplicate.

### Bacterial resistance study

Single colonies of *S. aureus* ATCC 29213 were cultured in MHB medium supplemented with a subinhibitory concentration (1/2 MIC) of **9b** or norfloxacin for 12 h. The resulting cultures were then transferred onto Mueller–Hinton agar plates containing the same subinhibitory concentration and incubated for 24 h. This passage procedure was repeated for 20 consecutive generations, and MICs were evaluated at each generation using the microbroth dilution method.

### Scanning electron microscopy

MRSA-16 in the logarithmic growth phase were coincubated with compound **9b** (final concentration 8× MIC) for 1 h. The mixture was centrifuged (5,000 rpm, 4 °C) and washed with PBS buffer 2 to 3 times, the supernatant was discarded, then 2.5% glutaraldehyde (1.0 ml) was added to fix it, and the mixture was stored at 4 °C overnight. Dehydration was carried out with a gradient concentration of ethanol, and then the bacteria were subjected to critical point drying and gold spraying. Bacterial morphological changes were observed using a high-resolution field emission scanning electron microscope. The same volume of sterile water was added as a blank control group.

### Membrane depolarization study

A monoclonal colony of MRSA was transferred under sterile conditions into sterile MHB medium and cultured for 4 h. After centrifugation, the bacterial pellet was washed 2 to 3 times with PBS and resuspended in an equal volume of PBS containing the fluorescent dye dichlorodihydrofluorescein diacetate (5 μM). The suspension was then incubated in the dark at 37 °C for 30 min. Subsequently, 160 μl of the bacterial suspension was added into a 96-well plate, followed by 40 μl of prediluted compound at different concentrations. After incubation at 37 °C for 45 min, fluorescence intensity was measured at excitation/emission wavelengths of 488/530 nm.

### Fluorescence microscopy study

After incubating MRSA in MHB medium (2.0 ml) at 37 °C for 12 h, **9b** (8× MIC) was added and cultivated under light-avoidance conditions for 4 h. After centrifuging (5,000 rpm, 4 °C, 5 min) and washing with PBS 3 times, the bacteria were resuspended in PBS (180 μl) buffer, PI (10 μl, 20 μg/ml) dye was added, and the mixture was incubated for another 20 min in an ice bath. No compound was introduced as a blank control for laser scanning confocal microscope images.

### Effects of 9b on bacterial PGN and cell membrane phospholipids

The checkerboard dilution method [61,62] was used to measure the interactions of **9b** with bacterial cell wall PGN and cell membrane phospholipids. After the mixing of compound **9b** (50 μl, 0 to 128 μg/ml) with different concentrations of PG, PE, CL, and PNG (50 μl, 0 to 128 μg/ml) in equal amounts, it was followed by the addition of MRSA-16 bacterial suspension (100 μl, 1 × 10^5^ CFU/ml). The mixture was placed in a 96-well plate and incubated at 37 °C for 16 to 18 h before reading the results.

### ROS measurement

A monoclonal colony of MRSA-16 was cultured in 2 ml of MHB at 200 rpm for 5 to 7 h, harvested by centrifugation (5,000 rpm, 4 °C, 5 min), washed 3 times with PBS, and resuspended in an equal volume of dichlorodihydrofluorescein diacetate solution. After incubation at 37 °C for 40 min, the cells were centrifuged again and resuspended in PBS to remove excess dye. Then, 180 μl of bacterial suspension was mixed with 20 μl of **9b** at different concentrations and incubated at 37 °C for 1 h. Fluorescence intensity was recorded on a microplate reader (λex/λem = 488/530 nm). Each assay was performed in triplicate.

### Effects of exogenously added DNA on the anti-MRSA activity of 9b

Compound **9b** (50 μl, 0.5 μg/ml), genomic DNA (50 μl, 0 to 0.2 μg/ml), and vancomycin (50 μl, 1.0 μg/ml) were added into 96-well plates, and 100 μl of MRSA-16 suspension (6 × 10^5^ CFU/ml) was added. The final volume of each well was 200 ml, then incubated at 37 °C for another 14 to 18 h. OD_600_ (optical density at 600 nm) value of each well was determined to evaluate the growth of bacteria.

### Effects of different concentrations of 9b on pUC19 plasmid

To Luria–Bertani medium containing 100 μg/ml ampicillin, *E. coli* DH5α containing pUC19 was added, and then the mixture was cultured (37 °C, 200 rpm) for 12 h. Plasmid extraction was carried out according to the plasmid extraction kit, and 6 μl of different concentrations of **9b** (100, 75, 50, 25, 10, 5, 2.5, 1 μg/ml) were mixed with plasmid (1 μl, 150 ng/μl) and incubated at 37 °C away from light for 6 h. The plasmid (4.5 μl) was mixed with 10 × loading buffer (0.5 μl) using a 0.8% gel run at 150 V for 15 min. The gel bands were visualized and photographed for observation by a UV gel acquisition system.

### Effects of 9b on pUC19 plasmid over time

The compound **9b** (4.5 μl, 50 μg/ml) was mixed with 1 μl of plasmid (150 ng/μl), and then 4.5 μl of plasmid was mixed with 10 × loading buffer (0.5 μl) in each group at each time point of 0.5, 1, 1.5, 2, 3, 4, 5, and 6 h. The plasmid was run for 15 min at 150 V using a 0.8% gel. The gel bands were photographed and analyzed by a UV gel acquisition system.

### Product purification after 9b treatment of pUC19 plasmid

Different concentrations of **9b** (6 μl, 100, 75, 50 μg/ml) were mixed with 1 μl of plasmid (150 ng/μl), respectively, and then the mixture was incubated at 37 °C for 6 h without light. The product was purified by the product purification kit and then run for 15 min using a 0.8% gel at 150 V. The gel bands were observed and photographed for analysis by a UV gel acquisition system.

### Effects of 9b on genomic DNA

Cultured MRSA-16 was centrifuged (5,000 rpm, 4 °C, 5 min) and washed 3 times with PBS buffer. The MRSA-16 was resuspended in MHB containing different concentrations of **9b** (1 to 8 μg/ml) at 37 °C for 3 h. Genomic DNA was extracted using a genome kit and then analyzed by 0.8% gel running at 150 V for 15 min and visualized by a UV gel acquisition system.

### Transcriptome analysis of MRSA-16 treated with 9b

The monoclonal colony of MRSA-16 was picked up in MHB medium (2 ml) and incubated (37 °C, 200 rpm) for 12 h. On the next day, the MRSA bacterial solution was diluted 100-fold and cultured for another 3 to 6 h, and then **9b** was added to achieve a final concentration of 8 μg/ml, and the mixture was further incubated for 8 h. After centrifugation, the supernatant was collected and frozen in liquid nitrogen for 10 to 15 min and then transferred to −80 °C for refrigeration. Finally, the samples were measured by Shanghai Vina Biotechnology Co.

### Hemolytic activity

The detailed procedure for the determination of hemolytic activity (HC_50_) was according to our previously reported methods [33,62,63], and it can be found in the Supplementary Materials.

### Stability assay of 9b

To determine the stability of compound **9b** in plasma, we incubated different concentrations of **9b** in 50% plasma for 3 and 6 h. After incubation, we added bacterial suspension (150 μl) in 96-well plates and incubated at 37 °C for 16 to 24 h. After reading the MIC, the MBC was determined. The plasma stability of compound **9b**’s antibacterial effect was assessed through MBC variation over time.

### Cytotoxicity and membrane selectivity evaluation

The cytotoxicity and membrane selectivity of compound **9b** on LO2 and NIH3T3 cells were determined by the cell counting kit-8 method according to our previous reports [59,62].

### In vivo toxicity evaluation

All animal procedures complied with institutional ethical regulations (SYXK (X) 2022-0008) approved by the Animal Protection and Utilization Committees of Zhengzhou University and the University of South China.

### In vivo anti-MRSA activity

An in vivo mouse skin abscess infection model was used to test **9b**’s anti-MRSA effectiveness.

An established murine skin abscess model was employed to evaluate the anti-MRSA efficacy of compound **9b**. A total of 30 healthy female KM mice were randomly allocated into 4 groups: the blank group (0.9% NaCl), the infected control group (MRSA-16 only), the **9b**-treated groups (2.5 and 5 mg/kg), and the positive control group receiving vancomycin (5 mg/kg). After depilation of the dorsal area, mice were anesthetized and subcutaneously injected with MRSA-16 (60 μl, 6 × 10^8^ CFU/ml). Two hours later, each group received its designated treatment at the infected site. For the thigh infection model [64,65], another cohort of 30 female KM mice of similar body weight was randomly assigned into 5 treatment groups: the blank control, the infected control, the vancomycin group (10 mg/kg), the high-dose **9b** group (10 mg/kg), and the low-dose **9b** group (5 mg/kg). After removing hair from the left thigh and keeping the animals for 24 h, all mice except the blank controls were intramuscularly inoculated with MRSA-16 suspension (60 μl, 1 × 10^8^ CFU/ml). Two hours following infection, compound **9b** at different doses was injected locally at the infection site for two consecutive days with a 24-h interval. Equal volumes of saline were administered to the blank and infected control groups. Twenty-hour hours after the last injection, the animals were euthanized. The infected skin or thigh tissues, together with major organs (heart, liver, spleen, lungs, and kidneys), were collected, fixed in 4% paraformaldehyde, and embedded in paraffin for hematoxylin and eosin staining. Bacterial loads at the infection sites of each group were subsequently quantified using the drop-plate counting method to assess the in vivo antibacterial activity of compound **9b**.

### Statistical analysis

Results are expressed as means ± SD or means ± standard error of the mean (SEM), as indicated. All statistical evaluations were carried out using SPSS software (version 21.0; IBM Corp.). Differences between 2 groups were analyzed by 1-way analysis of variance (ANOVA), depending on the experimental design. A *P* value ≤ 0.05 was considered statistically significant. Levels of significance are denoted as *P < 0.05, **P < 0.01, ***P < 0.001, ****P < 0.0001. Each experiment was independently performed at least 3 times.

## Ethical Approval

All animal procedures were conducted in accordance with the National Institutes of Health (NIH) Guide for the Care and Use of Laboratory Animals and were approved by the Animal Protection and Utilization Committees of Zhengzhou University and the University of South China (approval no. SYXK (X) 2022-0008).

## Data Availability

All data supporting the findings of this study are available within the article and its Supplementary Materials. Additional raw data, including original microscopy images, MIC measurements, flow cytometry files, and uncropped gels, are available from the corresponding authors upon reasonable request.

## Supplementary Materials

Supplementary 1 Sections S1 to S11 Figs. S1 to S32 Tables S1 to S3
