# Supplementary material for: Overcoming Methicillin-Resistant Staphylococcus aureus Infections by Disrupting Membrane Integrity and Inducing DNA Degradation with a Dual-Mechanism Fluorescent Phenanthro[9,10-d]imidazole Molecule
Source: Research (Wash D C). 2026 Apr 17;9:1242. doi: 10.34133/research.1242 (PMC13087400; doi:10.34133/research.1242)
Supplement: Supplementary 1 — Sections S1 to S11 Figs. S1 to S32 Tables S1 to S3 [file research.1242.f1.doc]

**Supplementary Material**

**Overcoming methicillin-resistant *Staphylococcus aureus* (MRSA) infections by** **disrupting membrane integrity and inducing DNA degradation with a dual-mechanism fluorescent phenanthro[9,10-d]imidazole** **molecule**

Ting Xu,^a,#^ Xiaoting Yan,^b,#^ Tingting Wang,^b^ Liping Cui,^b^ Shangshang Qin,^b^ Ruige Yang,^a,b^,*Hong Yao,^c^,* and Yong Guo,^a,b^,*

*^a^Hunan Province Cooperative Innovation Center for Molecular Target New Drug Study, School of Pharmaceutical Science, Hengyang Medical School, University of South China, Hengyang, 421001, Hunan Province, China*

*^b^School of Pharmaceutical Sciences, Zhengzhou University, Zhengzhou 450001, Henan Province, China*

*^c^College of Veterinary Medicine, Henan Agricultural University, Zhengzhou 450046, Henan Province, China*

^#^T. Xu and X. Yan contributed equally to this work.

***Corresponding Authors**

E-mail: yrggg@163.com (R. Yang); yaoh0913@henau.edu.cn (H. Yao); guoyong_122@163.com (Y. Guo).

**Contents**

[1. DNA and protein leakage S3](#__RefHeading___Toc30073)

[2. DNA binding assay S3](#__RefHeading___Toc15107)

[3. Docking assay S3](#__RefHeading___Toc10642)

[4. LDH release activity testing S4](#__RefHeading___Toc7946)

[5. ITC assay S4](#__RefHeading___Toc10735)

[7. *In Vivo* toxicity evaluation S5](#__RefHeading___Toc9270)

[8. Molecular docking, molecular dynamics simulations, and MM/GBSA analysis S6](#__RefHeading___Toc28204)

[9. *In vivo* antibacterial visual monitoring assay. S7](#__RefHeading___Toc2263)

[10. The *in vitro* stability of compound **9b** in rat plasma determined by LC-MS/MS S8](#__RefHeading___Toc9826)

[11. Plasma protein binding rate S9](#__RefHeading___Toc16269)

[12. *In vivo* pharmacokinetic assay S10](#__RefHeading___Toc12120)

[13. Spectral data for compounds **5a**, **8a**, **9b**, **9a**, **9b**, **12a**, **14a**, **14b**, **18a**, **20a**, and **20b** S20](#__RefHeading___Toc15366)

[14. HPLC chromatograph of representative compounds **9a** and **9b** S26](#__RefHeading___Toc6141)

[11. Copies of spectra of compounds **5a**, **8a**, **9b**, **9a**, **9b**, **12a**, **14a**, **14b**, **18a**, **20a**, and **20b** S28](#__RefHeading___Toc31622)

**1. DNA and protein leakage**

The monoclonal colony of MRSA-16 was inoculated in MHB medium (2.0 mL) and incubated (200 rpm, 37 °C) for 5−6 h. The supernatant was washed three times with PBS buffer, the supernatant was discarded and resuspended with PBS buffer, and then compound **9b** was added to make the final concentrations of 8×, 4×, 2×, and 1× MIC, respectively. It was incubated at 37 °C for 4 h and then centrifuged for 4 min (3500 rpm, 4 °C). The supernatant was removed and the concentration of DNA was determined by microspectrophotometer and the concentration of protein was determined by BCA Protein Concentration Kit.

1. **DNA binding assay**

For the UV absorption and CD spectra of the DNA–**9b** complex, bacterial genomic DNA solution (50 μg/mL, working concentration) was treated with **9b** at a final concentration of 64 μg/mL and incubated at room temperature for 30 min. UV absorption and CD spectra were then recorded, with each measurement performed in triplicate. The titration assay was carried out in PBS (pH 7.4) at 37 °C. DNA (10 μg/mL) was mixed with SYTO green and incubated for 30 min, after which different concentrations of **9b** (1–32 μg/mL) were added to the DNA–dye complex. The mixtures were further incubated at 37 °C for 1 h to allow equilibrium. Fluorescence intensity of SYTO green was then recorded using a microplate reader.

1. **Docking assay**

The DNA sequence (CGCGAATTCGCG) was derived from the X-ray crystal structure (PDB ID: 4U8A). The receptor was prepared by defining the docking grid center at the position of the co-crystallized ligand with a grid size of 10–12 Å. Self-docking of the native ligand was performed to validate the grid’s ability to reproduce the crystallographic binding pose. Molecular docking was conducted using the CDOCKER module in Discovery Studio 2.55.

1. **LDH release activity testing**

LO2 cells were seeded into 96-well plates at a density of 1–2 × 10⁴ cells/well and cultured overnight until reaching 70–80% confluence. The medium was then replaced with serum-free, phenol red-free DMEM for 30 min equilibration. Experimental groups included control (DMSO), **9b** treatment (16 μg/mL), and 1% Triton X-100. Cells were incubated at 37 °C for 4 h. At the end of incubation, 50 μL of supernatant was collected from each well and transferred to a new 96-well plate, mixed with an equal volume of LDH reaction mixture, and incubated at room temperature for 20–30 min in the dark. Absorbance was measured at 490 nm using a microplate reader. All experiments were performed in triplicate and repeated at least three times.

**5. ITC assay**

PG (2 mM) and **9b** (0.2 mM) were dissolved in sterile water containing 5% DMSO. PG was slowly dripped into the calorimetric cell containing compound **9b** and repeated 25 times with an equilibrium interval of 120 seconds. The equilibrium dissociation constant (K_D_) and variations of entropy (Δ*S*) and enthalpy (Δ*H*) were calculated using the instrument software.

**6. Hemolytic activity**

Stock solutions of the tested compounds were prepared in phosphate buffered saline (PBS) at a concentration of 5120 μg/mL and serially diluted by the 2-fold dilution method, and then 50 μL of each dilution was placed in 96-well plates. RBCs were obtained from the fresh sterile sheep blood and resuspended in PBS to 5% (*v/v*). The RBC suspension (150 μL) was added to 96-well plates and then the plates were incubated at 37 °C for 1 h. The negative control was an RBC suspension with PBS only, and the positive control was PBS containing 0.1% (v/v) Triton X-100. After incubation, the mixture was centrifuged (3500 rpm for 5 min), and then the supernatant (100 μL) was pipetted into a fresh 96-well plate. Finally, hemolysis was calculated by measuring the absorbance at 540 nm.

**7. *In Vivo* toxicity evaluation**

Thirty healthy female KM mice aged 4−6 weeks was separated into five groups: the blank group (0.9 % NaCl) and the administered group (2.5, 5, 10, 20, and 40 mg/kg). Following back depilation, mice were injected with 60 μL of various concentrations of **9b** and 0.9 % NaCl solution 24 h later. After 24 h, the mice were observed for death and abnormalities such as redness, hardening, and ulceration of the skin. The mice in the highest administered dose group that exhibited no adverse effects were euthanized, and blood was collected from the eyeballs, and the skin as well as tissues at the administration site of the mice were aseptically isolated for autopsy. For the mouse thigh model, thirty healthy female KM mice with the same body weight were randomly divided into five groups, the left leg was depilated, and after 24 h of rearing, the leg was injected intramuscularly with 60 μL of different concentrations of **9b** solution (5, 10, 15, 20 mg/kg), the control group was injected with an equal amount of saline, and injected for two days consecutively with an interval of 24 h. The survival status of mice and adverse reactions such as redness, swelling and ulceration of legs were observed for seven consecutive days after injection. Blood samples were tested for the blood routine and blood biochemical indexes, and samples of skin, heart, liver, spleen, lungs, and kidneys were taken for H&E staining to evaluate the differences between the mice and those in the blank group.

**8. Molecular docking, molecular dynamics simulations, and MM/GBSA analysis**

The three-dimensional structures of compound **9b** and PG were constructed using Chem3D 20.0 and energy-minimized under the MMFF94 force field. Molecular docking was performed using AutoDock Vina v1.2.3, and all molecules were converted to the required PDBQT format using ADFRsuite v1.02 prior to docking. The exhaustiveness parameter was set to 32, while other parameters were kept at default values. The top-ranked docking pose was selected as the representative binding conformation and visualized using PyMOL v2.5.2 for subsequent analyses and MD simulations.

All-atom MD simulations were carried out using AMBER 24. AM1-BCC charges for compound **9b** were generated with the antechamber module. The GAFF2 force field was applied for the small molecule, while the OL15 force field was used for DNA where applicable. Systems were solvated in a truncated octahedral TIP3P water box extending 10 Å from the solute, and Na⁺/Cl⁻ ions were added to neutralize the system. Energy minimization was performed using 2500 steps of steepest descent followed by 2500 steps of conjugate gradient minimization. The system was then gradually heated from 0 to 298.15 K over 200 ps under constant volume conditions, followed by 500 ps equilibration in the NVT ensemble and an additional 500 ps equilibration in the NPT ensemble. Production MD simulations were conducted for 100 ns under NPT conditions with periodic boundary conditions. A cutoff of 10 Å was applied for nonbonded interactions, long-range electrostatics were treated using the particle mesh Ewald (PME) method, hydrogen bonds were constrained using the SHAKE algorithm, and temperature was controlled using Langevin dynamics with a collision frequency of 2 ps⁻¹. The pressure was maintained at 1 atm, the integration time step was set to 2 fs, and coordinates were saved every 10 ps for analysis.

Binding free energies were calculated using the MM/GBSA method based on snapshots extracted from the last 10 ns (90–100 ns) of the MD trajectories. The binding free energy (ΔG_bind) was calculated as the difference between the complex and the sum of receptor and ligand free energies, including van der Waals, electrostatic, and solvation contributions. The polar solvation energy was estimated using the generalized Born model (*igb* = 2), while the nonpolar solvation energy was calculated from the solvent-accessible surface area using a surface tension coefficient of 0.0072 kcal·mol⁻¹·Å⁻². Entropic contributions were not included due to their high computational cost and limited accuracy.

1. ***In vivo* antibacterial visual monitoring assay.**

A mouse skin abscess model was employed to evaluate the *in vivo* fluorescence monitoring performance of compound **9b**. Twenty healthy female KM mice were randomly divided into four groups: saline group (uninfected with MRSA‑16), **9b**‑treated group (uninfected with MRSA‑16), infection control group (infected with MRSA‑16 and treated with saline), and **9b**‑treated group (5 mg/kg, infected with MRSA‑16). After depilation of the dorsal region, mice were anesthetized and subcutaneously injected with 60 μL of MRSA‑16 suspension (6×10⁸ cfu/mL) or an equivalent volume of saline. 2 h post‑infection, the corresponding treatments were administered at the injection site in each group. *In vivo* fluorescence imaging was performed under excitation at 260 nm at 1, 6, and 24 h post‑treatment, respectively. Meanwhile, skin tissues from the infected sites were collected and homogenized, and the bacterial burden at the infection foci was quantified using the plate counting method.

1. **The *in vitro* stability of compound 9b in rat plasma determined by LC-MS/MS**

Aliquots of blank heparin sodium-anticoagulated rat plasma were spiked with the working solutions in centrifuge tubes and vortexed thoroughly to achieve final concentrations of 4 μg/mL for **9b** and 1 μM for propantheline bromide. The spiked plasma samples were incubated in a water bath at 37 ℃. At predetermined time points (0 min, 0.5 h, 1 h, 2 h, and 4 h), 20 μL of the incubation mixture was collected and mixed with 300 μL of internal standard-containing protein precipitant, followed by vigorous vortexing to facilitate protein precipitation. All samples were centrifuged at 5500 g for 10 min. Subsequently, 150 μL of the supernatant was mixed with an equal volume of ultrapure water and vortexed, then subjected to LC-MS/MS analysis using the method detailed in the table below.

| Chromatography column | Welch Ultimate AQ-C18, 2.1*50 mm, 5 μm | | |
| --- | --- | --- | --- |
| Mobile phase | Mobile phase A： | 0.1% formic acid-water | |
|  | Mobile phase B： | acetonitrile | |
| Ionization mode | ESI/Positive | | |
| Scanning method | MRM | | |
| Flow rate | 0.6000 mL/min | | |
| Analyte | **9b** | 464.3/324.2 | |
| Internal standard | Carbamazepine | 237.3/194.2 | |
| Liquid phase gradient | Time (min) |  | B% |
|  | 0.01 |  | 15 |
|  | 1.20 |  | 95 |
|  | 2.00 |  | 95 |
|  | 2.01 |  | 15 |
|  | 3.00 |  | Stop |

The stability was evaluated by the parent drug residual rate, calculated as:Residual rate (%)=A_t_/A_0_×100%, where A_t_ is the analyte amount at each incubation time point and A_0_ is the analyte amount at 0 h. The half-life (t_1/2_) was determined using the formula t_1/2_=0.693/k, where k is the rate constant derived from linear regression of the natural logarithm of the residual rate against incubation time.

1. **Plasma protein binding rate**

The plasma protein binding rate of **9b** in rat plasma was determined by ultracentrifugation combined with LC-MS/MS analysis, with warfarin sodium serving as the positive control. A phosphate buffer (PB) containing 0.002% Tween-80 was prepared as the basic buffer system. Working solutions of compound **9b** and warfarin sodium were prepared with 80% acetonitrile-water solution, and then spiked into blank EDTA-2K anticoagulated male SD rat plasma (Lot No.: R1-P-20260116-EDTA-2K-M-294~301), thus preparing plasma samples with a final concentration of 4 μg/mL for **9b** (purity > 98%) and 2 μM for warfarin sodium (Lot No.: 101163-201702, purity 91.70%). The spiked plasma samples were incubated at 37 ℃ and then subjected to ultracentrifugation at 800,000 g for 4 h. After centrifugation, 20 μL of the supernatant was collected, and 20 μL of the remaining centrifuged mixture after pipetting and mixing was taken as the recovery sample. Separately, 20 μL of the T_0_ sample and the recovery sample were each mixed with 90 μL of 0.002% Tween-80-PB buffer, vortexed for 10 min, and then 50 μL of the mixture was added to 300 μL of internal standard-containing precipitant. In addition, 20 μL of the ultracentrifugation supernatant was mixed with 20 μL of blank plasma, vortexed for 10 min, and then added to 300 μL of internal standard-containing precipitant. All samples were vortexed and subsequently centrifuged at 5500 g for 10 min. Then, 150 μL of the supernatant was mixed with an equal volume of ultrapure water and vortexed for homogenization, followed by injection analysis via the LC-MS/MS method. Chromatographic separation was performed on an AQ-C18 column (2.1×50 mm, 5 μm) with the mobile phase consisting of 0.1% formic acid-water (Phase A) and acetonitrile (Phase B). Mass spectrometry was operated in electrospray ionization positive mode (ESI+) with multiple reaction monitoring (MRM) scanning, and the monitored ion transition for **9b** was set at m/z 464.3→324.2. The unbound fraction (F_u_, %) was calculated according to the formula F_u_ (%) = C_T_/C_0_ (where C_T_ is the drug concentration in the ultracentrifugation supernatant and C_0_ is the drug concentration in the T_0_ sample). The plasma protein binding rate (%) was calculated as Plasma protein binding rate (%) = 1 − F_u_ (%), and the sample recovery rate (%) was determined by the formula Recovery (%) = C_r_/C_0_ (where C_r_ is the drug concentration in the recovery sample).

1. ***In vivo* pharmacokinetic assay**

SPF grade SD rat (320–350 g) used in this study were purchased from Hunan Slike Jingda Laboratory Animal Co., Ltd. All rats were acclimatized to a standard, environmentally controlled animal room (temperature, 18– 29 °C; relative humidity of 30 –70% and a 12 h/12 h light/dark cycle) for 1 week before the experiment. Adequate water and food were provided. Three SD rats were used for **9b** group. **9b** was (2.5 mg/kg) dissolved in DMSO/Tween 80/saline (5/5/90, *v/v/v*）to a concentration of 0.2 mg/mL, and was given to each group by intravenous administration, respectively. Blood samples were collected at 0 min, 5 min, 10 min, 15 min, 30 min, 1 h, 2 h, 4 h, 8 h and 24 h after administration (anticoagulant: EDTA-2K). 100 μL of solvent of methanol/acetonitrile (1:1, *v/v*) was added to 10 μL of plasma and vortexed thoroughly. It was centrifuged for 5 min, then 20 μL of the supernatant was mixed with 20 μL of water for analysis. Samples were analyzed by LC-MS-012. The Welch Ultimate® AQ-C18（2.1×50 mm，5 μm）was used for the analysis. Gradient elution was applied consisting of 0.1% formic acid - water (A phase) and acetonitrile (B phase). After analyzing the concentrations of compounds, the value of T1/2, AUClast, AUCINF_obs, CL_obs, animal using Phoenix WinNonlin (CERTARA, USA).

**Table S1. Binding free energies and energy components predicted by MM/GBSA (****kcal/mol).**

| System name | **9b**-PG |
| --- | --- |
| ΔE_vdw_ | -21.26±1.75 |
| ΔE_elec_ | -30.92±3.94 |
| ΔG_GB_ | 33.02±4.76 |
| ΔG_SA_ | -2.81±0.25 |
| ΔG_bind_ | -21.97±2.42 |

ΔE_vdW_: van der Waals energy.

ΔE_elec_: electrostatic energy.

ΔG_GB_: electrostatic contribution to solvation.

ΔG_SA_: non-polar contribution to solvation.

ΔG_bind_: binding free energy.

MRTINF_obs, VSS_obs was calculated from time concentration curves in each

**Table S2. *In Vivo* Toxicity of Compound 9b**

| Dose (mg/kg/d) | number | Subcutaneous injection |
| --- | --- | --- |
|  |  | Survival rate (%) |
| Control | 5 | 100% |
| 40 | 5 | 100% |
| 20 | 5 | 100% |
| 10 | 5 | 100% |
| 5 | 5 | 100% |
| 2.5 | 5 | 100% |

**Table S3. *In Vivo* Toxicity of Compound 9b**

| Dose (mg/kg/d) | number | Thigh muscle injection |
| --- | --- | --- |
|  |  | Survival rate (%) |
| Control | 5 | 100% |
| 20 | 5 | 100% |
| 25 | 5 | 100% |
| 10 | 5 | 100% |
| 5 | 5 | 100% |

**Fig. S1.** The fluorescence quantum yield of **9b**, using quinidine sulfate as the standard control (in the absence of bacterial environment, with excitation wavelength of 350 nm)


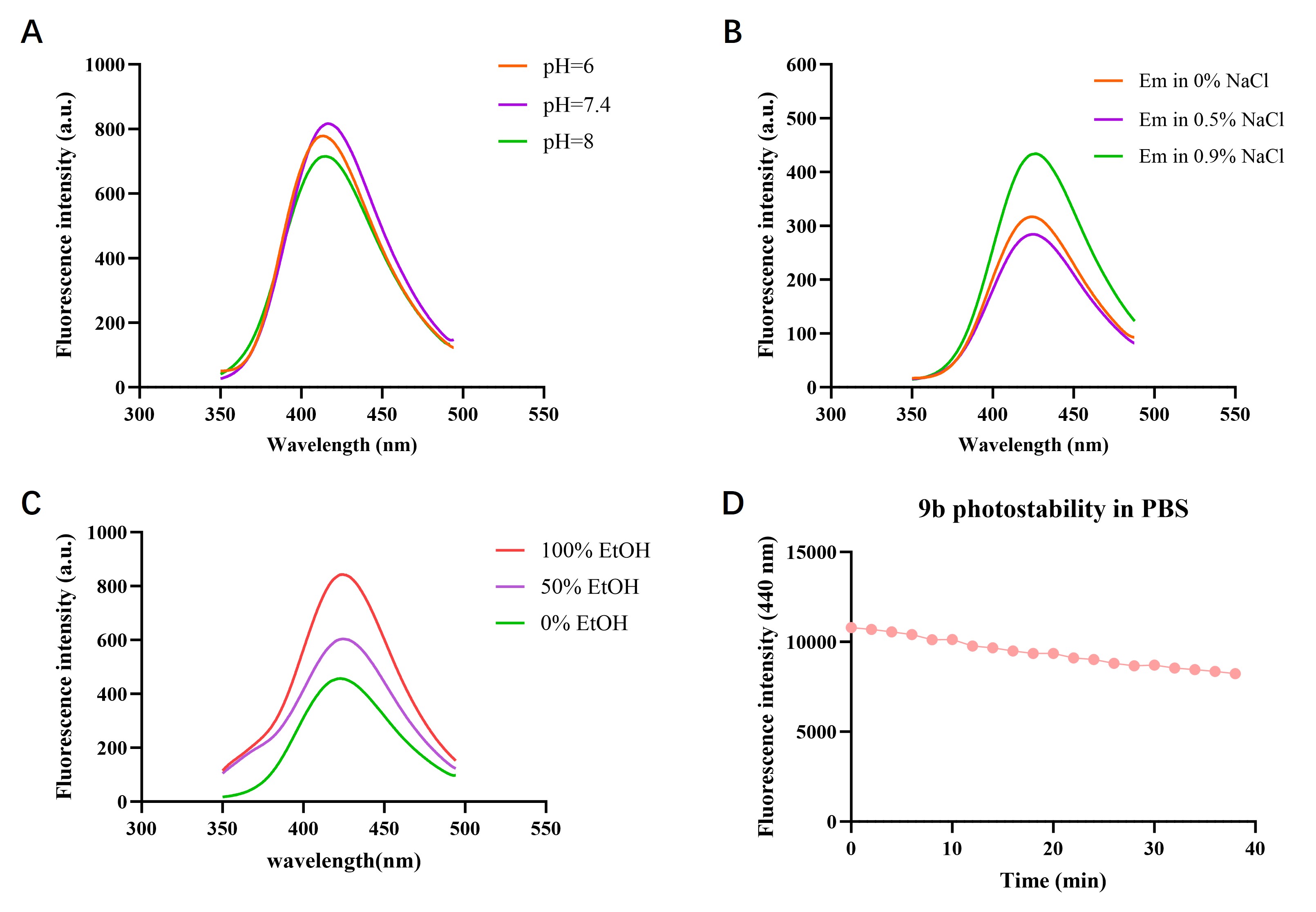


**Fig. S2.** Photophysical characterization of **9b** under biologically relevant conditions. (A) Fluorescence emission spectra of **9b** recorded at different pH values (pH 6.0, 7.4, and 8.0); (B) Fluorescence emission spectra of **9b** in NaCl solutions of varying ionic strength (0%, 0.5%, and 0.9% NaCl); (C) Emission spectra of **9b** in solvent mixtures containing different ethanol fractions (0%, 50%, and 100% EtOH), indicating solvent polarity–dependent fluorescence enhancement; (D) Photostability assessment of **9b** in PBS under continuous excitation at 440 nm. Fluorescence intensity was monitored over 40 min.


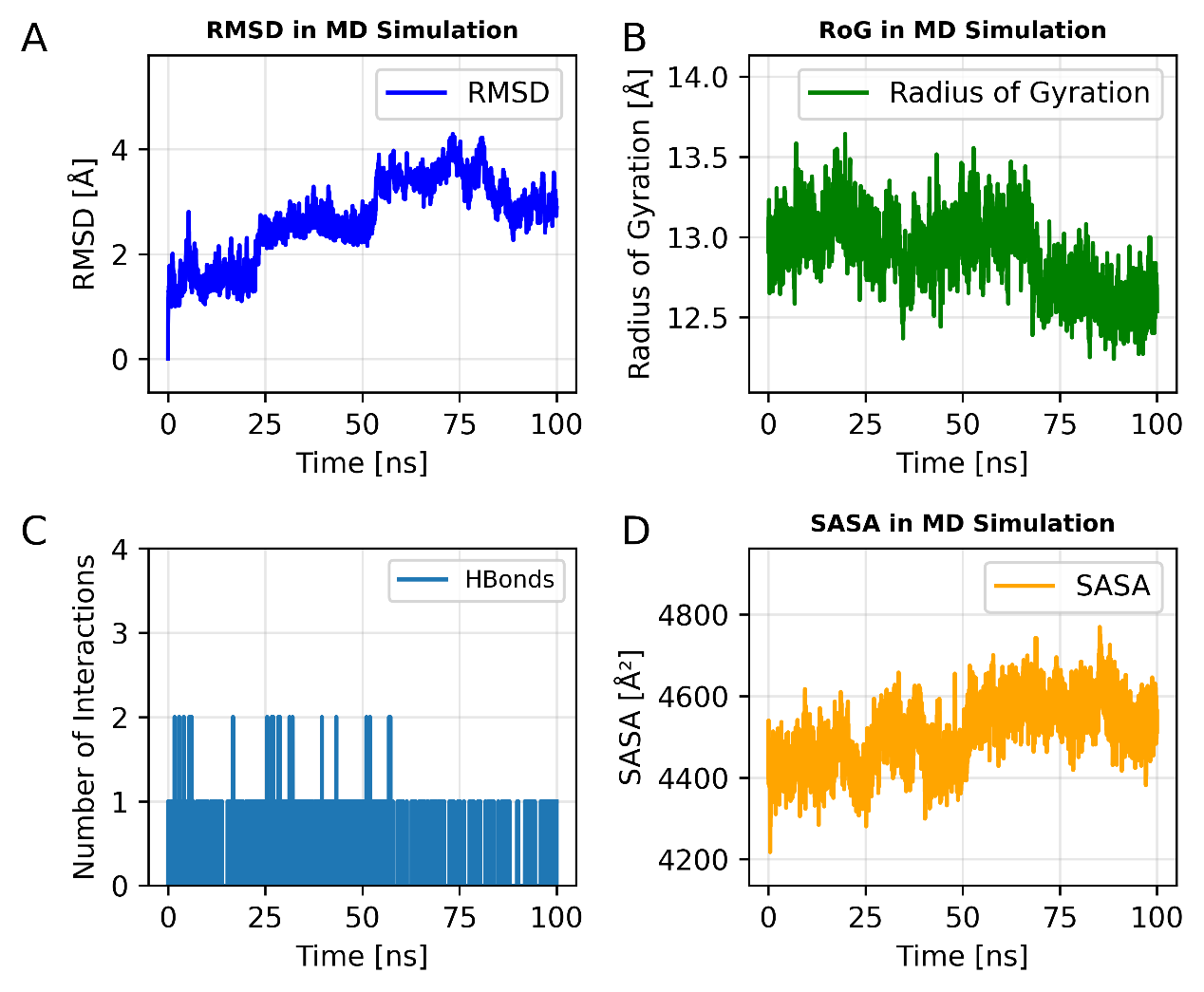


**Fig. S3.** Molecular dynamics (MD) analysis of the **9b**–DNA complex. (A) Root-mean-square deviation (RMSD) of the **9b**–DNA complex during the 100 ns MD simulation. (B) Radius of gyration (Rg) of the complex over the simulation time. (C) Number of hydrogen bonds formed between compound **9b** and DNA throughout the simulation. (D) Solvent-accessible surface area (SASA) of the **9b**–DNA complex as a function of simulation time.

**Fig. S4.** The influence of eliminating reactive oxygen species on DNA oxidative damage during the **9b** antibacterial process


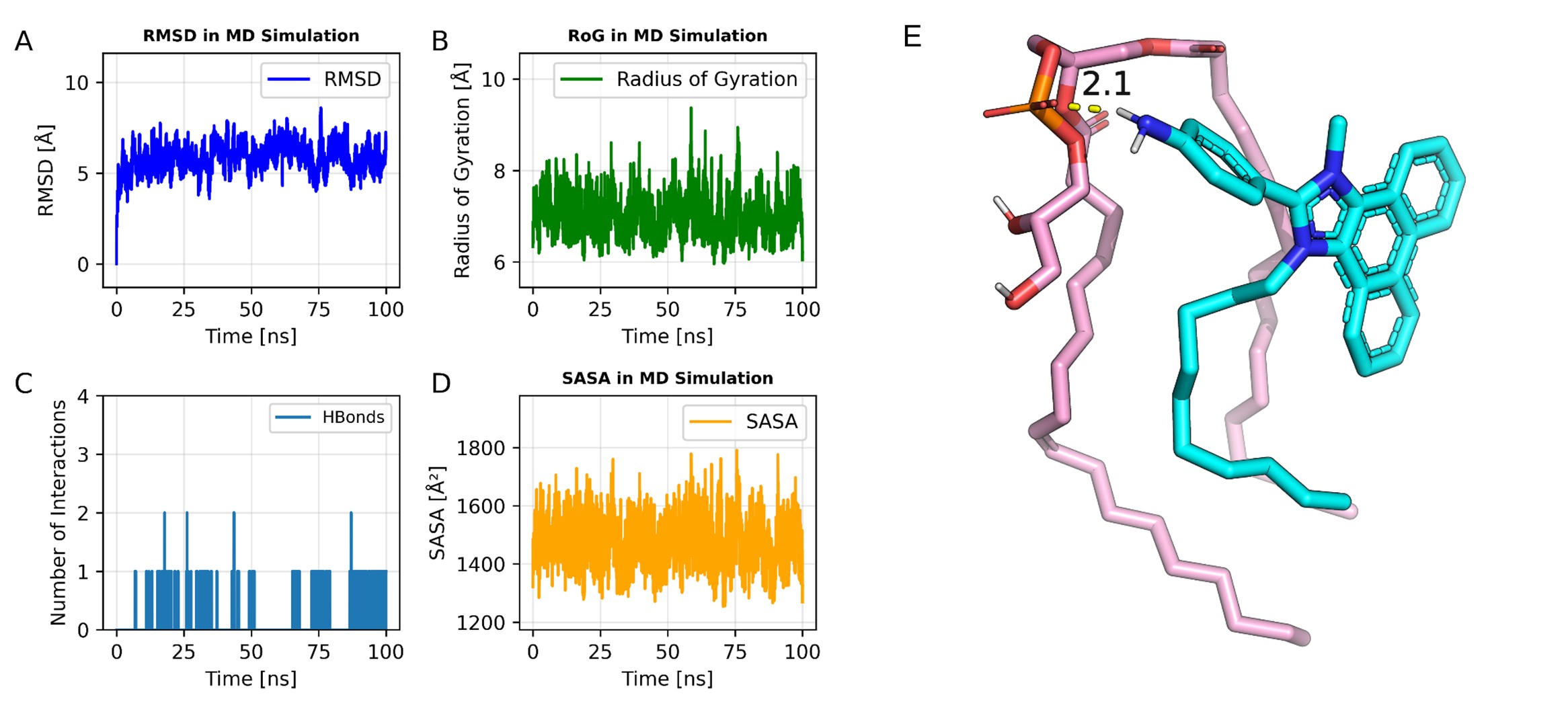


**Fig. S5.** Molecular dynamics (MD) analysis of the **9b**–PG complex. (A) RMSD of the **9b**–DNA complex during the 100 ns MD simulation. (B) Rg of the complex over the simulation time. (C) Number of hydrogen bonds formed between compound **9b** and DNA throughout the simulation. (D) SASA of the **9b**–DNA complex as a function of simulation time. (E) The binding pattern of the **9b**–PG complex. Pink represents PG, cyan represents **9b**, and the yellow dotted lines indicate hydrogen bonding.

**Fig. S6.** The quantitative dose–response hemolysis curve of **9b**.

**Fig. S7.** The plasma stability of **9b**.


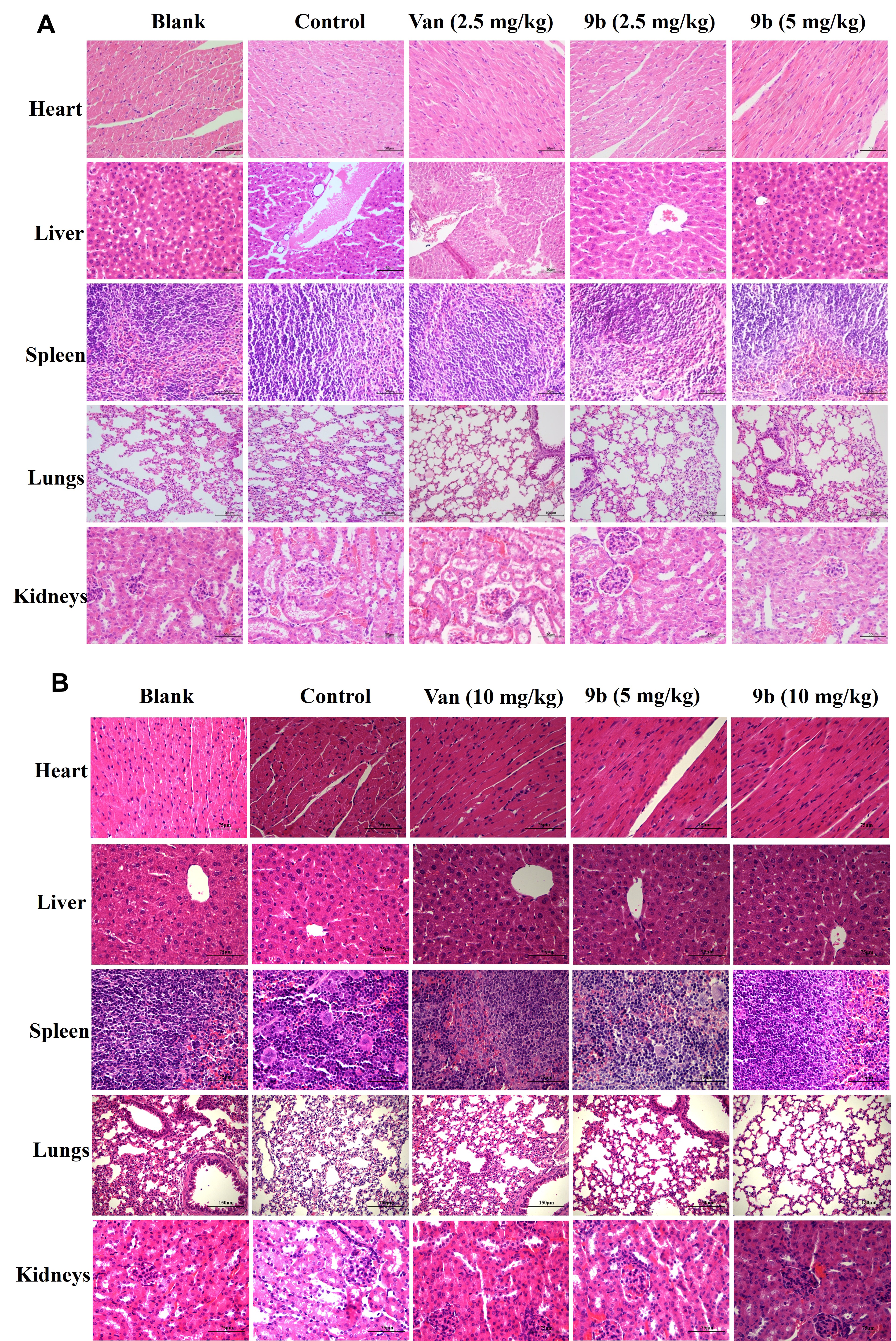


**Fig. S8.** (A) Representative H&E-stained sections from major organs after various treatments in a MRSA-infected mouse skin abscess model. Scale bar is 50 μm (for heart, liver, spleen, and kidneys) or 100 μm (for lungs). (B) Representative H&E-stained sections from major organs after various treatments in a thigh model of mice. Scale bar is 75 μm (for heart, liver, spleen, kidneys, and leg muscle) or 150 μm (for lungs).

**13.Spectral data for compounds 5a, 8a, 9b, 9a, 9b, 12a, 14a, 14b, 18a, 20a, and 20b**

*N,N,N-trimethyl-4-(1H-phenanthro[9,10-d]imidazol-2-yl)benzenaminium iodide* (**5a**) Yield: 29.0 %, white solid, ^1^H NMR (400 MHz DMSO-d_6_) *δ*: 13.76 (s, 1H, −NH−), 8.88 (s, 2H, −Ph), 8.53**−**8.60 (m, 4H, −Ph), 8.20 (d, *J* = 8.8 Hz, 2H, −Ph), 7.67−7.75 (m, 4H, −Ph), 3.69 (s, 9H, N−CH_3_); ^13^C NMR (100 MHz DMSO-d_6_) *δ*: 148.1, 146.7, 139.6, 128.1, 128.0, 127.6, 126.4, 124.2, 122.1, 121.6, 56.6; HRMS (ESI) C_24_H_22_IN_3_ [M−I]^+^ calcd = 352.1808 ; found = 352.1807.

*N,N,N-trimethyl-4-(1-(3-methylbut-2-en-1-yl)-1H-phenanthro[9,10-d]imidazol-2-yl)benzenaminium iodide* (**8a**) Yield: 51.5 %, brown solid, ^1^H NMR (400 MHz DMSO-d_6_) *δ*: 9.02−9.06 (m, 1H, −Ph), 8.93 (d, *J* = 7.6 Hz, 1H, −Ph), 8.64 (d, *J* = 7.2 Hz, 1H, −Ph), 8.31−8.38 (m, 3H, −Ph), 8.10−8.18 (m, 2H, −Ph), 7.75−7.81 (m, 4H, −Ph), 5.57 (s, 1H, −CH=CH_2_), 5.28 (s, 2H, −CH_2_−), 3.74 (s, 9H, N−CH_3_), 1.77 (s, 3H, −CH_3_), 1.75 (s, 3H, −CH_3_); ^13^C NMR (100 MHz DMSO-d_6_) *δ*: 148.1, 146.7, 136.6, 131.3, 128.5, 127.9, 127.8, 127.6, 127.4, 126.4, 125.8, 123.7, 121.8, 121.6, 121.2, 119.6, 56.5, 46.1, 25.2, 18.3; HRMS (ESI) C_29_H_30_IN_3_ [M−I]^+^ calcd = 420.2434; found = 420.2437.

*4-(1-decyl-1H-phenanthro[9,10-d]imidazol-2-yl)-N,N,N-trimethylbenzenaminium iodide* (**8b**) Yield: 38.2 %, yellow solid, ^1^H NMR (400 MHz DMSO-d_6_) *δ*: 8.98 (d, *J* = 8.4 Hz, 1H, −Ph), 8.87 (d, *J* = 8.4 Hz, 1H, −Ph), 8.57 (d, *J* = 7.6 Hz, 1H, −Ph), 8.42 (d, *J* = 8.0 Hz, 1H, −Ph), 8.23 (d, *J* = 8.8 Hz, 2H, −Ph), 8.06 (d, *J* = 8.8 Hz, 2H, −Ph), 7.65−7.81 (m, 4H, −Ph), 4.68 (t, *J* = 7.2 Hz, 2H, −CH_2_−), 3.73 (s, 9H, N−CH_3_), 1.83 (s, 2H, −CH_3_), 1.06−1.18 (m , 14H, −CH_2_−), 0.78 (t, *J* = 7.2 Hz, 3H, −CH_3_); ^13^C NMR (100 MHz DMSO-d_6_) *δ*: 150.7, 147.8, 131.2, 128.4, 127.6, 127.49, 127.4, 127.3, 126.0, 125.7, 125.3, 122.7, 121.1, 56.6, 46.8, 31.2, 28.6, 25.3, 21.9, 13.8; HRMS (ESI) C_34_H_42_IN_3_ [M−I]^+^ calcd = 492.3373; found = 492.3377.

*2-(4-aminophenyl)-3-methyl-1-(3-methylbut-2-en-1-yl)-1H-phenanthro[9,10-d]imidazol-3-ium iodide* (**9a**) Yield: 36.4 %, yellow solid, ^1^H NMR (400 MHz DMSO-d_6_) *δ*: 9.12 (d, *J* = 7.6 Hz, 2H, −Ph), 8.77 (d, *J* = 4.4 Hz, 1H, −Ph), 8.46 (d, *J* = 7.6 Hz, 1H, −Ph), 7.88−7.92 (m, 4H, −Ph), 7.47−7.56 (m, 2H, −Ph), 6.86−6.88 (m, 2H, −Ph), 5.44 (s, 1H, −CH=CH_2_), 5.30 (s, 2H, −CH_2_−), 4.28 (s 3H, N−CH_3_), 1.71 (s, 3H, −CH_3_), 1.65 (s, 3H, −CH_3_); ^13^C NMR (100 MHz DMSO-d_6_) *δ*: 152.9, 150.8, 137.8, 132.2, 129.1, 128.4, 127.9, 126.4, 125.1, 124.7, 124.6, 122.6, 122.5, 120.7, 120.2, 117.8, 111.5, 105.7, 45.3, 38.0, 25.2, 18.1; HRMS (ESI) C_27_H_26_IN_3_ [M−I]^+^ calcd = 392.2121; found = 392.2124.

*2-(4-aminophenyl)-1-decyl-3-methyl-1H-phenanthro[9,10-d]imidazol-3-ium iodide* (**9b**) Yield: 28.3 %, yellow solid, ^1^H NMR (400 MHz DMSO-d_6_) *δ*: 9.12−9.14 (m, 2H, −Ph), 8.77−8.79 (m, 1H, −Ph) 8.55 (d, *J* = 7.6 Hz, 1H, −Ph), 7.88−7.95 (m, 4H, −Ph) 7.49−7.59 (m, 2H, −Ph) 6.86 (d, *J* = 8.8 Hz, 2H, −Ph), 6.15 (s, 2H, −NH_2_−), 4.71 (t, *J* = 7.2 Hz, 2H, −CH_2_−), 4.25 (s, 3H, N−CH_3_), 1.80−1.87 (m, 2H, −CH_2_−), 1.10−1.23 (m, 14H, −CH_2_−), 0.81 (t, *J* = 7.2 Hz, 3H, −CH_3_); ^13^C NMR (100 MHz DMSO-d_6_) *δ*: 152.8, 150.8, 132.2, 129.3, 129.2, 128.7, 128.4, 127.9, 127.8, 126.6, 124.8, 124.7, 124.5, 122.6, 122.2, 120.8, 120.3, 113.6, 106.0, 48.3, 37.9, 31.2, 28.6, 28.58, 28.5, 28.4, 28.0, 25.1, 22.0, 13.9; HRMS (ESI) C_32_H_38_IN_3_ [M−I]^+^ calcd = 464.3060; found = 464.3067.

*N-(2-aminoethyl)-4-(1H-phenanthro[9,10-d]imidazol-2-yl)benzamide* (**12a**) Yield: 61.9 %, yellow solid, ^1^H NMR (400 MHz DMSO-d_6_) *δ*: 8.88 (s, 1H, −Ph), 8.85 (s, 1H, −NH−), 8.60 (d, *J* = 7.6 Hz, 3H, −Ph), 8.40 (d, *J* = 8.4 Hz, 2H, −Ph), 8.07 (d, *J* = 8.0 Hz, 2H, −Ph), 7.73−7.77 (m, 2H, −Ph), 7.63 (t, *J* = 7.6 Hz, 2H, −Ph), 3.32−3.36 (m, 2H, −CH_2_−), 2.74 (t, *J* = 6.4 Hz, 2H, −CH_2_−); ^13^C NMR (100 MHz DMSO-d_6_) *δ*: 165.8, 148.2, 134.7, 132.5, 127.8, 127.7, 127.1, 125.7, 125.4, 123.9, 122.0, 42.6, 41.0; HRMS (ESI) C_24_H_21_N_4_O [M+H]^+^ calcd = 381.1715; found = 381.1711.

*N-(2-aminoethyl)-4-(1-(3-methylbut-2-en-1-yl)-1H-phenanthro[9,10-d]imidazol-2-yl)benzamide* (**14a**) Yield: 39.2 %, yellow solid, ^1^H NMR (400 MHz CDCl_3_) *δ*: 8.77 (d, *J* = 4.0 Hz, 1H, −Ph), 8.71 (d, *J* = 8.0 Hz, 1H, −Ph), 8.65 (d, *J* = 8.0 Hz, 1H, −Ph), 8.19 (s, 1H, −NH−), 7.97 (d, *J* = 8.0 Hz, 2H, −Ph), 7.83 (d, *J* = 6.8 Hz, 2H, −Ph), 7.58−7.67 (m, 4H, −Ph), 7.42 (d, *J* = 5.6 Hz, 1H, −Ph), 5.54 (s, 1H, −CH=CH_2_), 5.05 (s, 2H, −CH_2_−) 3.59 (d, *J* = 5.2 Hz, 2H, −CH_2_−), 3.02 (s, 2H, −CH_2_−),1.80 (s, 3H, −CH_3_), 1.69 (s 3H, −CH_3_); ^13^C NMR (100 MHz CDCl_3_) *δ*: 167.6, 151.0, 137.4, 136.4, 134.7, 133.2, 129.7, 129.0, 128.0, 127.7, 127.0, 126.9, 126.5, 125.4, 124.8, 124.0, 122.9, 122.3, 121.1, 120.3, 40.4, 29.6, 25.5, 18.4, 14.1; HRMS (ESI) C_29_H_29_N_4_O [M+H]^+^ calcd = 449.2341; found = 449.2339.

*N-(2-aminoethyl)-4-(1-decyl-1H-phenanthro[9,10-d]imidazol-2-yl)benzamide* (**14b**) Yield: 34.3 %, write solid, ^1^H NMR (400 MHz DMSO-d_6_) *δ*: 8.97 (d, *J* = 8.4 Hz, 1H, −Ph), 8.85 (d, *J* = 8.4 Hz, 1H, −Ph), 8.69 (s, 1H, −NH−), 8.58 (d, *J* = 7.6 Hz, 1H, −Ph), 8.40 (d, *J* = 8.4 Hz, 1H, −Ph), 8.08−8.12 (m, 2H, −Ph), 7.88 (d, *J* = 7.6 Hz, 2H, −Ph), 7.65−7.77 (m, 4H, −Ph), 4.70 (s, 2H, −CH_2_−), 3.36−3.39 (m, 2H, −CH_2_−) 2.77 (t, *J* = 6.0 Hz, 2H, −CH_2_−), 1.77 (d, *J* = 5.6 Hz, 2H, −CH_2_−), 1.01−1.18 (m, 14H, −CH_2_−), 0.78 (t, *J* = 7.2 Hz, 3H, −CH_3_); ^13^C NMR (100 MHz DMSO-d_6_) *δ*: 165.7, 151.7, 137.3, 135.0, 133.1, 129.6, 128.3, 127.6, 127.5, 127.3, 126.7, 125.9, 125.6, 125.1, 124.5, 123.5, 122.8, 121.8, 121.2, 46.3, 41.8, 40.7, 31.2, 29.3, 28.6, 28.57, 28.5, 28.0, 25.2, 21.9, 13.8; HRMS (ESI) C_34_H_41_N_4_O [M+H]^+^ calcd = 521.3280; found = 521.3282.

.

*2-(4-(1H-phenanthro[9,10-d]imidazol-2-yl)benzamido)ethan-1-aminium* (**18a**) Yield: 32.3 %, yellow solid, ^1^H NMR (400 MHz DMSO-d_6_) *δ*: 9.03 (t, *J* = 5.6 Hz, 1H, −NH−), 8.92−8.97 (m, 4H, −Ph), 8.72 (d, *J* = 8.4 Hz, 2H, −Ph), 8.21 (d, *J* = 8.4 Hz, 2H, −Ph), 8.16 (s, 2H, −NH_2_), 7.74−7.84 (m, 4H, −Ph), 3.58−3.63 (m, 2H, −CH_2_−) 3.04−3.08 (m, 2H, −CH_2_−), ^13^C NMR (100 MHz DMSO-d_6_) *δ*: 165.9, 146.8, 138.8, 128.3, 128.1, 127.7, 127.5, 126.8, 124.1, 122.9, 38.5, 37.2; HRMS (ESI) C_24_H_21_ClN_4_O [M−Cl]^+^ calcd = 381.1715; found = 381.1714.

*2-(4-(1-(3-methylbut-2-en-1-yl)-1H-phenanthro[9,10-d]imidazol-2-yl)benzamido)ethan-1-aminium chloride* (**20a**) Yield: 47.5%, yellow solid ^1^H NMR (400 MHz DMSO-d_6_) *δ*: 9.19 (s, 1H, −Ph), 9.05 (s, 1H, −Ph), 8.95 (s, 1H, −Ph), 8.85 (s, 1H, −NH−), 8.38 (s, 1H, −Ph), 8.28 (s, 4H, −Ph), 8.01 (s, 2H, −NH_2_), 7.80 (s, 4H, −Ph), 5.59 (s, 2H, −CH=CH_2_), 5.29 (s, 2H, −CH_2_−), 3.62 (s, 2H, −CH_2_−), 3.06 (s, 2H, −CH_2_−), 1.76 (s, 3H, −CH_3_), 1.71 (s, 3H, −CH_3_); ^13^C NMR (100 MHz DMSO-d_6_) *δ*: 165.8, 152.3, 136.2, 130.2, 128.9, 128.2, 128.0, 127.8, 127.2, 126.6, 125.9, 124.7, 124.0, 122.5, 121.9, 118.9, 38.5, 37.3, 25.4, 18.3; HRMS (ESI) C_29_H_29_ClN_4_O [M−Cl]^+^ calcd = 449.2336; found = 449.2340.

*2-(4-(1-decyl-1H-phenanthro[9,10-d]imidazol-2-yl)benzamido)ethan-1-aminium chloride* (**20b**) Yield: 45.6 %, write solid, ^1^H NMR (400 MHz DMSO-d_6_) *δ*: 9.18 (t, *J* = 4.8 Hz, 1H, −NH−), 9.07 (d, *J* = 8.0 Hz, 1H, −Ph), 8.98 (d, *J* = 8.4 Hz, 1H, −Ph), 8.83 (d, *J* = 7.6 Hz, 1H, −Ph), 8.50 (d, *J* = 8.4 Hz, 1H, −Ph), 8.28 (d, *J* = 8.4 Hz, 1H, −Ph), 8.25 (s, 2H, −NH_2_), 8.04 (d, *J* = 8.0 Hz, 2H, −Ph), 7.79−7.91 (m, 4H, −Ph), 4.75 (t, *J* = 7.2 Hz, 2H, −CH_2_−), 3.60−3.64 (m, 2H, −CH_2_−) 3.04−3.08 (m, 2H, −CH_2_−), 1.87 (t, *J* = 6.8 Hz, 2H, −CH_2_−), 1.06−1.20 (m, 14H, −CH_2_−), 0.79 (t, *J* = 7.2 Hz, 3H, −CH_3_); ^13^C NMR (100 MHz DMSO-d_6_) *δ*: 165.7, 154.0, 136.5, 130.5, 128.9, 128.4, 128.2, 128.1. 128.06, 128.0, 125.2, 124.8, 124.0, 122.5, 121.8, 47.2, 38.8, 38.4, 37.2, 31.1, 28.8, 28.7, 28.6, 28.5, 28.0, 25.1, 21.9, 13.9; HRMS (ESI) C_34_H_41_ClN_4_O [M−Cl]^+^ calcd = 521.3275; found = 521.3279.

**14. HPLC chromatograph of representative compounds 9a and 9b**


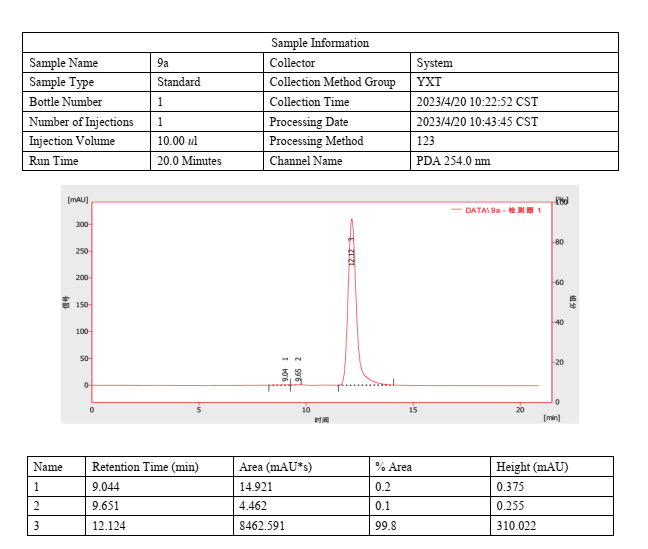


**Figure S9.** HPLC chromatograph of **9a**


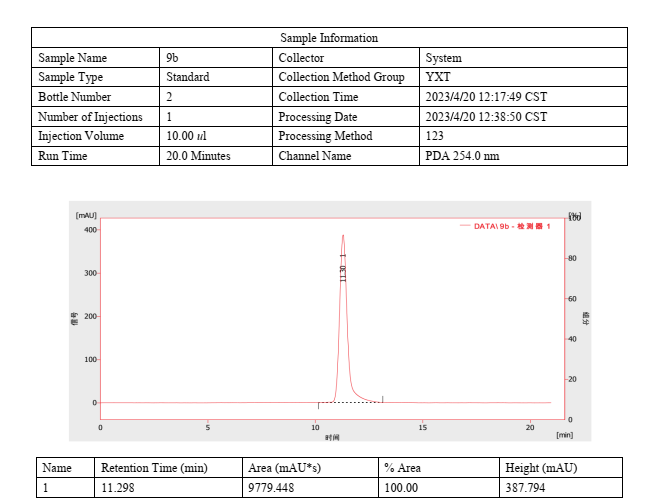


**Figure S10.** HPLC chromatograph of **9b**

**11. Copies of spectra of compounds 5a, 8a, 9b, 9a, 9b, 12a, 14a, 14b, 18a, 20a, and 20b**


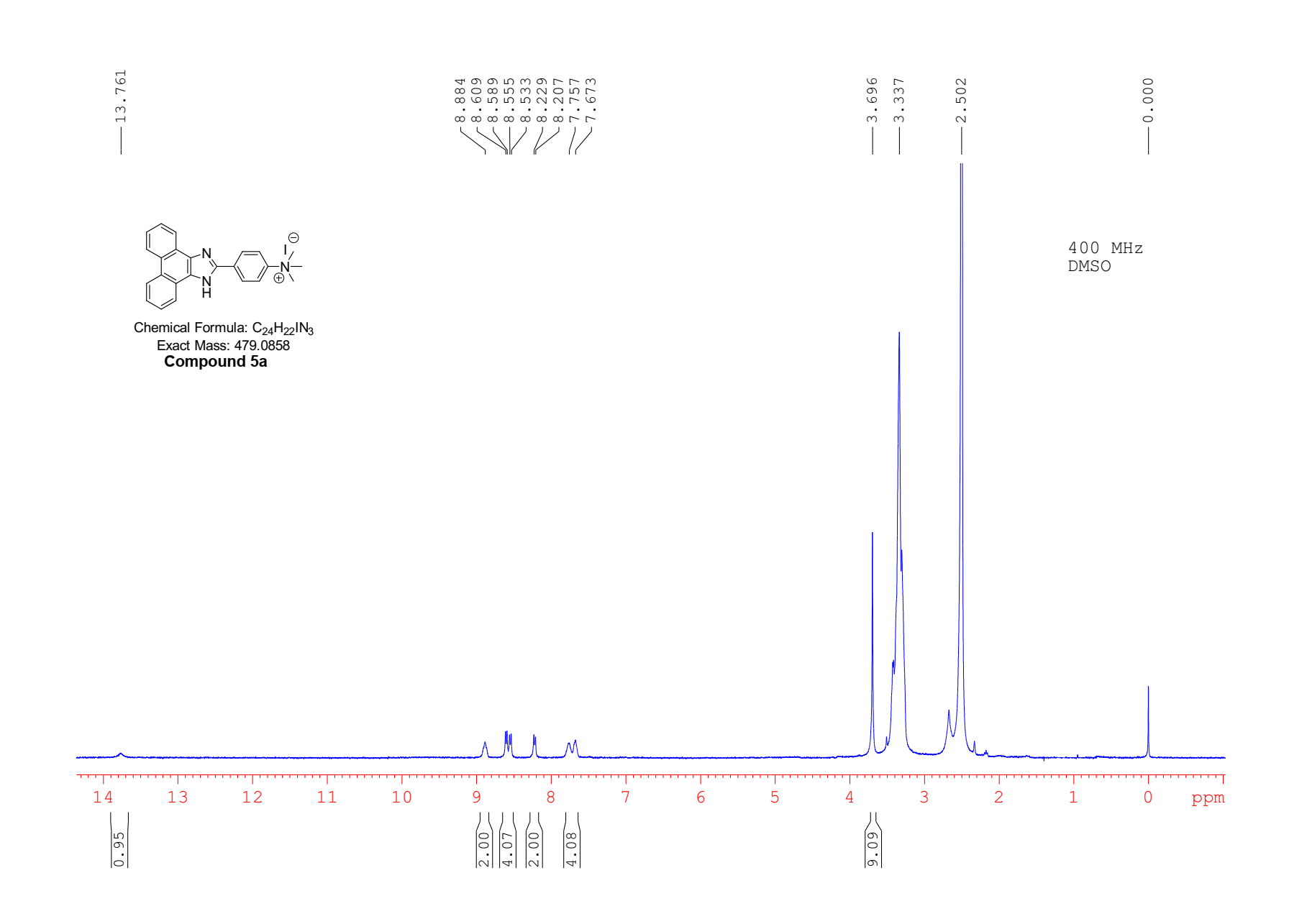


**Figure S11.** ^1^H NMR spectrum of **5a**


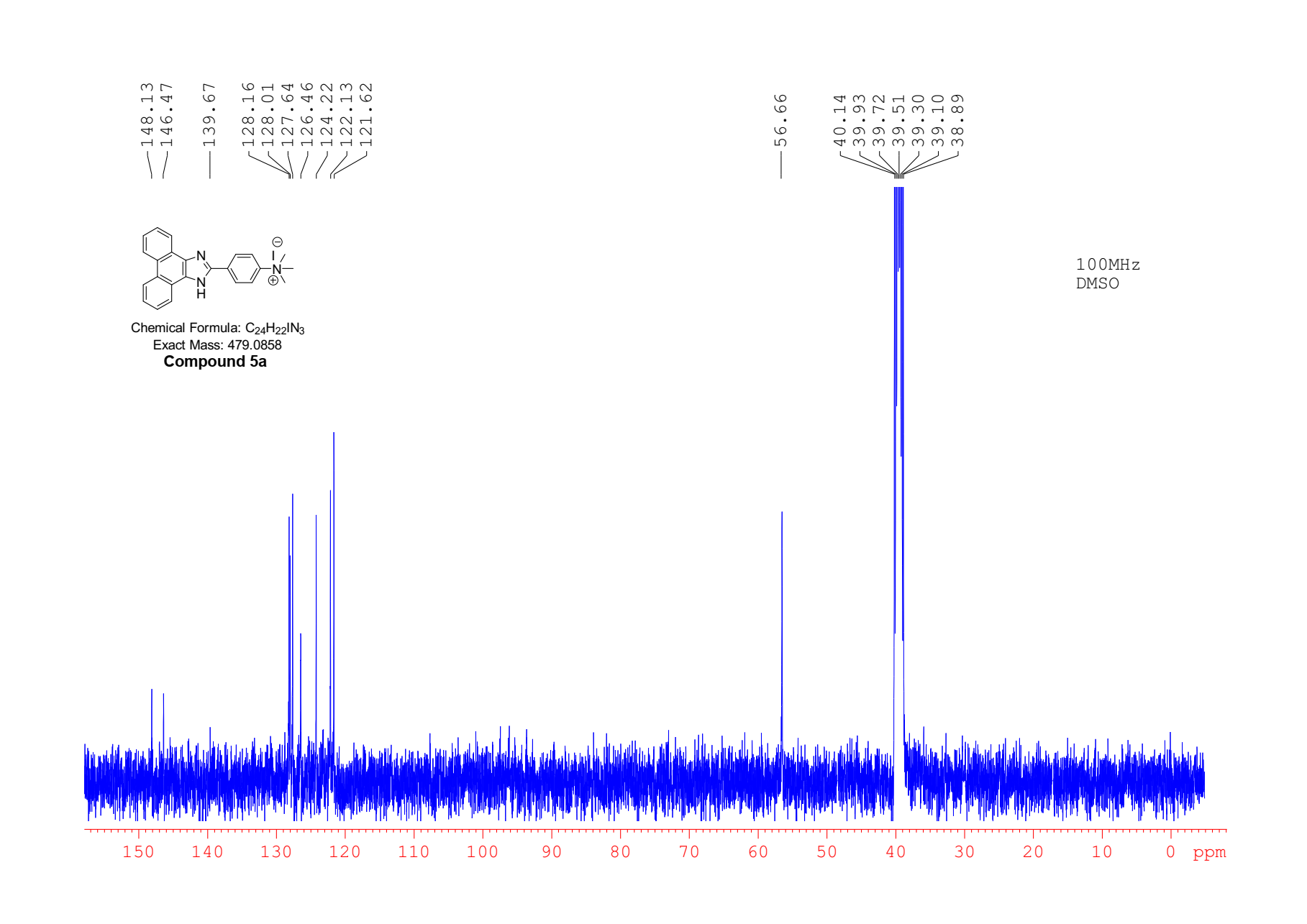


**Figure S12.** ^13^C NMR spectrum of **5a**


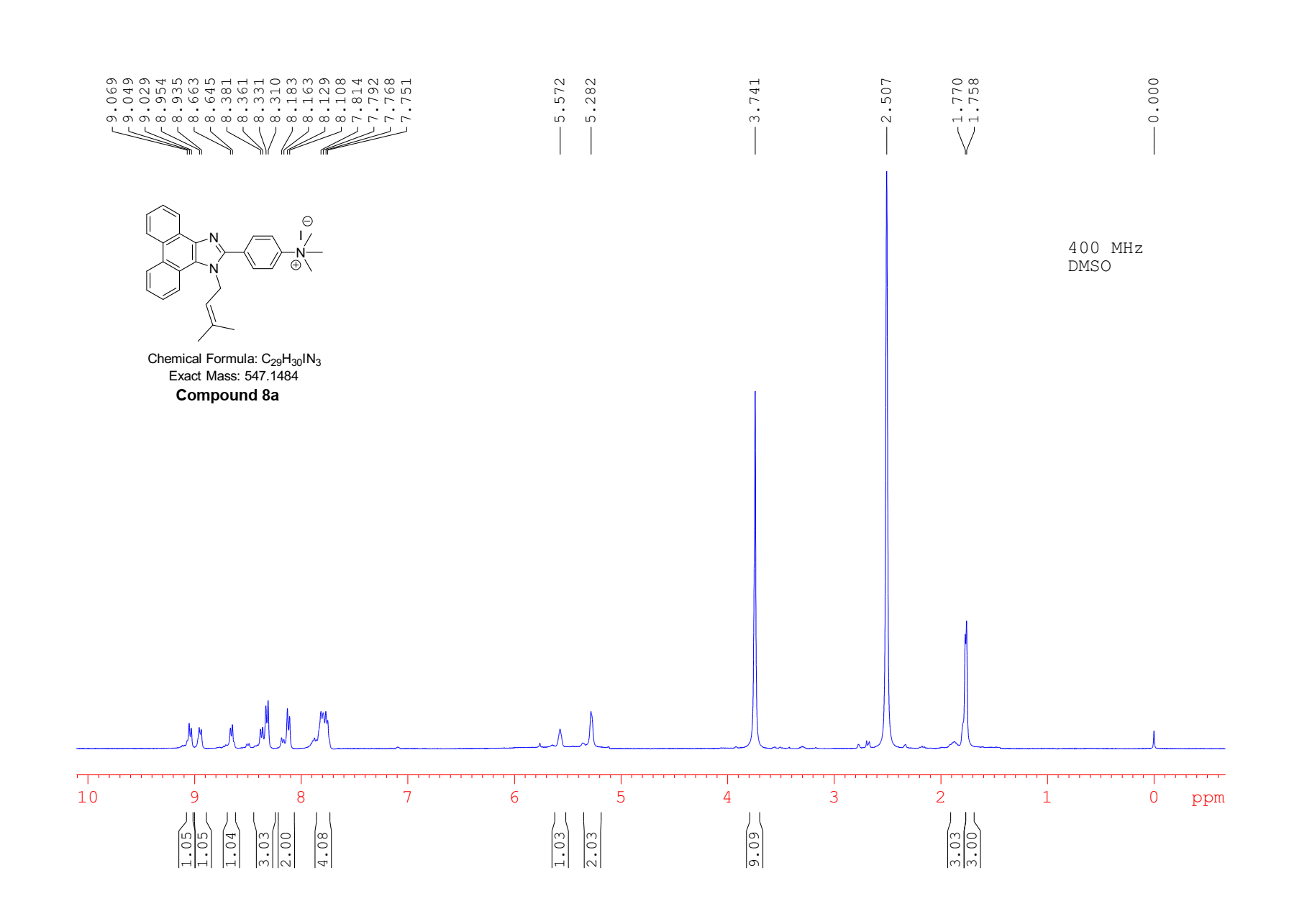


**Figure S13.** ^1^H NMR spectrum of **8a**


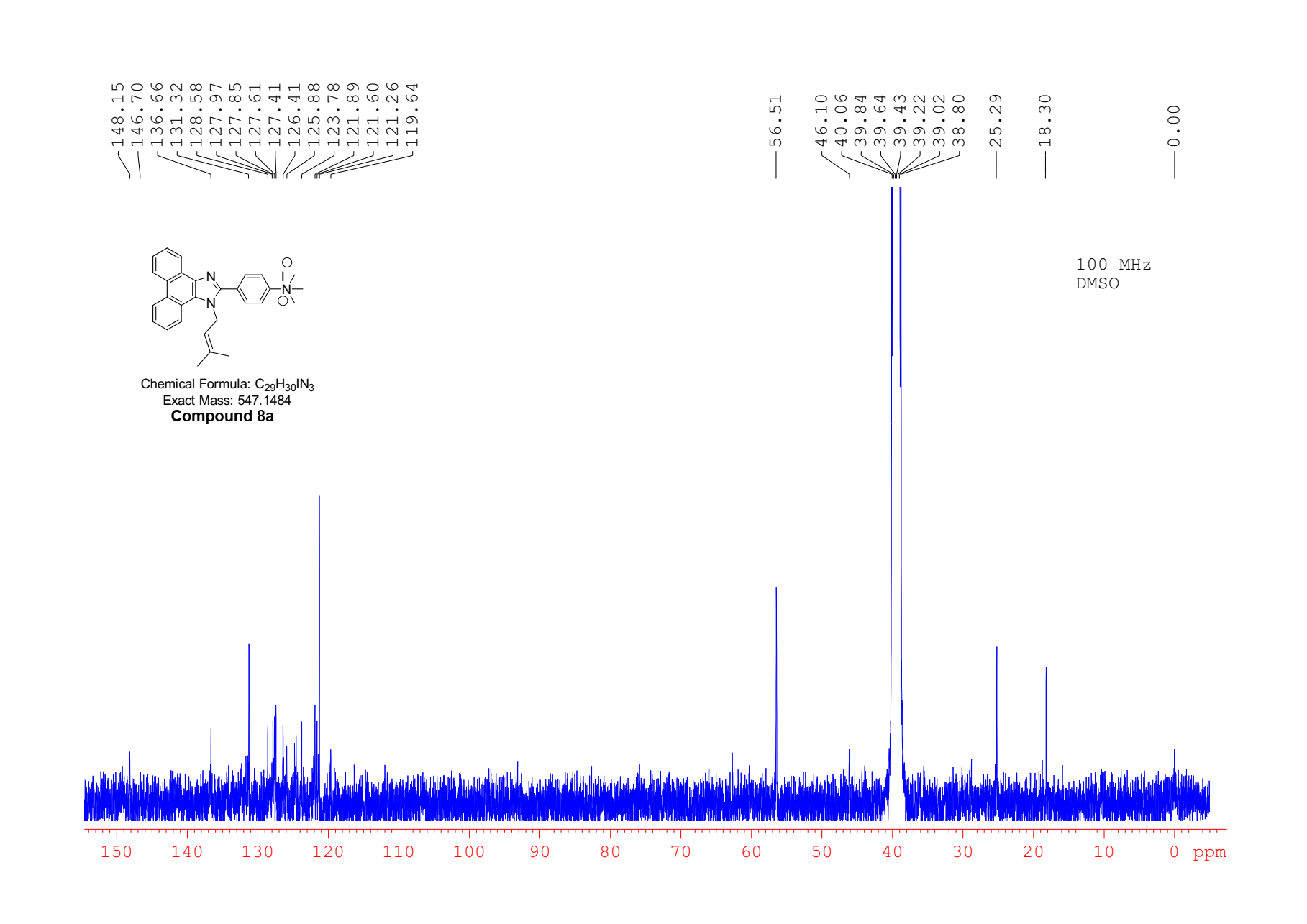


**Figure S14.** ^13^C NMR spectrum of **8a**


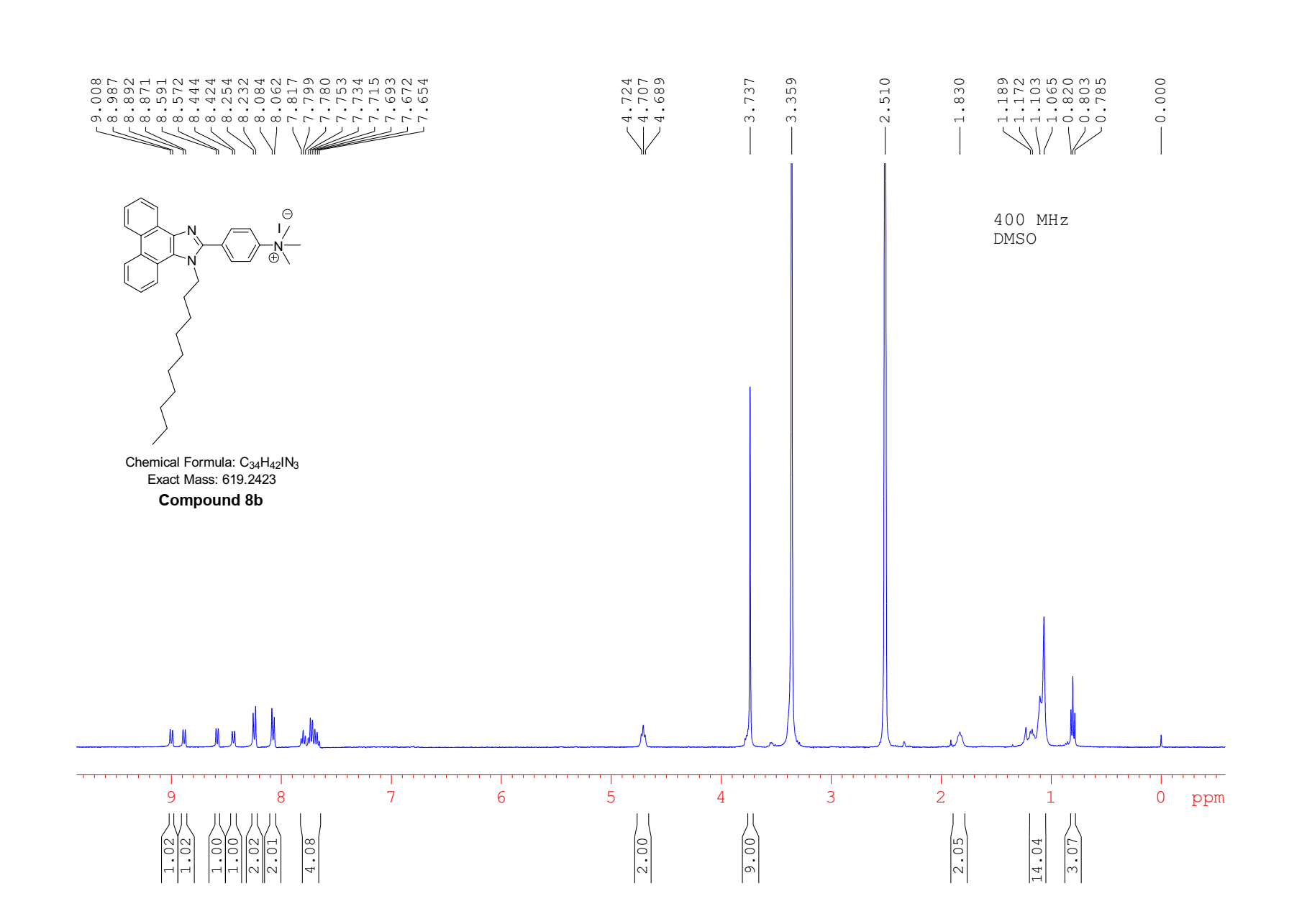


**Figure S15.** ^1^H NMR spectrum of **8b**


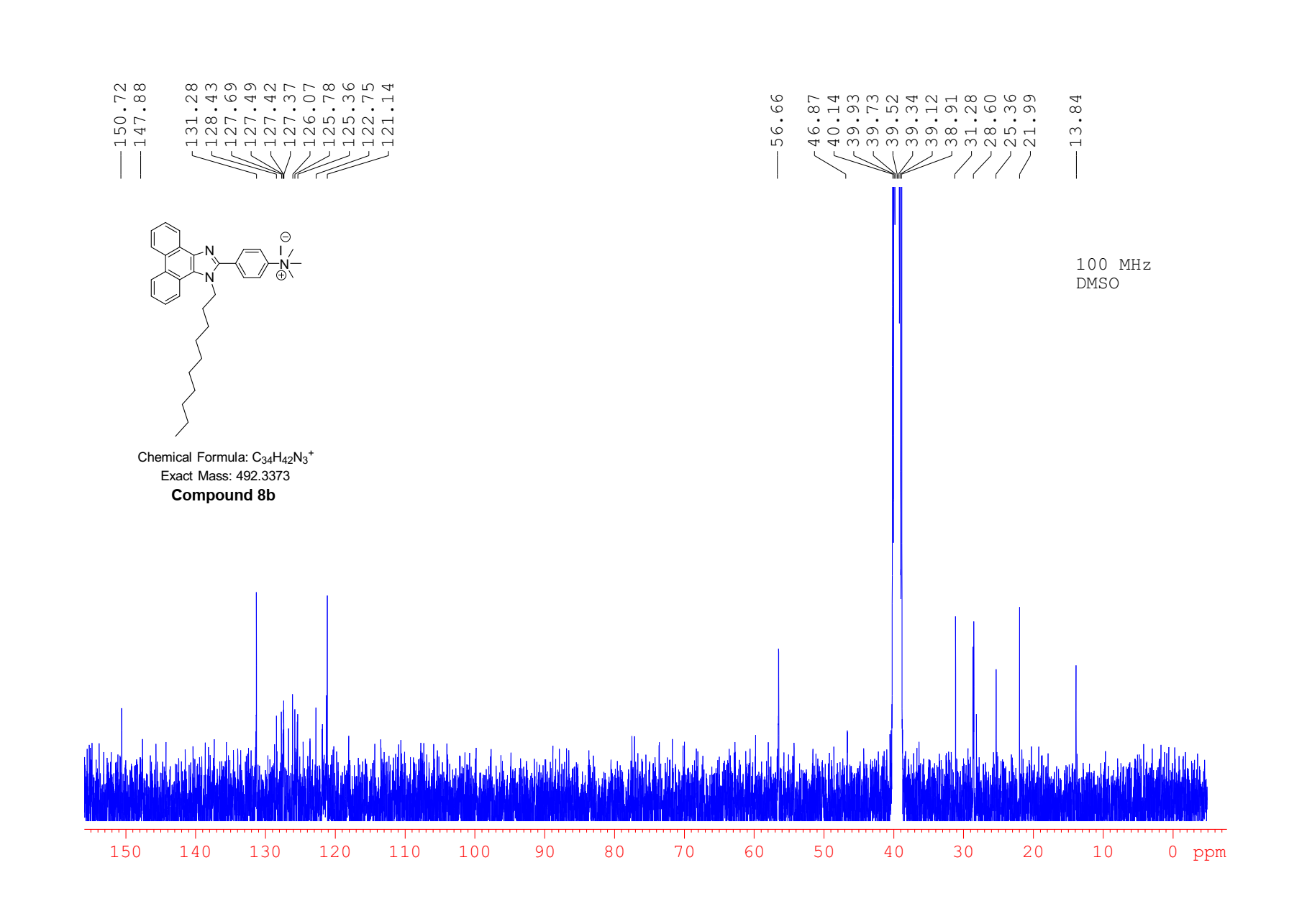


**Figure S16.** ^13^C NMR spectrum of **8b**


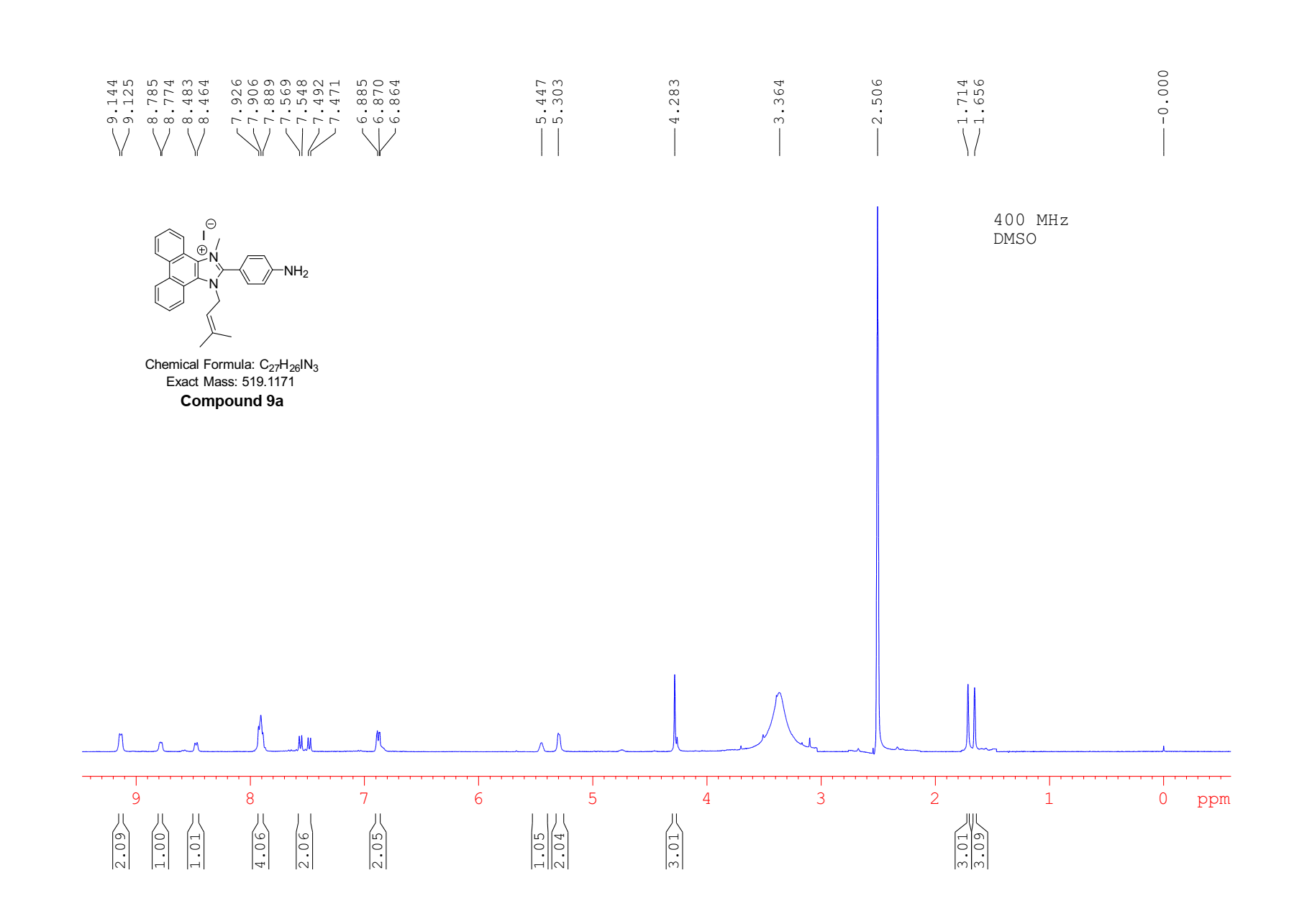
 **Figure S17.** ^1^H NMR spectrum of **9a**


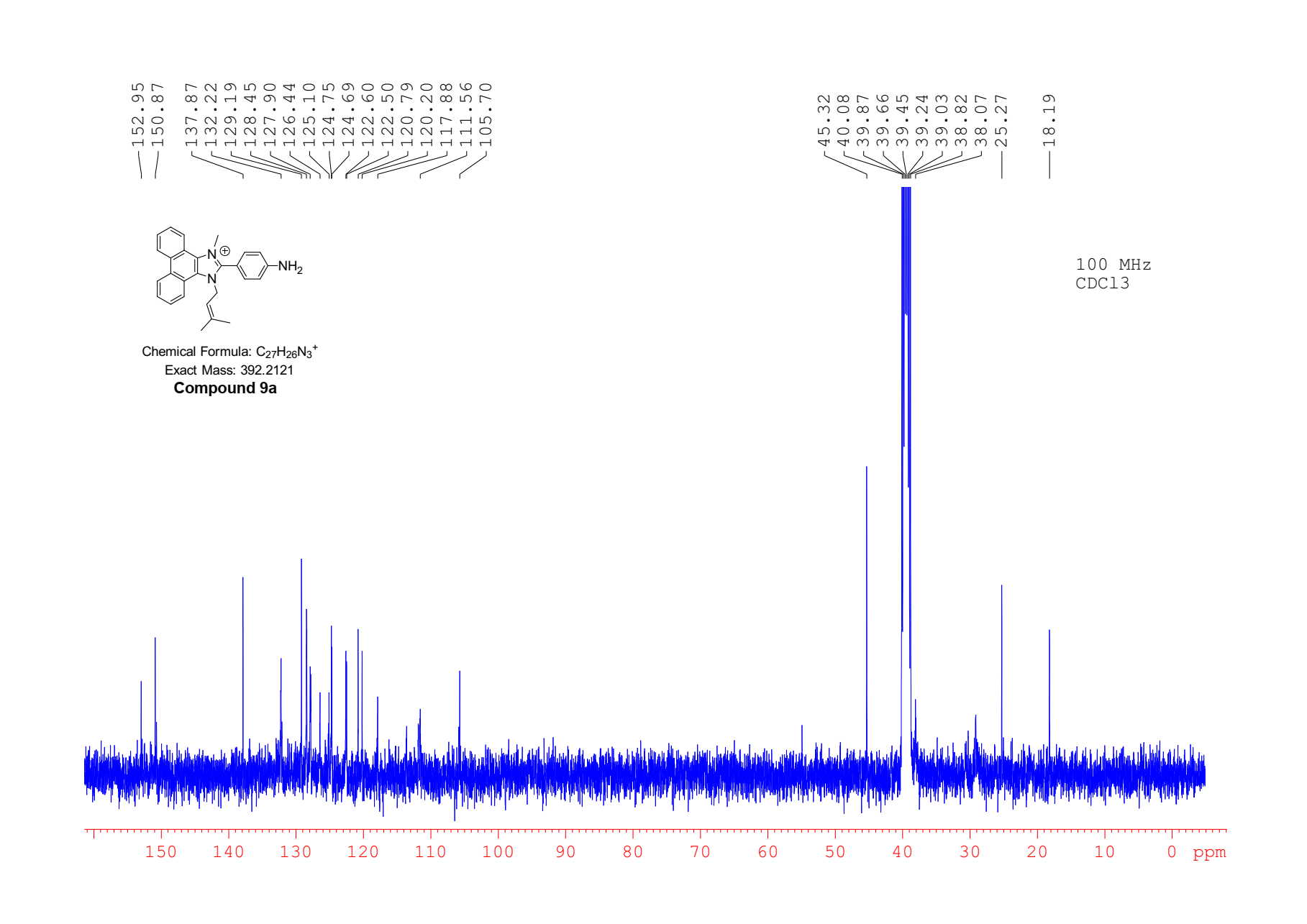
 **Figure S18.** ^13^C NMR spectrum of **9a**


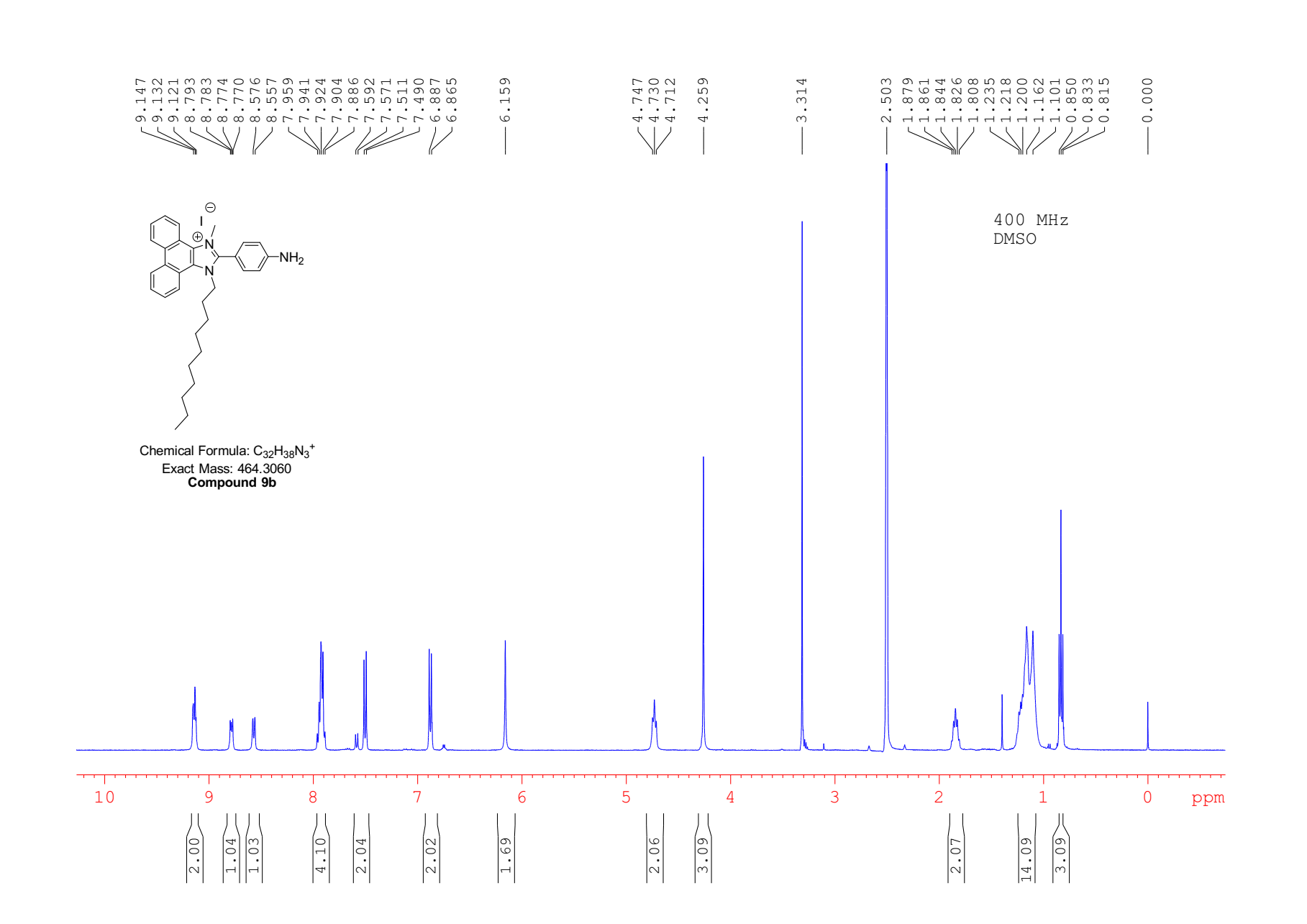


**Figure S19.** ^1^H NMR spectrum of **9b**


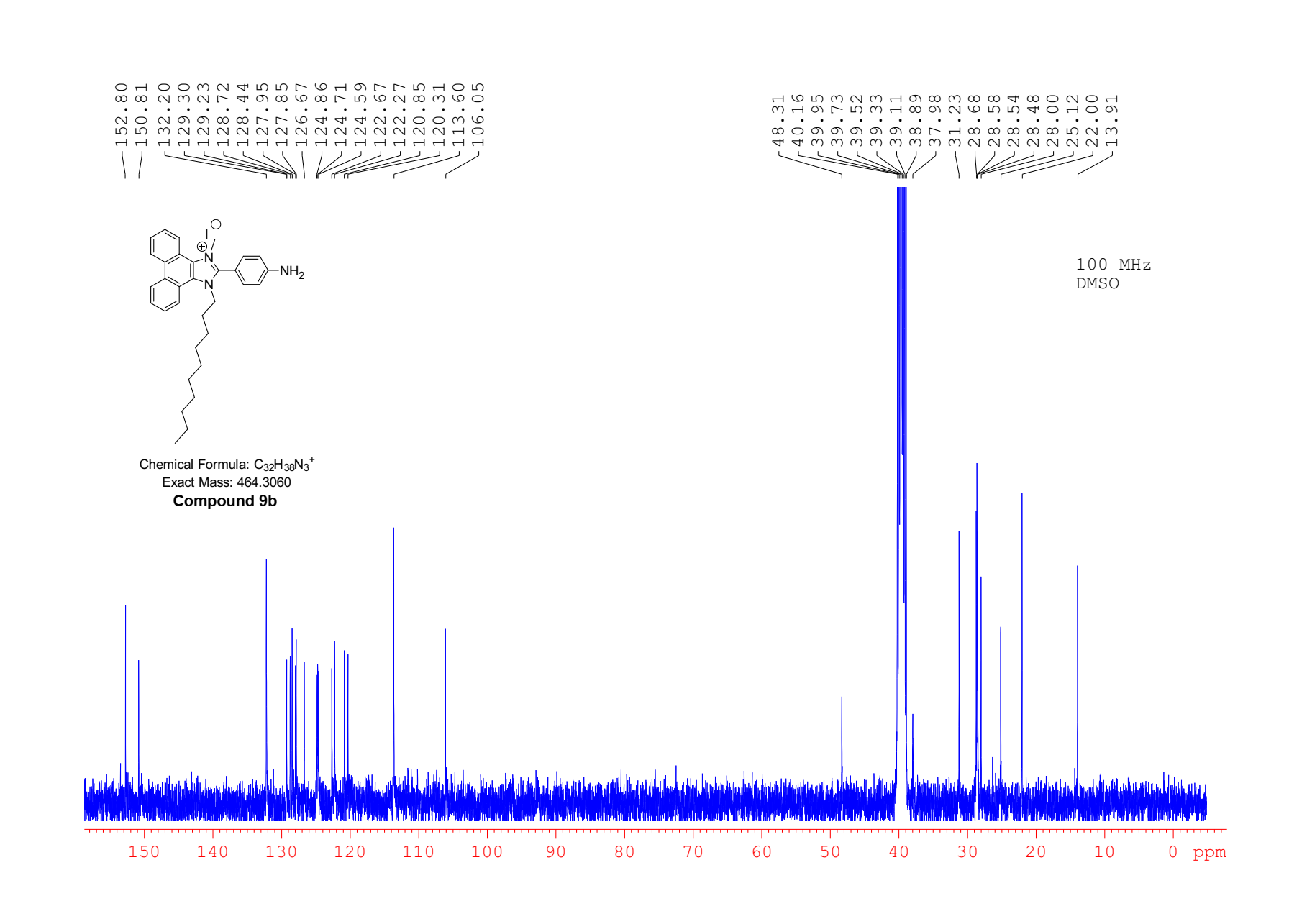


**Figure S20.** ^13^C NMR spectrum of **9b**


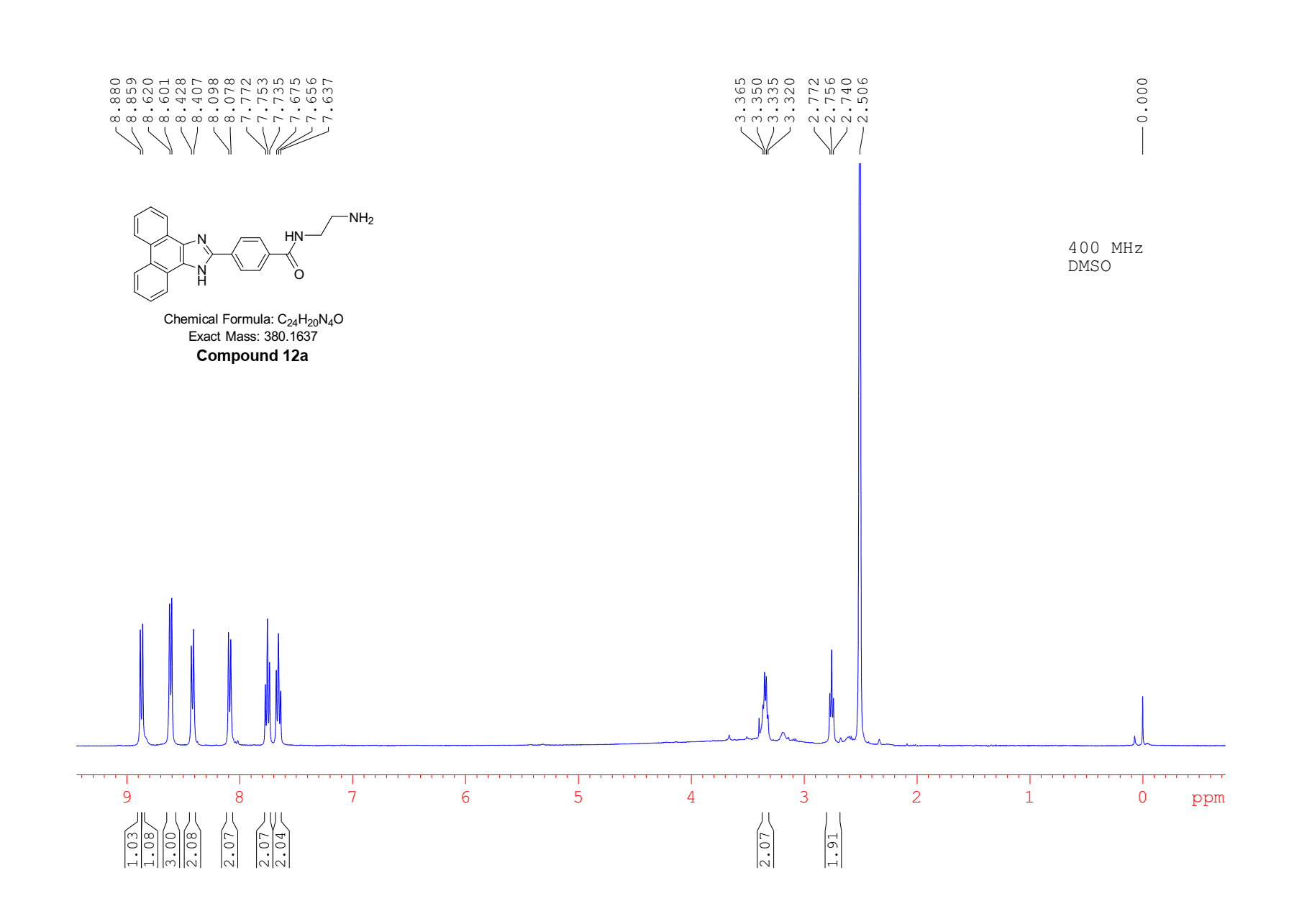


**Figure S21.** ^1^H NMR spectrum of **12a**


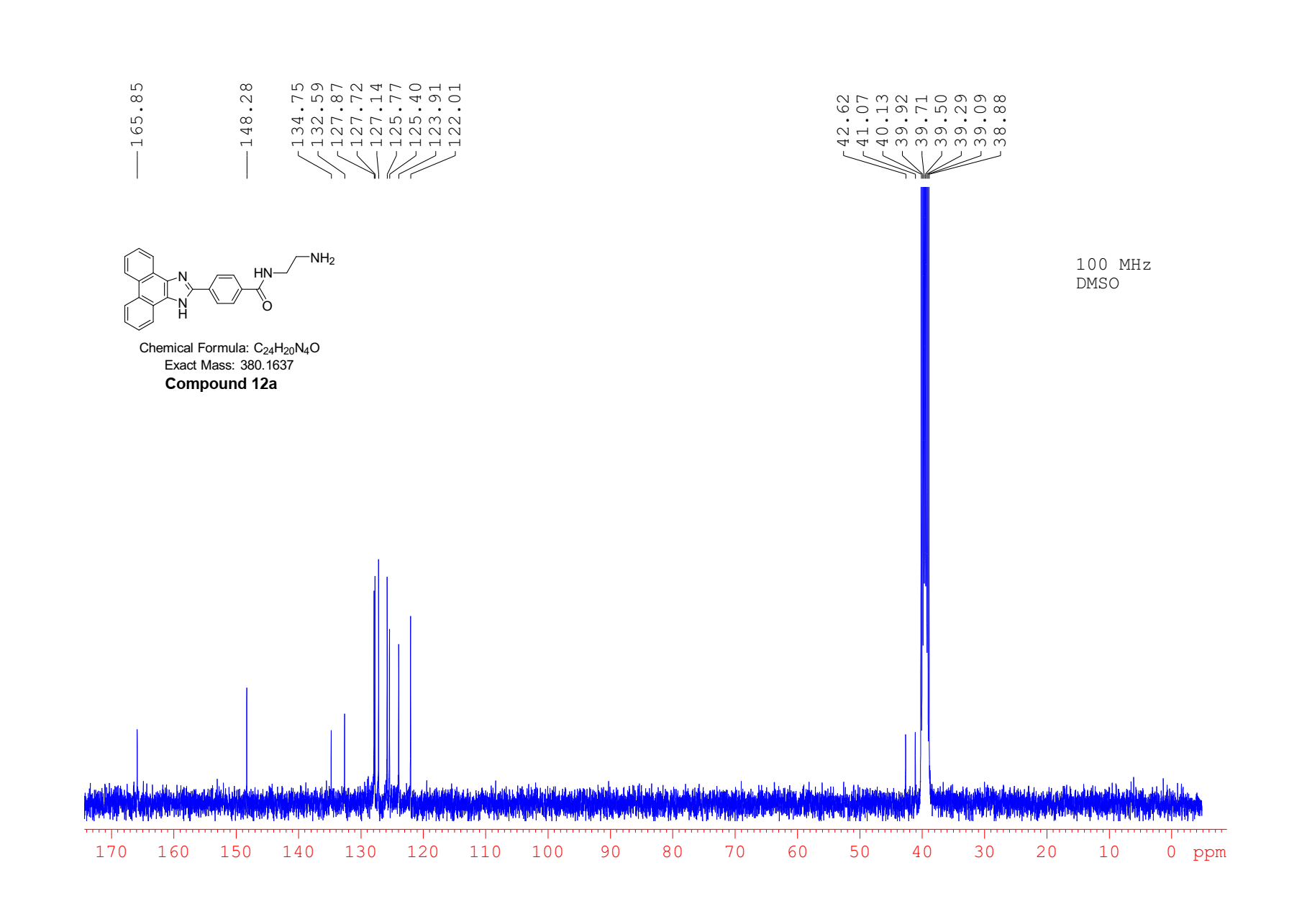


**Figure S22.** ^13^C NMR spectrum of **12a**


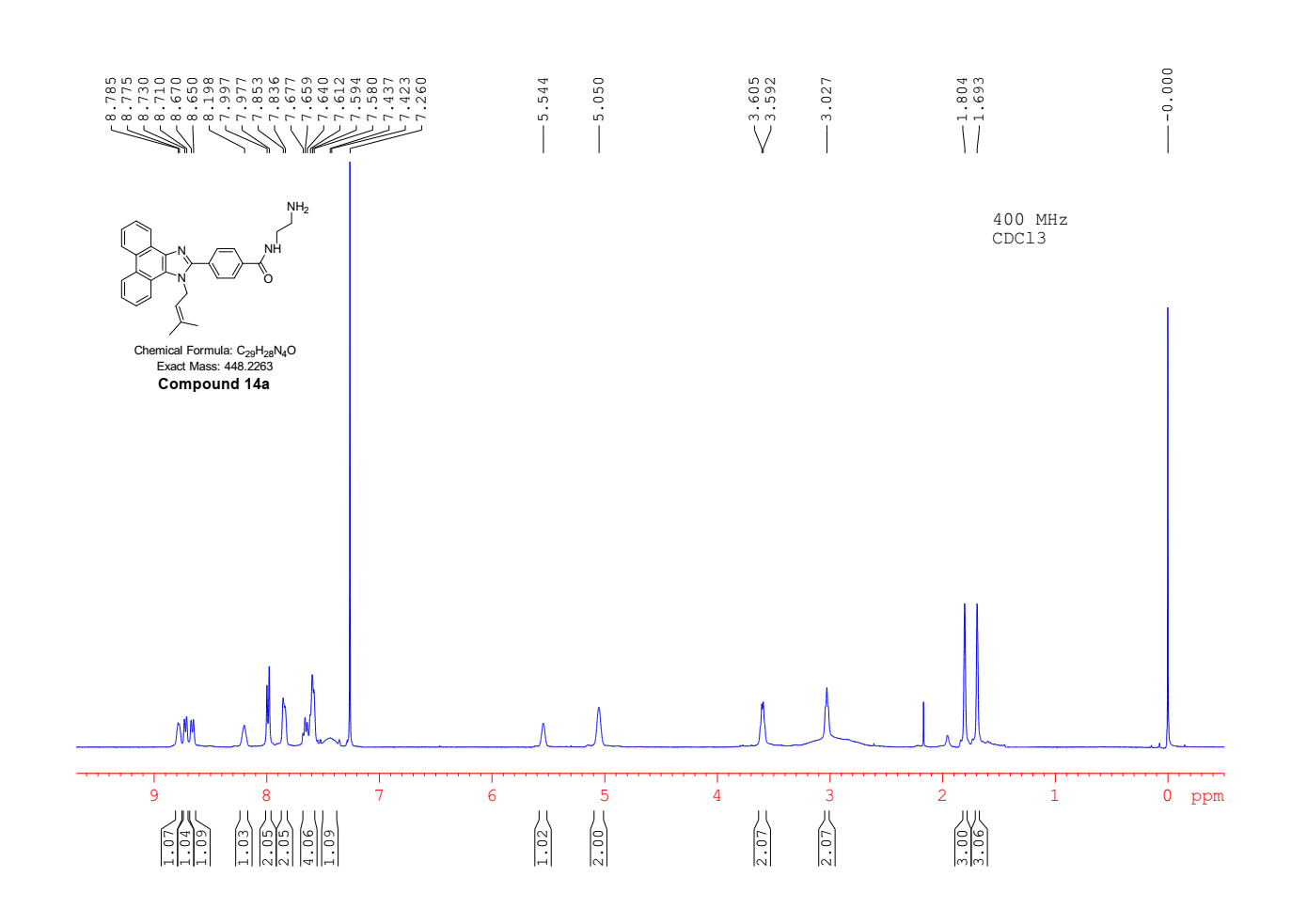


**Figure S23.** ^1^H NMR spectrum of **14a**


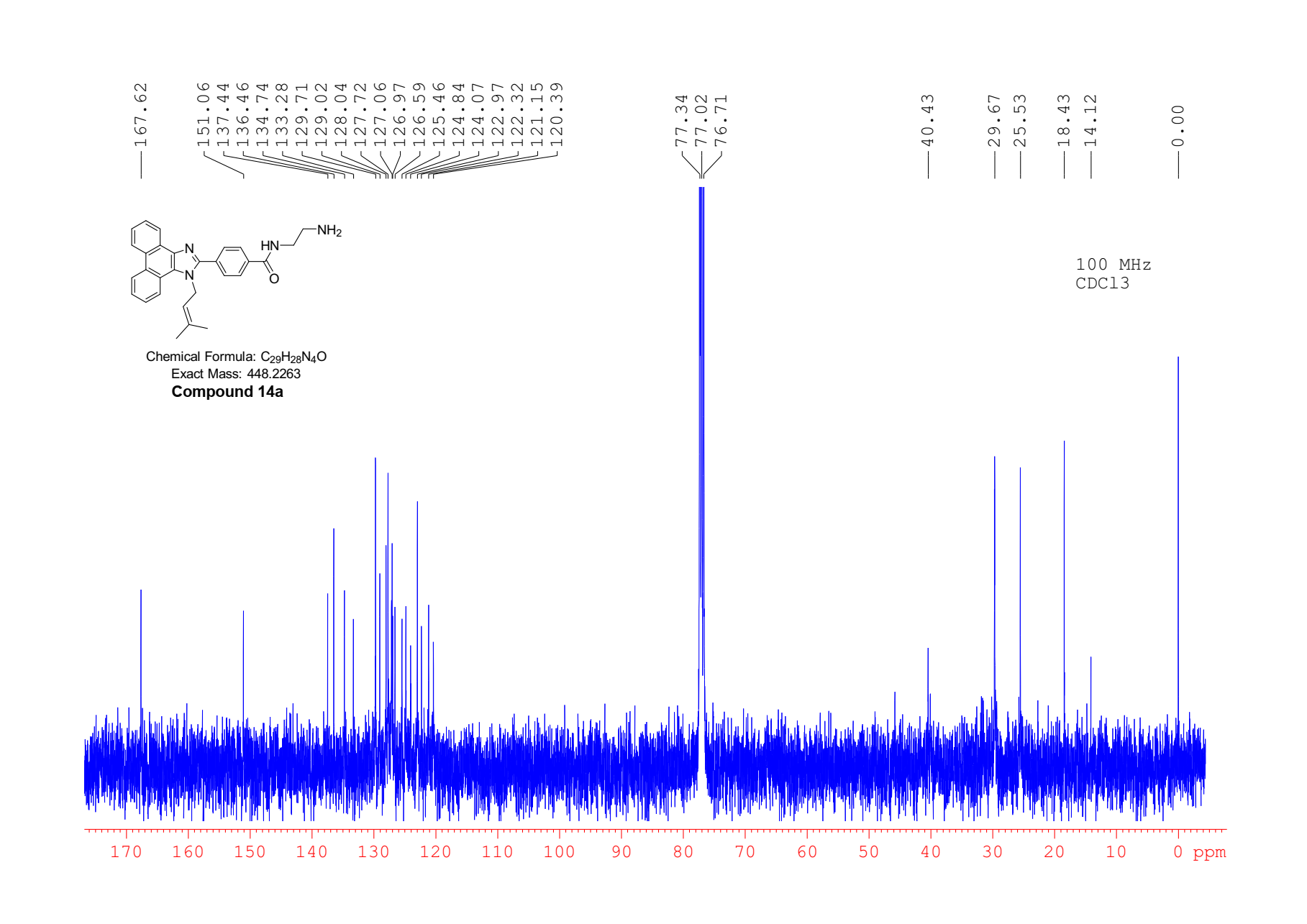


**Figure S24.** ^13^C NMR spectrum of **14a**


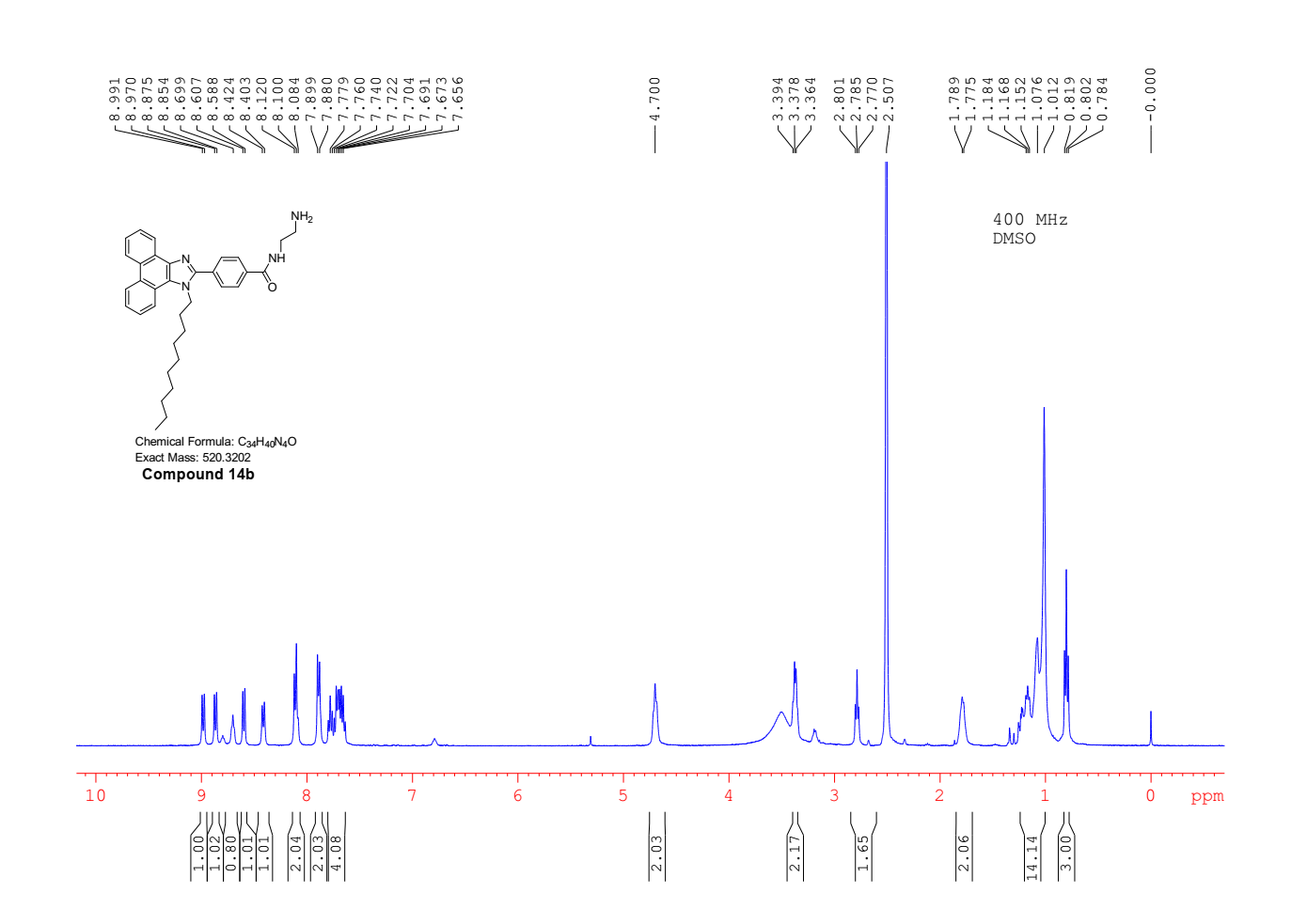


**Figure S25.** ^1^H NMR spectrum of **14b**


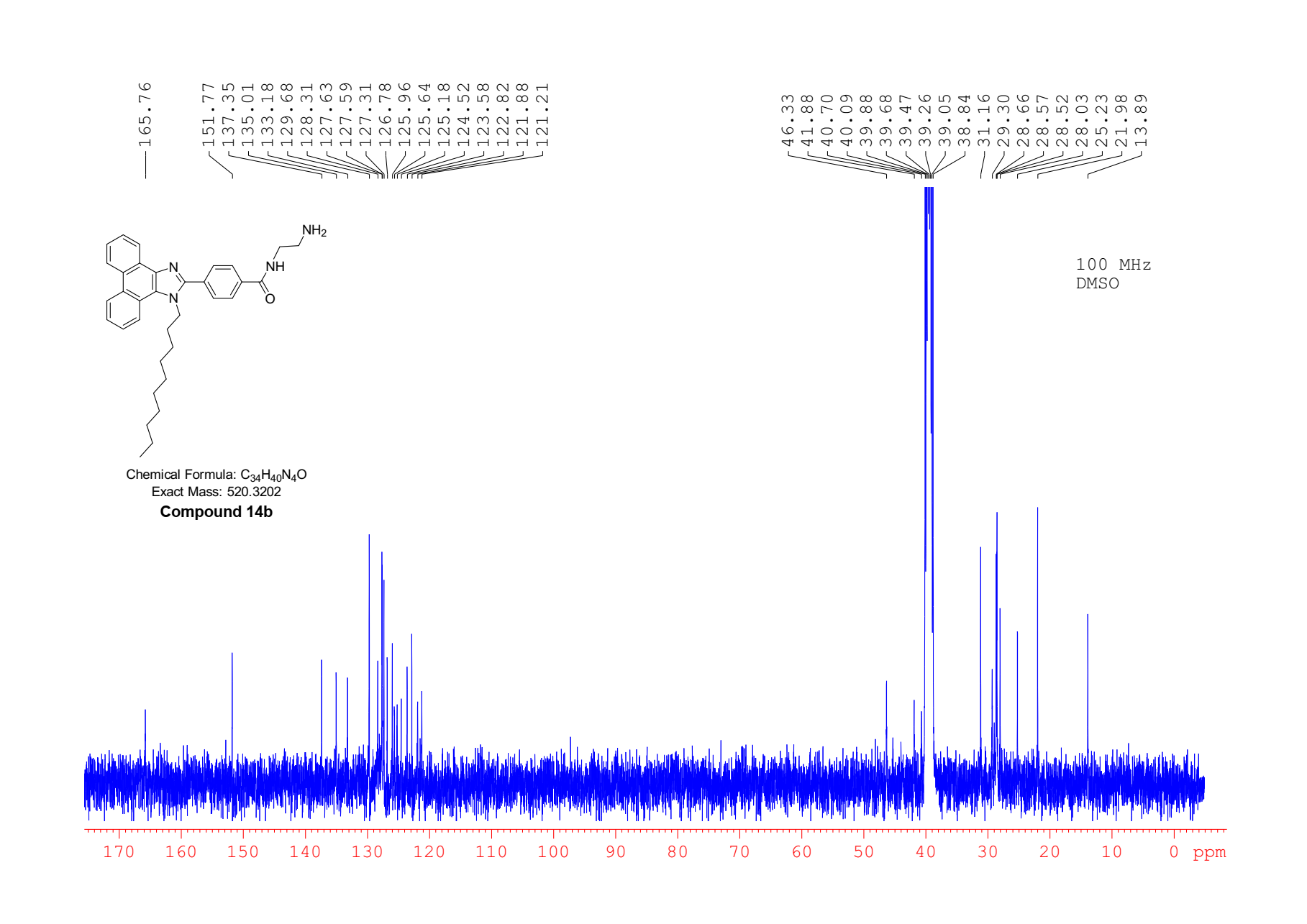


**Figure S26.** ^13^C NMR spectrum of **14b**


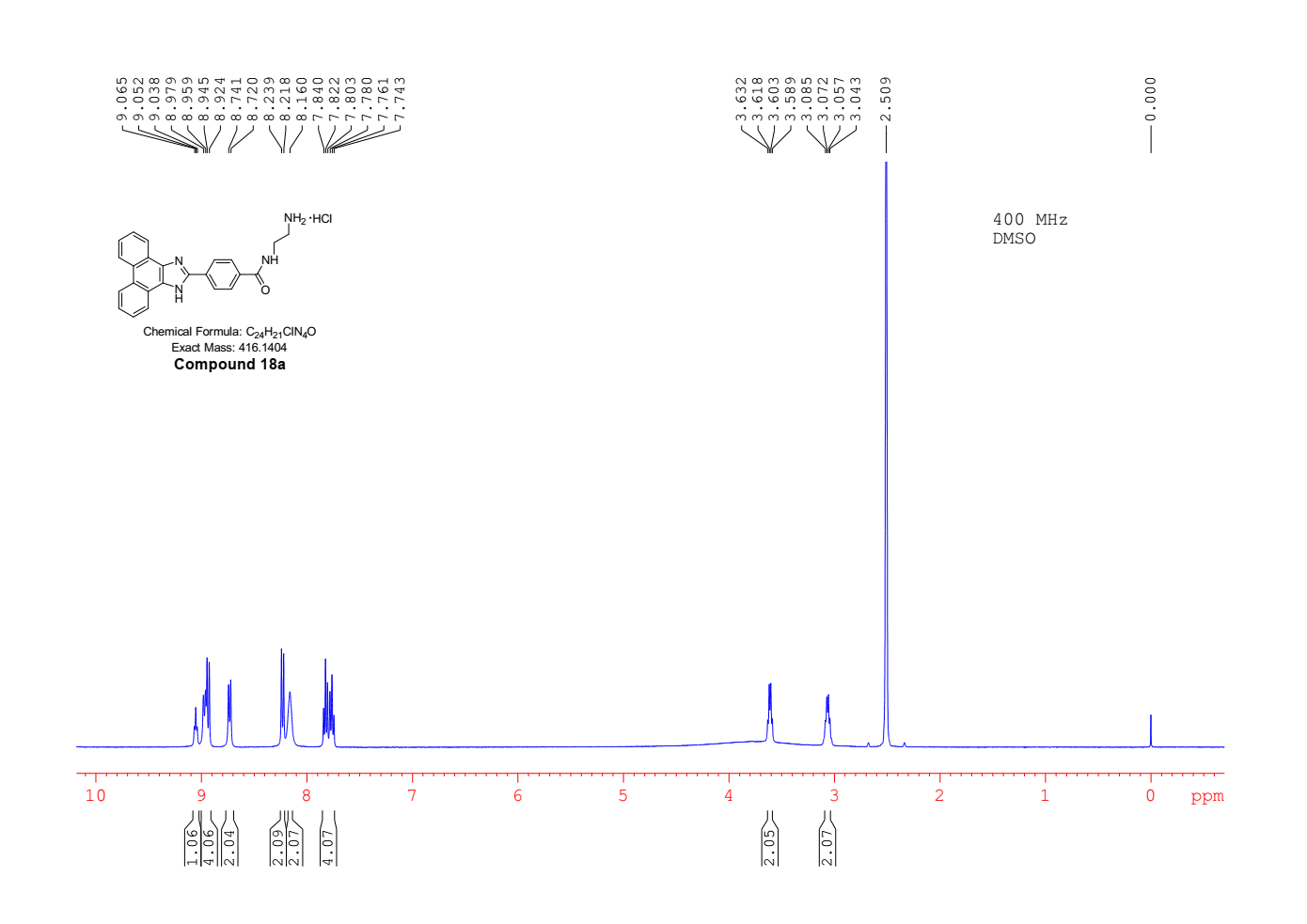


**Figure S27.** ^1^H NMR spectrum of **18a**


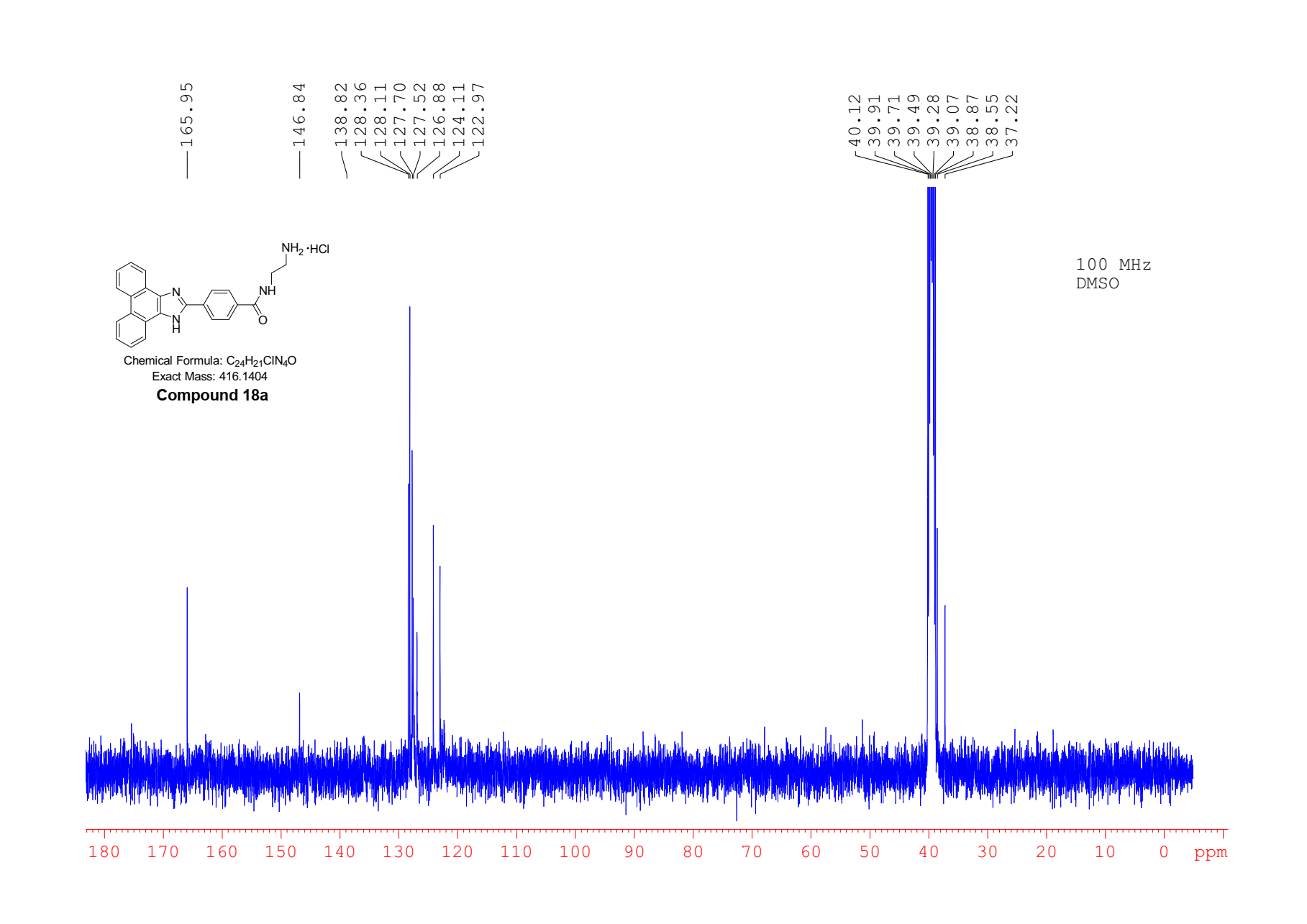


**Figure S28.** ^13^C NMR spectrum of **18a**


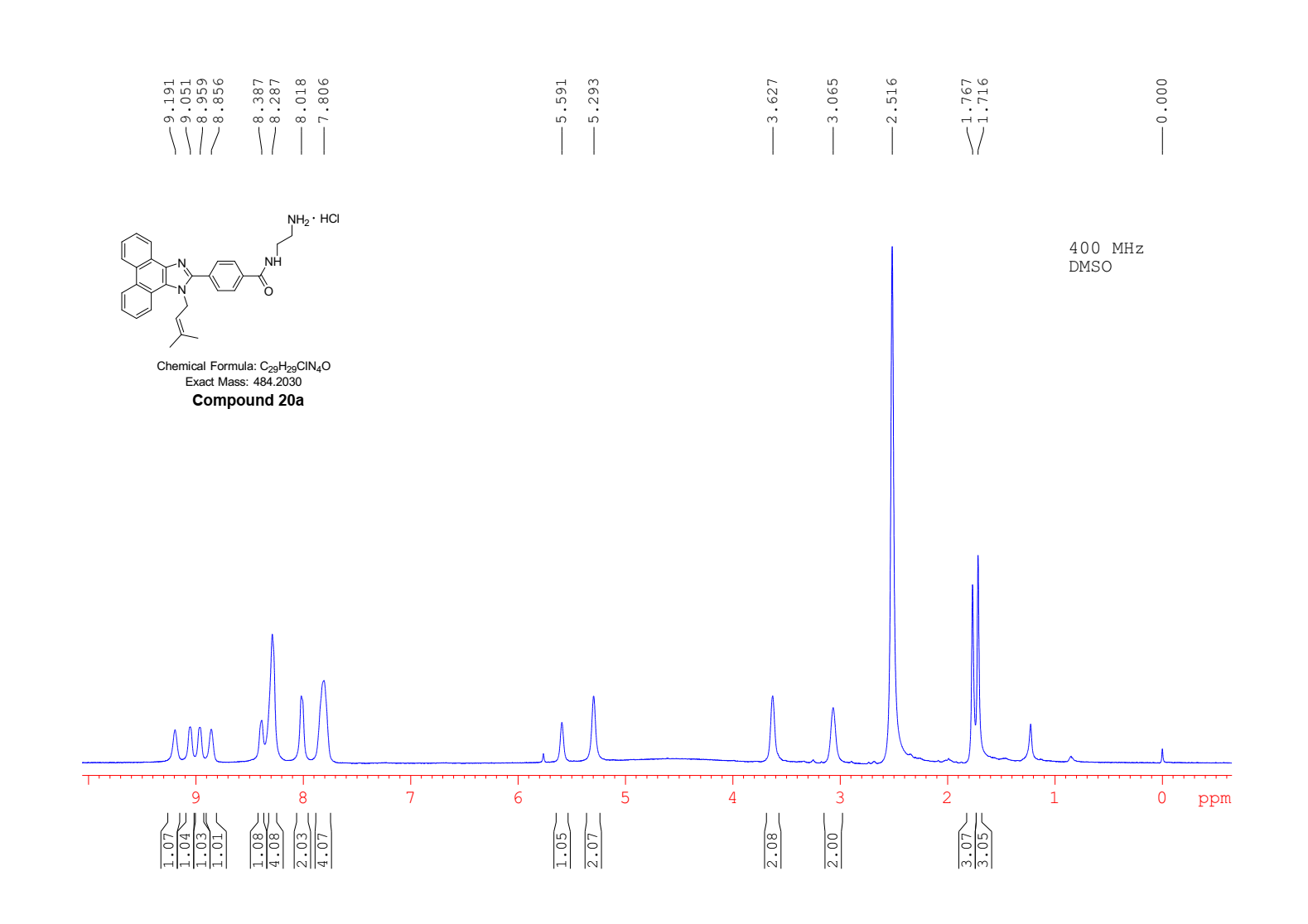


**Figure S29.** ^1^H NMR spectrum of **20a**


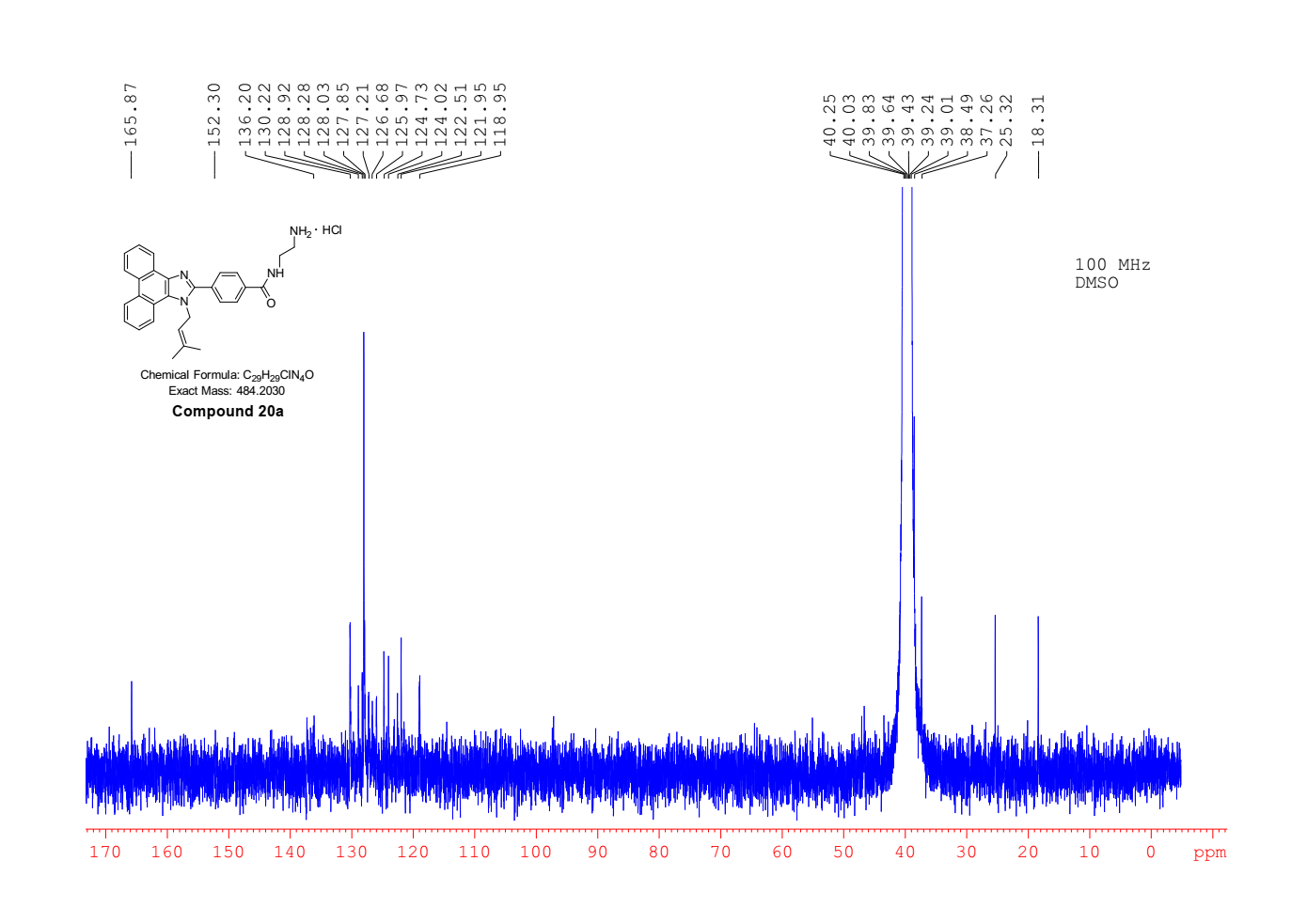


**Figure S30.** ^13^C NMR spectrum of **20a**


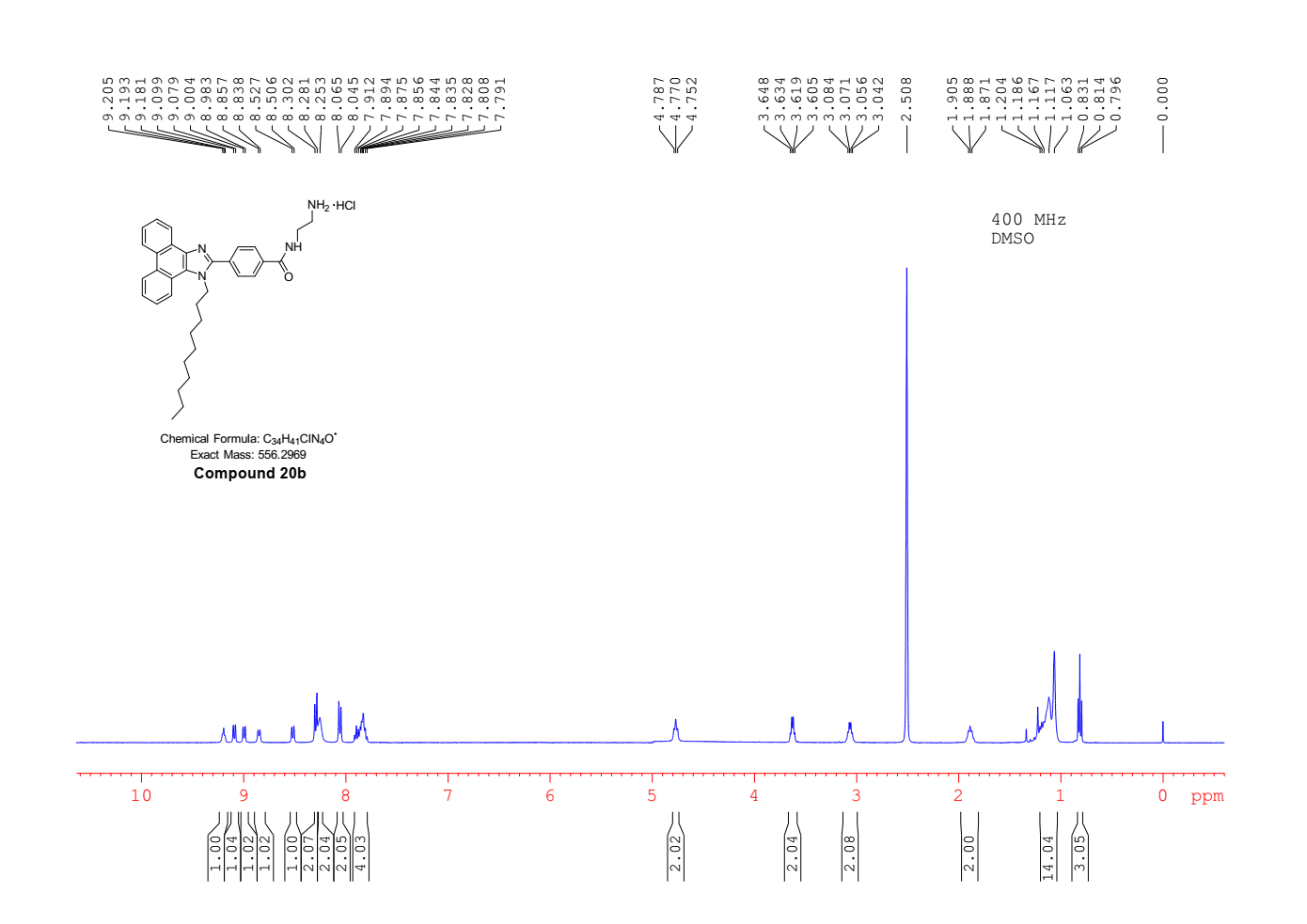


**Figure S31.** ^1^H NMR spectrum of **20b**


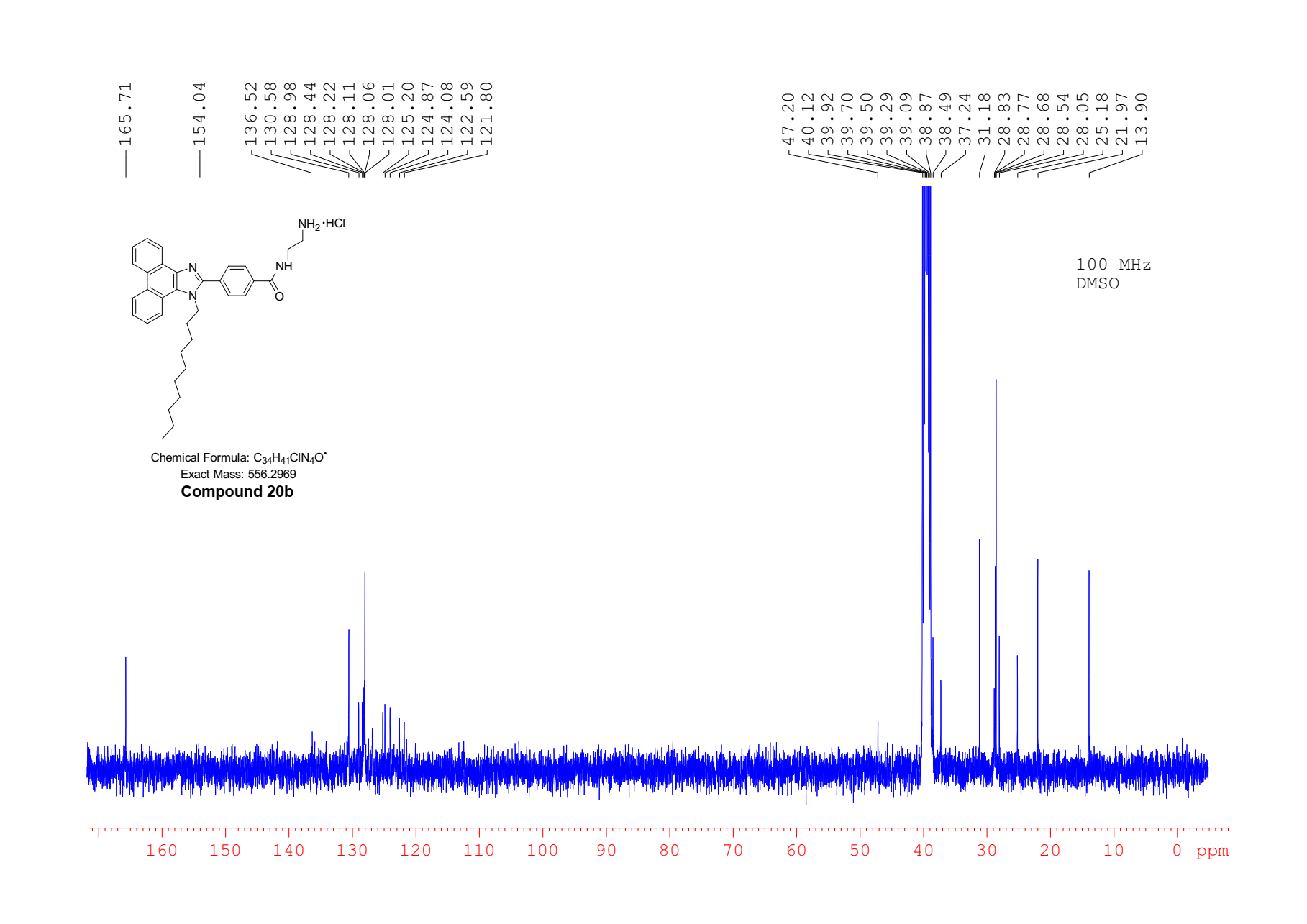


**Figure S32.** ^13^C NMR spectrum of **20b**
